# Rheumatoid arthritis burden in 129 low- and middle-income countries from 1990 to 2023

**DOI:** 10.1016/j.isci.2026.116881

**Published:** 2026-07-21

**Authors:** Hongnian Wang, Mingyang Zhang, Xiong Ke, Zichao Wang, Lijuan Wu

**Affiliations:** 1 Key Laboratory of Digital-Intelligent Disease Surveillance and Health Governance, North Sichuan Medical College, Nanchong, China; 2 School of Social Sciences, Henan Normal University, Xinxiang, China; 3 Department of Epidemiology and Statistics, Institute of Basic Medical Sciences Chinese Academy of Medical Sciences, School of Basic Medicine Peking Union Medical College, Beijing, China; 4 Institute of Sciences in Emergency Medicine, Department of Emergency Medicine, Guangdong Provincial People’s Hospital (Guangdong Academy of Medical Sciences), Southern Medical University, Guangzhou, China; 5 Medical Research Institute, Guangdong Provincial People’s Hospital (Guangdong Academy of Medical Sciences), Southern Medical University, Guangzhou, China

**Keywords:** arthritis, rheumatoid, developing countries, global burden of disease, health equity, health services accessibility, mortality

## Abstract

Rheumatoid arthritis (RA) causes substantial disability in low- and middle-income countries (LMICs), which account for most of the global population. Using Global Burden of Disease (GBD) 2023 data, we assessed RA prevalence, incidence, mortality, and disability-adjusted life-year (DALY) rates across 129 LMICs from 1990 to 2023 and projected trends to 2050. Countries were stratified by gross national income (GNI) into low-income, lower-middle-income, and upper-middle-income groups. In 2023, RA affected 12.6 million individuals, with prevalence in upper-middle-income countries being 4.1-fold higher than in low-income countries. Death rates converged across income groups, while absolute inequality in prevalence widened and inequality in mortality narrowed. Projections to 2050 indicated declining mortality in low-income settings but increasing in middle-income countries. Therefore, it is necessary to improve diagnostic capacity in low-income settings and medication affordability in middle-income countries.

## Highlights

• RA prevalence was highest in upper-middle-income countries; death rates were similar
• RA morbidity peaked in Latin America; mortality was highest in South Asia and Africa
• GNI per capita was correlated with RA morbidity but not with death rates
• Death rates are projected to rise in middle-income but decline in low-income groups

## Graphical abstract

**Figure:**
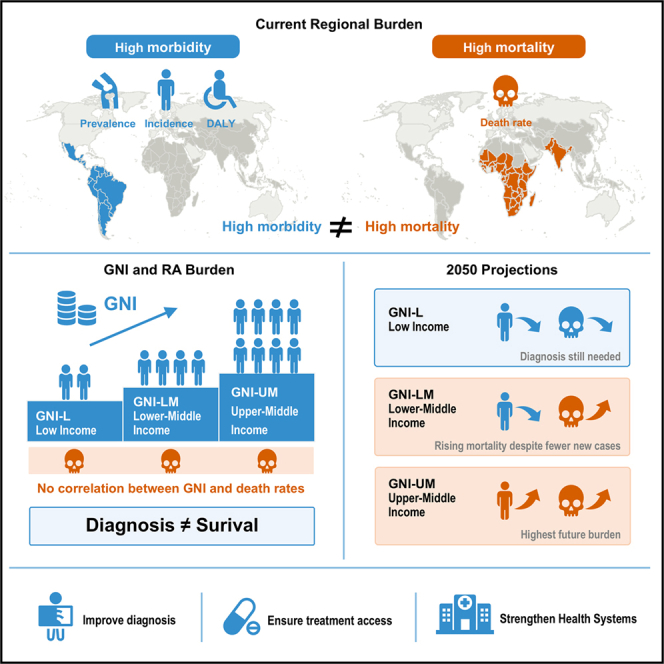

## Teaser

health sciences; medicine; medical specialty; internal medicine; rheumatology; public health

## Introduction

Rheumatoid arthritis (RA) is a systemic autoimmune disease affecting approximately 18 million people globally.^1^^,^^2^ As a leading cause of disability, it imposes a substantial burden due to productivity loss and high treatment costs^3^^,^^4^ and is associated with an approximately 30% higher death rate compared with that of the general population.^5^^,^^6^^,^^7^ However, this burden is not equitably distributed. Low- and middle-income countries (LMICs), accounting for over 80% of the global population,^8^^,^^9^ face the dual pressure of severely constrained healthcare resources and rapid demographic shifts. Despite this large population size, evidence on RA burden in LMICs remains scarce.^10^ This data gap stems from a critical policy failure: musculoskeletal disorders, including RA, are excluded from the global non-communicable diseases (NCD) agenda.^11^^,^^12^ This exclusion directly constrains the prioritization and allocation of resources for RA in national health strategies, especially in LMICs.^13^^,^^14^ Therefore, understanding the disease burden patterns and trends of RA in these nations is critical to developing equitable health policies.

Reliable estimates are essential for policy planning; yet, country-specific evidence remains fragmented. The Global Burden of Disease (GBD) study provides the primary estimates of RA burden across countries and time periods.^15^^,^^16^ However, previous analyses have largely aggregated data globally, regionally, or by socio-demographic index (SDI),^1^^,^^17^^,^^18^^,^^19^^,^^20^ thereby overlooking heterogeneity within LMICs, particularly the substantial differences across income tiers in healthcare accessibility, medication coverage, and disease management capacity. Moreover, these previous analyses used data only up to 2021 and, therefore, did not include the updated GBD 2023 estimates extending through 2023.^10^ Several evidence gaps remain. It is still unclear how RA burden trajectories differ across gross national income (GNI) strata within LMICs, how GNI per capita is ecologically associated with country-level RA burden, and how absolute and relative inequalities have changed over time. These gaps limit the identification of income-related burden patterns and inequity hotspots within LMICs, as well as the anticipation of future service needs through long-term projections.

To address these evidence gaps, we analyzed RA burden across 129 LMICs from 1990 to 2023 using the most recent GBD 2023 data. Temporal trends in prevalence rates, incidence rates, death rates, and disability-adjusted life-year (DALY) rates were examined across three GNI groups (low-income [GNI-L], lower-middle-income [GNI-LM], and upper-middle-income [GNI-UM]) to characterize income-based variations within LMICs. Country-level Spearman correlation analysis was employed to assess ecological associations between GNI per capita and disease burden. Health inequalities were evaluated using the slope index of inequality (SII) for absolute inequality and the concentration index for relative inequality. Moreover, autoregressive integrated moving average (ARIMA) models were applied to project burden trajectories to 2050 to support long-term healthcare planning, resource allocation, rheumatology workforce preparation, and preventive policy development.

## Results

### Overall burden trends by GNI

RA burden increased substantially across LMICs, with DALYs, prevalent cases, incident cases, and deaths all rising between 1990 and 2023. The number of prevalent cases showed the largest relative increase, rising from 4.51 million (95% uncertainty interval [UI]: 3.84–5.29) in 1990 to 12.61 million (95% UI: 10.97–14.69) in 2023 (+179.6%) (Table 1). Across income strata, GNI-LM showed the most pronounced growth in absolute burden, most notably for prevalent cases, which increased by 239.5% from 1.32 to 4.48 million. Corresponding increases in incident cases, DALYs, and deaths are shown in Table 1.

**Table 1:** Burden of rheumatoid arthritis in 129 LMICs by GNI from 1990 to 2023

Metric and location	Cases, absolute count		Trend, 1990–2023	
	1990 (95% UI)	2023 (95% UI)	PC (%)^a^	EAPC (%) (95% CI)^b^
**Prevalence (millions)**^c^				

All LMICs	4.51 (3.84–5.29)	12.61 (10.97–14.69)	+179.6	1.85 (1.81–1.90)
GNI-L	0.13 (0.11–0.16)	0.39 (0.32–0.45)	+188.7	0.59 (0.50–0.68)
GNI-LM	1.32 (1.12–1.58)	4.48 (3.88–5.26)	+239.5	2.06 (1.94–2.19)
GNI-UM	3.06 (2.61–3.56)	7.75 (6.77–8.98)	+153.4	2.15 (2.10–2.19)

**Incidence (thousands)**^c^				

All LMICs	294.10 (257.05–339.09)	721.95 (633.06–825.51)	+145.5	1.43 (1.39–1.46)
GNI-L	9.20 (7.95–10.62)	25.78 (22.24–29.79)	+180.4	0.52 (0.44–0.60)
GNI-LM	95.99 (83.91–111.27)	300.69 (264.10–343.97)	+213.3	1.80 (1.70–1.91)
GNI-UM	188.91 (165.19–217.20)	395.47 (346.73–451.76)	+109.3	1.51 (1.46–1.56)

**Deaths (thousands)**^c^				

All LMICs	15.28 (8.38–23.94)	34.09 (18.97–51.91)	+123.2	1.03 (0.95–1.11)
GNI-L	1.32 (0.79–2.00)	2.80 (1.78–4.17)	+111.2	−0.55 (−0.6 to −0.49)
GNI-LM	6.10 (2.41–11.13)	16.84 (7.64–28.50)	+176.2	1.23 (1.15–1.31)
GNI-UM	7.86 (5.19–10.81)	14.46 (9.56–19.25)	+84.1	1.04 (0.92–1.16)

**DALYs (millions)**^c^				

All LMICs	0.99 (0.70–1.33)	2.38 (1.70–3.15)	+141.0	1.34 (1.29–1.39)
GNI-L	0.06 (0.04–0.09)	0.13 (0.09–0.18)	+115.0	−0.49 (−0.59 to −0.39)
GNI-LM	0.31 (0.21–0.45)	0.93 (0.64–1.27)	+198.1	1.57 (1.47–1.66)
GNI-UM	0.61 (0.44–0.80)	1.32 (0.97–1.71)	+114.6	1.61 (1.56–1.65)

Notes: Data are presented as absolute counts with 95% UIs.

Abbreviations: LMICs, low- and middle-income countries; GNI, gross national income; GNI-L, low-income countries; GNI-LM, lower-middle-income countries; GNI-UM, upper-middle-income countries; UI, uncertainty interval; CI, confidence interval.

a PC (%): percentage change in absolute counts on the basis of non-rounded data.

b EAPC: estimated annual percentage change calculated by using all-age rates to capture aggregate trends including demographic shifts.

c Units: millions for prevalence/DALYs and thousands for incidence/deaths.

In 2023, all-age DALY, prevalence, and incidence rates were highest in GNI-UM countries and lowest in GNI-L countries, whereas death rates were similar across income strata. DALY rates were 2.1-fold higher in GNI-UM than in GNI-L (49.5 vs. 23.9 per 100,000), prevalence rates were 4.1-fold higher (290.7 vs. 70.7 per 100,000), and incidence rates were 3.1-fold higher (14.8 vs. 4.7 per 100,000). In contrast, death rates were similar across GNI-L, GNI-LM, and GNI-UM (0.51, 0.58, and 0.54 per 100,000, respectively) (Table 1; Figure S1).

Estimated annual percentage change (EAPC)-based temporal trends further showed that DALY rates declined only in GNI-L, prevalence and incidence rates increased faster in GNI-LM and GNI-UM than in GNI-L, and death rates also declined only in GNI-L. Specifically, DALY rates decreased in GNI-L (EAPC: −0.49%) but increased in GNI-LM and GNI-UM. Prevalence rates increased fastest in GNI-UM (EAPC: 2.15%), whereas incidence rates increased fastest in GNI-LM (EAPC: 1.80%). The corresponding EAPCs were lowest in GNI-L (0.59% and 0.52%, respectively). Death rates decreased in GNI-L (EAPC: −0.55%) but increased in GNI-LM and GNI-UM (Table 1; Figure S1).

### National-level analysis

In 2023, national patterns differed by burden indicator, with age-standardized DALY, prevalence, and incidence rates concentrated in Latin America and age-standardized death rates highest in South Asia and sub-Saharan Africa. DALY rates were highest in Mexico (86.2 per 100,000 population), Peru (76.1), and Honduras (71.5) and lowest in Papua New Guinea (6.7), Kiribati (7.1), and Fiji (7.4), representing a 12.9-fold difference (Figure S2; Table S14). Prevalence and incidence rates followed a similar geographic pattern. The highest prevalence rates were observed in Peru (530.9 per 100,000), Mexico (447.8), and Honduras (371.7), whereas the lowest rates were observed in Papua New Guinea (47.3), Indonesia (47.6), and Chad (50.1), representing an 11.2-fold difference. Incidence rates showed a similar distribution, with the highest rates in Peru (24.9 per 100,000), Mexico (24.9), and Honduras (21.2) and the lowest rates in Papua New Guinea (2.4), Kiribati (2.5), and Fiji (2.6), representing a 10.2-fold difference (Figures S3, S4, and S5; Tables S5 and S8). In contrast, death rates showed a different geographic pattern. The highest death rates were observed in Bangladesh (1.96 per 100,000), Ethiopia (1.83), and Madagascar (1.80), whereas countries with the highest prevalence, such as Peru (0.27) and Mexico (1.27), had lower death rates (Figure S7; Table S11).

**Figure 1:**
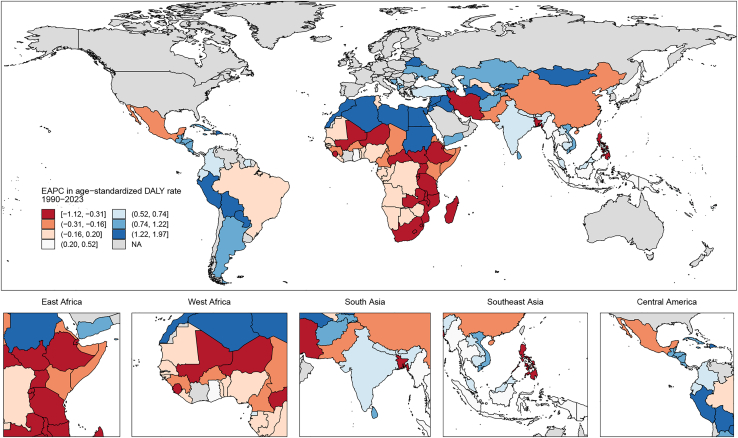
EAPC in age-standardized DALY rates of rheumatoid arthritis in 129 LMICs, 1990–2023

Temporal trends from 1990 to 2023 showed that the largest increases in age-standardized burden rates were mostly concentrated in GNI-UM countries, whereas the largest declines were more heterogeneous across income strata. For DALY rates, the largest increases were observed in GNI-UM countries, including Armenia (EAPC: 2.0%), Mauritius (2.0%), and Peru (1.8%), whereas the most pronounced declines occurred in Rwanda (−1.1%), Mozambique (−0.9%), and South Africa (−0.8%) (Figure 1; Table S15). Prevalence and incidence rate trends showed a similar concentration of the largest increases in GNI-UM countries (Figures S4, S5, and S6; Tables S6 and S9). Death rate trends also showed the largest increases in GNI-UM countries, including Turkmenistan (10.4%), Mauritius (6.9%), and Georgia (6.5%), whereas the largest declines occurred in Iran (−2.7%), Tunisia (−2.4%), and Micronesia (−2.2%) (Figure S8; Table S12). Detailed country-specific data for all burden indicators are provided in Tables S4–S15.

### Sex differences across age groups

Age-specific analysis showed female predominance in RA prevalence and incidence across the three GNI strata, with ratios generally above 1.0 and peak female-to-male ratios shifting toward older ages with increasing GNI level (Figure S9). For prevalence, female-to-male ratios peaked at 20–24 years in GNI-L (2.80), 25–29 years in GNI-LM (3.27), and 40–44 years in GNI-UM (2.58) (Figure S9A). Incidence showed a similar age shift, with peak female-to-male ratios at 15–19 years in GNI-L (3.04), 20–24 years in GNI-LM (3.43), and 30–34 years in GNI-UM (2.72) (Figure S9B). Female-to-male death-rate ratios were more variable than prevalence and incidence ratios, particularly in GNI-L and GNI-LM, where they exceeded 1.0 in most age groups with computable ratios, whereas GNI-UM showed a narrower age-related range (Figure S9C). DALY ratios broadly paralleled the prevalence pattern in GNI-UM but were higher at younger ages in GNI-L and GNI-LM (Figure S9D).

### Inequalities

**Figure 2:**
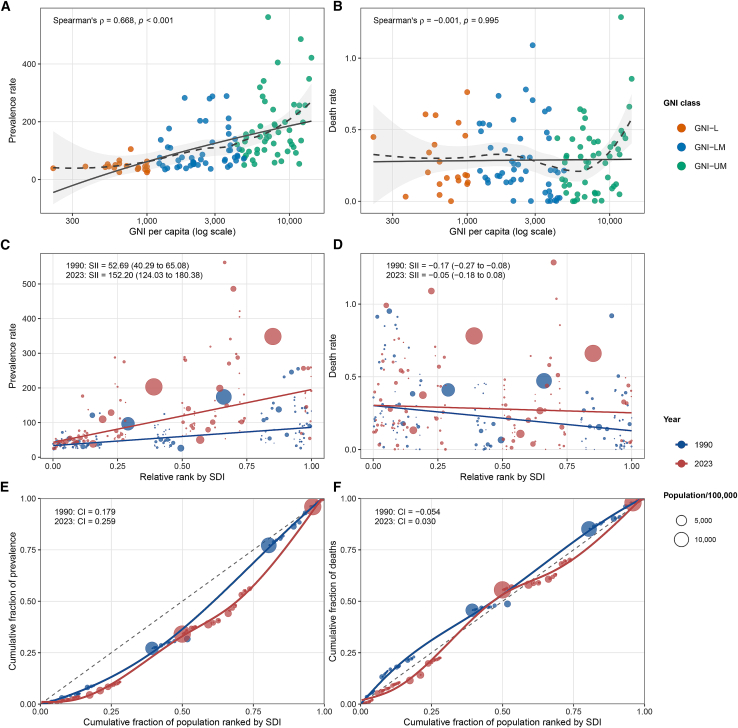
Divergent socioeconomic gradients in prevalence and death burden of rheumatoid arthritis across LMICs, 1990–2023 (A and B) Spearman correlations between GNI per capita and all-age prevalence rates (A) and all-age death rates (B) in 2023. (C and D) Slope index of inequality for all-age prevalence rates (C) and all-age death rates (D) between 1990 and 2023. (E and F) Concentration index for prevalent cases (E) and deaths (F) between 1990 and 2023. *p* values indicate the statistical significance of the Spearman correlations.

At the country level, Spearman correlation analysis showed ecological associations between GNI per capita and all-age RA burden indicators in 2023. The prevalence, incidence, and DALY rates were positively correlated with GNI per capita, whereas death rates showed no correlation. Specifically, prevalence rates (Spearman’s *ρ* = 0.668, *p* < 0.001), incidence rates (*ρ* = 0.583, *p* < 0.001), and DALY rates (*ρ* = 0.389, *p* < 0.001) showed significant positive correlations with national income, while death rates showed no significant correlation (*ρ* = −0.001, *p* = 0.995) (Figures 2A and 2B; Figures S10A and S10B).

Socioeconomic inequality analyses demonstrated different patterns for morbidity-related indicators and death rates between 1990 and 2023. For absolute inequality, SII for prevalence rates increased by 189%, from 52.69 (95% CI: 40.29 to 65.08) in 1990 to 152.20 (95% CI: 124.03 to 180.38) in 2023, indicating widening absolute inequality (Figure 2C). In contrast, SII for death rates moved toward zero, from −0.17 (95% CI: −0.27 to −0.08) to −0.05 (95% CI: −0.18 to 0.08), indicating a narrowing absolute gap in death rates (Figure 2D). Relative inequalities in burden also increased for morbidity-related indicators, with the concentration index rising from 0.179 to 0.259 for prevalent cases, from 0.141 to 0.195 for incident cases, and from 0.092 to 0.178 for DALYs. For deaths, the concentration index shifted from −0.054 in 1990 to 0.030 in 2023, indicating a modest relative shift toward higher-SDI countries (Figures 2E and 2F; Figures S10C–S10F).

### Projections

**Figure 3:**
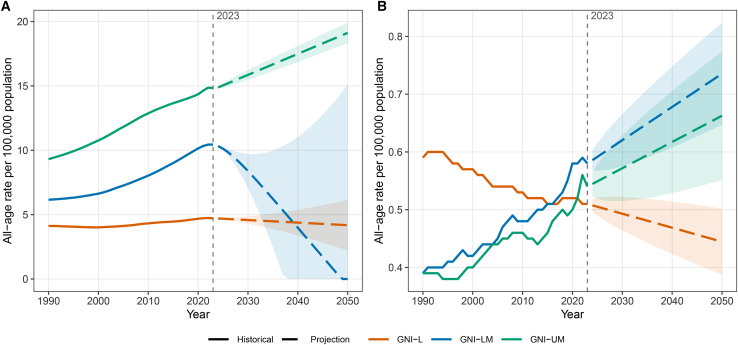
Income-stratified projected trends of RA incidence and death rates in 129 LMICs, 1990–2050 (A) All-age incidence rates and (B) all-age death rates projected from 2024 to 2050 on the basis of 1990–2023 trends. Shaded areas indicate 95% prediction intervals.

Projections to 2050 showed divergent trajectories across GNI strata, characterized by opposite mortality trends and heterogeneous incidence patterns. Death and incidence rates declined in GNI-L, while death rates increased in middle-income countries despite divergent incidence patterns. Specifically, incidence rates declined by 11.4% in GNI-L, followed a downward trajectory in GNI-LM, and rose by 28.8% (from 14.84 to 19.12 per 100,000) in GNI-UM (Figure 3A). Death rates were projected to decline in GNI-L by 12.8% (from 0.51 to 0.44 per 100,000) but increase in GNI-LM by 26.8% (from 0.58 to 0.74) and in GNI-UM by 22.7% (from 0.54 to 0.66) (Figure 3B). Prevalence rates declined in GNI-L by 7.4% but increased in GNI-UM by 23.3%, while DALY rates increased across all GNI strata, with the largest increases in GNI-UM (Figure S11; Table S16).

## Discussion

### Main findings

Using GBD 2023 data, this study quantified RA burdens across 129 LMICs from 1990 to 2023, stratified by GNI level. The main findings are as follows.

(1) All-age death rates rose in GNI-UM but declined in GNI-L, despite GNI-UM having 4.1-fold higher prevalence rates. At the country level, GNI per capita was not correlated with death rates but was positively associated with prevalence and incidence.
(2) Geographic variation was characterized by high death rates despite lower recorded prevalence in several South Asian and sub-Saharan African countries, in contrast to high recorded prevalence but lower death rates in Latin America.
(3) Sex- and age-specific analyses demonstrated higher RA prevalence and incidence among females, with peak female-to-male ratios shifting to older ages in higher-GNI settings.
(4) Absolute inequality in prevalence rates (SII) increased 189% from 1990 to 2023, while inequality in death rates converged toward zero.
(5) Projections to 2050, stratified by income levels, revealed divergent trajectories. Incidence and death rates declined in GNI-L, whereas death rates increased in middle-income countries despite incidence declining in GNI-LM or rising in GNI-UM.

### Income-related divergence

The higher observed DALY, prevalence, and incidence rates in GNI-UM may reflect better case detection and reporting, demographic aging, and risk-factor differences, rather than a simple increase in underlying RA occurrence. Relatively wealthier LMICs may have greater access to rheumatology services and more complete reporting systems. Broader availability of modern diagnostic tools, including magnetic resonance imaging (MRI) and ultrasound, may also improve diagnosis and recording of inflammatory arthritis in better-resourced settings.^21^^,^^22^ The larger increases in prevalence observed in GNI-UM are compatible with this ascertainment-related explanation, although these observations cannot distinguish improved detection from underlying epidemiological change. Demographic aging and risk-factor differences, including smoking and obesity, may also contribute to the recorded burden.^23^ However, the higher recorded morbidity burden in GNI-UM was not accompanied by lower mortality. Instead, death rates increased in GNI-UM while declining in GNI-L.

This discordance should be interpreted in light of the different epidemiological and health-system dimensions captured by each indicator. Incidence is sensitive to case ascertainment, whereas prevalence is additionally shaped by disease duration, survival after diagnosis, and population age structure. Death rates are further influenced by treatment access, comorbidity burden, death certification, and competing mortality risks. In this context, the rising trends in death rates in GNI-UM (EAPC: +1.04%), contrasting with declines in GNI-L (EAPC: −0.55%), may reflect faster expansion of case detection than access to sustained disease-modifying antirheumatic drug (DMARD) therapy, leaving some diagnosed patients without adequate disease control. Research demonstrates substantial disparities in biologic and targeted synthetic DMARD utilization, with rates in regions with lower Human Development Index (HDI) far below those in regions with higher HDI.^24^ In middle-income settings, fragmented healthcare systems, widespread rheumatologist shortages, and limited public reimbursement pose structural barriers to medication access,^25^^,^^26^ compounded by substantial economic burdens, with average annual direct costs per patient ranging from USD 523 to USD 2,837 across LMICs.^27^ These barriers persist despite established treatment guidelines, including those from the American College of Rheumatology^28^ and the WHO essential medicines list,^29^ suggesting fundamental deficits in health system delivery capacity rather than knowledge gaps alone. These treatment-access barriers are particularly evident in Latin America. Studies from Colombia and Peru document that approximately 23% of patients face access barriers associated with supply interruptions and reimbursement approval delays.^30^ Delayed access to effective therapy may contribute to persistent disease activity and comorbidity accumulation, which may partly account for the observed mortality pattern.^6^^,^^7^^,^^31^

### Geographic variation

The substantial geographic variations may reflect differences in case ascertainment and health-system capacity, whereby countries with lower recorded prevalence may nevertheless show higher death rates. Latin American countries, such as Peru, showed high recorded prevalence but low to moderate death rates, a pattern that may be partly related to broader case detection and more established rheumatology-care infrastructure, although treatment-access barriers remain important in this region.^25^^,^^32^ In contrast, regions in South Asia and sub-Saharan Africa, such as Bangladesh, exhibited high death rates despite relatively low reported prevalence. Previous studies have documented limited epidemiological data of RA in LMICs and Africa.^10^^,^^33^ Restricted rheumatology services, shortages of rheumatologists, delayed diagnosis, and barriers to specialist care may further contribute to under-ascertainment of nonfatal RA and preferential capture of more severe or fatal cases.^34^^,^^35^ Together, these factors provide a plausible explanation for the observed low-prevalence/high-mortality pattern.

### Sex- and age-specific patterns

Across GNI strata, females had higher RA prevalence and incidence than males, and peak female-to-male ratios shifted from younger ages in GNI-L and GNI-LM to older ages in GNI-UM. The female excess is consistent with established RA epidemiology and may reflect sex-related immune and hormonal factors.^36^^,^^37^ The later peak in GNI-UM may reflect broader diagnostic access and more complete ascertainment at older ages, whereas earlier peaks in lower-GNI settings may be influenced by delayed diagnosis and differential healthcare access.^35^^,^^38^^,^^39^ Death-rate ratios were more irregular, and the higher DALY ratios at younger ages in GNI-L and GNI-LM may reflect the combined contribution of female-predominant nonfatal burden and premature mortality.

### Socioeconomic inequality

This study found no significant cross-sectional correlation between GNI per capita and death rates in 2023 (Spearman’s *ρ* = −0.001, *p* = 0.995), suggesting that higher national income alone was not associated with lower RA death rates across LMICs. In GNI-UM, prevalence and disability burdens are generally high, yet death rates have not declined proportionally. This pattern aligns with the situation in Latin America, where fragmented medical insurance systems and inadequate prioritization of RA on the health agenda may limit public reimbursement for high-cost therapies, such as biologics, thereby contributing to high out-of-pocket payments.^25^^,^^32^ Absolute health inequalities (SII) widened significantly for prevalence rates, reflecting increasing differences in diagnosed RA burden across the SDI ranking. This widening may reflect greater case ascertainment, population aging, and stronger diagnostic capacity in higher-SDI LMICs, rather than a purely biological increase in RA occurrence.^40^ The parallel concentration-index pattern further suggests that the concentration of prevalence was not limited to the highest- and lowest-SDI countries but also involved countries across the middle of the SDI ranking. Conversely, the convergence of SII for death rates toward zero should not be interpreted as an unequivocal improvement. This pattern may indicate that death rates are becoming similarly elevated across the SDI gradient because mortality is rising in some middle- and higher-SDI settings while lower-SDI settings remain affected by underdiagnosis, incomplete vital registration, and coding limitations.^16^^,^^34^ These patterns suggest that economic development alone does not necessarily translate into equitable RA outcomes unless diagnostic expansion is accompanied by affordable and continuous treatment access.

### Projections and policy implications

Projections to 2050, stratified by income levels, revealed divergent burden trajectories. Incidence and death rates declined in GNI-L, whereas death rates increased in middle-income countries despite incidence declining in GNI-LM or rising in GNI-UM. This divergence suggests that, without effective interventions, gaps between case detection and sustained treatment access may widen, particularly in middle-income settings. The declining incidence in GNI-L may be associated with favorable trends in modifiable risk factors, such as global reductions in smoking rates.^41^^,^^42^ By contrast, the projected rise in incidence in GNI-UM should be interpreted cautiously. It may partly reflect continued expansion of case detection and more complete ascertainment in the underlying data sources,^43^ rather than a purely biological increase in disease occurrence. The trend in GNI-LM is of particular concern. The downward incidence trajectory alongside rising death rates suggests that lower estimated new-case occurrence does not necessarily translate into improved outcomes for patients already living with RA, especially where sustained treatment access and comorbidity management remain limited. Prevalence projections showed a similar divergence. Prevalence rates declined in GNI-L but rose substantially in GNI-UM. This rise may reflect a combination of improved ascertainment, population aging, and longer disease duration, which can increase the number of recorded prevalent cases^17^ even when underlying incidence trends are not increasing to the same extent. In middle-income countries, faster expansion of case detection than access to sustained treatment could contribute to sustained disease activity and accumulated comorbidities (e.g., cardiovascular diseases), which may partly account for the projected increases in mortality.^31^^,^^44^^,^^45^ Targeted health-system interventions are needed, including strengthening diagnostic capacity in GNI-L and prioritizing treatment accessibility in middle-income countries.

The contrast between low recorded prevalence and incidence in GNI-L and rising mortality in GNI-LM and GNI-UM suggests that policy priorities should differ by income setting. In GNI-L, where recorded prevalence and incidence were lowest, policymakers should first strengthen basic case detection and referral capacity, including integration of simple musculoskeletal screening into primary healthcare and clearer referral pathways to specialist or regional services,^38^ because low recorded morbidity burden may partly reflect under-ascertainment rather than genuinely low disease occurrence. In GNI-LM, where incidence followed a downward trajectory but projected death rates rose, policy should focus on retaining diagnosed patients in care, ensuring affordable access to conventional DMARDs and timely treatment escalation where feasible, monitoring treatment response, and managing cardiovascular and other comorbidities that contribute to excess mortality.^25^^,^^26^ In GNI-UM, where DALY, prevalence, and incidence rates were highest and death rates were projected to rise, as well as in high-burden regions such as Latin America, the priority should shift from diagnostic expansion alone to sustained treatment access, including reducing reimbursement delays, expanding insurance coverage for DMARDs and biosimilars, and strengthening the rheumatology workforce.^30^^,^^46^ At the broader LMIC and global-health level, RA should be more explicitly integrated into NCD and health-system monitoring agendas, with standardized indicators for treatment access, DMARD affordability, and rheumatology service capacity to support accountability and resource allocation.^11^^,^^12^ These differentiated strategies are intended to align policy priorities with the observed income-stratified and regional burden patterns and to mitigate future mortality increases.

This research revealed a substantial increase in RA burden across LMICs from 1990 to 2023. The observed patterns are consistent with faster expansion of diagnostic capacity than access to sustained treatment in some middle-income settings. Without effective interventions, the current imbalance between diagnosis and treatment observed in middle-income countries may become a protracted challenge for other emerging economies. Consequently, the future focus of disease control should shift from managing new cases to caring for a large and aging population of prevalent cases. Bridging the gap between diagnostic capability and treatment accessibility and ensuring equitable disease management are critical to curbing the projected rise in death rates by 2050.

### Limitations of the study

This study has several limitations. First, although GBD employs statistical modeling to address data gaps, burden estimates in many LMICs may be biased due to incomplete vital registration systems and insufficient surveillance coverage. Because estimates for data-sparse locations may rely more heavily on statistical borrowing from similar locations, country-level comparisons and rankings should be interpreted cautiously. Second, GNI-based stratification and GNI-burden correlation analyses are ecological analyses based on aggregate country-level estimates. This design carries an inherent risk of ecological fallacy because country-level associations cannot be assumed to represent individual-level effects and cannot capture subnational heterogeneity in RA burden, diagnosis, treatment access, or outcomes. These findings should, therefore, be interpreted as descriptive country-level patterns. Third, because this study did not separately model pandemic-related disruptions, changes during 2020–2023 may partly reflect shifts in healthcare utilization, diagnostic ascertainment, treatment initiation or continuity, and reporting completeness rather than changes in RA epidemiology alone. These period-specific changes should, therefore, be interpreted descriptively rather than as evidence of a direct COVID-19 effect on RA burden. Fourth, ARIMA projections are based on historical trends and do not account for potential future influences from adoption of breakthrough therapies or public health emergencies. Fifth, the proposed explanation involving faster expansion of case detection than access to sustained treatment as well as the interpretation of divergent prevalence, incidence, and death-rate trends is hypothesis-generating because the aggregate GBD estimates do not include individual-level data on age composition, disease duration, treatment exposure, survival after diagnosis, or comorbidity burden. Future real-world studies are needed to validate these explanations.

## Resource availability

### Lead contact

Requests for further information and resources should be directed to and will be fulfilled by the lead contact, Hongnian Wang (hongnianwang@gmail.com).

### Materials availability

This study did not generate new or unique reagents.

### Data and code availability

• This paper analyzes existing, publicly available data from the Global Burden of Disease Study 2023. The estimates used in this study are publicly accessible via the GBD Results tool (https://vizhub.healthdata.org/gbd-results/).
• The code used for data analysis and visualization is publicly available at https://github.com/hongnianwang/ra-lmic-gbd2023.
• Any additional information required to reanalyze the data reported in this paper is available from the lead contact upon request.

## Acknowledgments

We thank the Global Burden of Disease Study 2023 collaborators and the Institute for Health Metrics and Evaluation for making these estimates publicly available. This research was supported by the National Key R&D Program of China-Intergovernmental Key Projects (2023YFE0114300), the Sichuan Science and Technology Program (2026NSFSC1446), the Guangdong Natural Science Foundation General Project (2024A1515012112), the Guangdong Medical Research Fund Project (A2024044), and the Doctoral Research Start-up Fund of North Sichuan Medical College (CBY25-QDA50).

## Author contributions

Conceptualization, H.W.; data curation, H.W., M.Z., and Z.W.; formal analysis, H.W. and M.Z.; funding acquisition, H.W. and L.W.; project administration, L.W.; software, M.Z.; supervision, H.W. and L.W.; visualization, H.W.; writing – original draft, H.W.; writing – review and editing, X.K., Z.W., and L.W. All authors read and approved the final manuscript.

## Declaration of interests

The authors declare no competing interests.

## STAR★Methods

### Key resources table

**Table:** 

REAGENT or RESOURCE	SOURCE	IDENTIFIER
**Deposited data**		

Global Burden of Disease Study 2023 estimates	Institute for Health Metrics and Evaluation (IHME)	https://vizhub.healthdata.org/gbd-results/
GBD 2023 input-source information	Global Health Data Exchange (GHDx)	https://ghdx.healthdata.org/gbd-2023/sources
GBD 2023 Socio-Demographic Index 1950-2023	Global Burden of Disease Collaborative Network/IHME	https://ghdx.healthdata.org/record/gbd-2023-socio-demographic-index-sdi
GNI per capita, Atlas method (current US$), 2023	World Bank World Development Indicators	https://data.worldbank.org/indicator/NY.GNP.PCAP.CD
World Bank country classifications by income level for 2024-2025	World Bank	https://blogs.worldbank.org/en/opendata/world-bank-country-classifications-by-income-level-for-2024-2025

**Software and algorithms**		

R software	R Project for Statistical Computing	https://www.r-project.org/
forecast R package	CRAN	https://cran.r-project.org/package=forecast
MASS R package	CRAN	https://cran.r-project.org/package=MASS

### Experimental model and study participant details

This study analyzed publicly available, aggregate estimates from the Global Burden of Disease (GBD) Study 2023. Analyses were restricted to 129 low- and middle-income countries (LMICs), including all ages and both sexes, from 1990 to 2023. No individual-level data were accessed, no experimental models were used, and institutional ethics approval and informed consent were not required.

### Method details

#### Data source

GBD 2023 provides comprehensive estimates for 204 countries and territories from 1990 to 2023.^15^^,^^16^ For this study, rheumatoid arthritis (RA) estimates were extracted using the GBD Results Tool. Across the overall GBD 2023 framework, input sources included more than 11,000 cause-of-death sources and 35,000 non-fatal sources from vital registration systems, disease registries, and health surveys. GBD 2023 included corrections for misclassified COVID-19 deaths and the redistribution of “garbage codes” to ensure robust estimation across the pandemic period. RA cases were defined according to the 1987 American College of Rheumatology classification criteria. Disease Modeling Meta-Regression 2.1 (DisMod-MR 2.1) and the Cause of Death Ensemble model (CODEm) were used to estimate prevalence and incidence^15^ and death rates,^16^ respectively. For locations with sparse data, hierarchical modeling was applied to borrow information from similar locations. To address methodological differences in input data (e.g., alternative case definitions), the Meta-Regression-Bayesian, Regularised, Trimmed (MR-BRT) tool was used to systematically adjust for known biases. Spatiotemporal Gaussian Process Regression (ST-GPR) was also applied to smooth data trends and borrow strength across time and space. In the GBD 2023 hierarchy, RA is classified as a Level 3 cause under the Level 2 category of musculoskeletal disorders within the Level 1 group of non-communicable diseases. The Socio-demographic Index (SDI) is a composite measure of development used in this study for inequality analysis. It is calculated as the geometric mean of three rescaled components: lag-distributed income per capita, mean educational attainment for individuals aged 15 years and older, and total fertility rate under age 25. SDI values range from 0 to 1. A geographical distribution of the 129 study countries by GBD region is provided in Table S2.

#### Study design

This study examined disparities in RA prevalence rates, incidence rates, death rates, and disability-adjusted life-year (DALY) rates, stratified by national economic status across 129 LMICs (Tables S1 and S2). Countries were classified into three groups according to the World Bank gross national income (GNI) per capita thresholds for fiscal year 2025, which use 2023 calendar-year data^47^: low-income countries (GNI-L; ≤$1,145), lower-middle-income countries (GNI-LM; $1,146-$4,515), and upper-middle-income countries (GNI-UM; $4,516-$14,005). Numeric 2023 GNI per capita values used in correlation analyses were obtained from the World Bank World Development Indicators indicator “GNI per capita, Atlas method (current US$)” (NY.GNP.PCAP.CD). High-income countries were excluded.

GNI was selected as the stratification metric because it provides a standardized World Bank-defined country-level measure of national economic capacity based on Atlas-method GNI per capita.^47^ Similar World Bank income-level or GNI-based stratifications have been used in previous GBD-based public health analyses.^48^^,^^49^ In this study, GNI strata were used as ecological groupings to compare broad income-related patterns across LMICs, including patterns that may relate to national capacity to finance high-cost RA therapies, rather than as proxies for individual socioeconomic status or within-country access to RA care. The study design complied with the Guidelines for Accurate and Transparent Health Estimates Reporting (GATHER)^50^ and GBD 2023 methodological standards.

#### Estimates of rheumatoid arthritis burden

Prevalence rates, incidence rates, death rates, and DALY rates were analyzed. Numbers, all-age rates, and age-standardized rates were extracted or calculated as appropriate. Rates are expressed per 100,000 population. Final GBD estimates were generated from a 250-draw distribution and are presented as means, with 95% uncertainty intervals derived from the 2.5th and 97.5th percentiles of ordered draws.^15^^,^^16^ This approach accounted for uncertainty in sampling and non-sampling variances and model parameter estimation. GBD 2023 reduced the number of draws used to generate final estimates to 250 from 500 in previous cycles to optimize computational efficiency. Simulation testing demonstrated that this reduction minimally affected final mean estimates or uncertainty intervals.^15^^,^^16^

### Quantification and statistical analysis

Age-standardized rates per 100,000 population were calculated using the GBD 2023 standard population.^15^ Age-standardized rates were used for national-level maps and country-level estimated annual percentage change (EAPC) analyses, whereas all-age rates were used for GNI-level temporal trends, Spearman correlation analyses, inequality analyses, and autoregressive integrated moving average (ARIMA) projections to capture population-level burden patterns.

Temporal trends from 1990 to 2023 were quantified using EAPCs derived from log-linear regression models of the form *l*n(*r*) = *α* + *βx*+ε, where *β* is the calendar-year coefficient. EAPC was calculated as 100 × (*e*^*β*^-1). The 95% confidence interval (CI) of EAPC was derived from the standard error of *β* in the fitted regression model. Trends were classified as increasing when the lower bound of the 95% CI was greater than 0, decreasing when the upper bound was less than 0, and stable otherwise. The log-linear model assumes that the log-transformed rate changes approximately linearly with calendar year over the analysis period. Therefore, EAPC was interpreted as an average annual percentage change over 1990–2023, rather than as evidence that each annual series followed a perfectly log-linear trajectory. Year-to-year deviations and short-term fluctuations should be assessed using the annual time-series results and figures, while EAPC summarizes the overall direction and magnitude of temporal change.

For analyses requiring GNI per capita as a continuous variable, countries without available numeric 2023 Atlas-method GNI per capita values in the World Bank World Development Indicators were excluded from the correlation analysis. Spearman’s rank correlation coefficient *ρ* was calculated to examine ecological associations between all-age burden indicators and GNI per capita among countries with available numeric GNI data (*n* = 123):

$$\rho =1-\frac{6\sum d_{i}^{2}}{n\left(n^{2}-1\right)}$$

where *d*_*i*_ is the difference between the ranks of corresponding values of GNI per capita and burden indicator for country *i*, and *n* is the number of countries included in the correlation analysis. GNI was used for income-group stratification and Spearman correlation analyses, whereas SDI was used for inequality ranking.

Health inequalities were evaluated at the country level across the 129 LMICs using annual all-age rates and SDI ranking. SDI values were obtained from the GBD 2023 SDI dataset and restricted to national-level GBD locations matching the study countries. Countries were ranked from lower to higher SDI, and population midpoint ranks were calculated from cumulative population shares. The slope index of inequality (SII) was estimated by robust regression of all-age rates on the population midpoint rank ordered by SDI. Concentration curves were generated after ranking countries from lower to higher SDI, and the concentration index was calculated using burden numbers, including prevalent cases, incident cases, deaths, and DALYs.

The SII was estimated as the slope coefficient from a robust linear regression of all-age rates on the population midpoint rank:

$$y_{i}=\beta_{0}+\beta_{1}\times R_{i}+\varepsilon_{i}$$

where *β*_1_ (SII) represents the absolute difference in burden between the highest (1) and lowest (0) ends of the SDI spectrum.

The concentration index was calculated from the concentration curve as:

CI=1-2AUC

where AUC represents the area under the concentration curve relating the cumulative fraction of burden to the cumulative fraction of population ranked from lower to higher SDI.

Female-to-male ratios were calculated by age group and GNI stratum to describe sex-specific patterns of RA burden. Non-seasonal ARIMA models were used to project all-age burden rates to 2050. The general form of a non-seasonal ARIMA (p, d, q) model is:

$$\Phi \left(B\right){\left(1-B\right)}^{d}y_{t}=\Theta \left(B\right)\varepsilon_{t}$$

where *y*_*t*_ is the burden metric at time *t*, where *p* is the autoregressive order, *d* is the degree of differencing, and *q* is the moving average order. In this backshift notation, *Φ*(*B*) is the autoregressive polynomial operator of order *p*, and *Θ*(*B*) is the moving average polynomial operator of order *q*. The Akaike information criterion (AIC) serves as a standard for model selection, finding the optimal balance between goodness-of-fit and model parsimony. It is calculated as:

AIC=−2ln(L)+2k

where *L* is the maximized likelihood of the model and *k* is the number of estimated parameters (p + q, plus the variance, etc.).

Model orders were selected using the auto.arima function through an information-criterion-based search. Model fit was summarized by AIC and Bayesian information criterion (BIC). Residual autocorrelation was assessed using the Ljung-Box test at lag 10. Forecast uncertainty was presented using 95% prediction intervals. These intervals reflected ARIMA forecasting uncertainty based on point-estimate time series and did not propagate GBD draw-level uncertainty. Because ARIMA forecasts were not constrained to be non-negative, long-horizon projections crossing below zero were interpreted as indicating continued downward trajectories rather than valid negative disease rates. Model parameters and diagnostics are provided in Table S3. Analyses were conducted using R version 4.3.3. A two-sided *p* value < 0.05 was considered statistically significant.
