# Supplementary material for: Rheumatoid arthritis burden in 129 low- and middle-income countries from 1990 to 2023
Source: iScience. 2026 Jul 21;29(8):116881. doi: 10.1016/j.isci.2026.116881 (PMC13392949; doi:10.1016/j.isci.2026.116881)
Supplement: Document S1. Figures S1–S11 and Tables S1–S16 [file mmc1.pdf]

iScience, Volume *29*

Supplemental information

Rheumatoid arthritis burden in 129 low- and middle-income countries from 1990 to 2023

Hongnian Wang, Mingyang Zhang, Xiong Ke, Zichao Wang, and Lijuan Wu

Figure S1. Trends in all-age rates (per 100,000) of rheumatoid arthritis burden by GNI stratum, 1990–2023

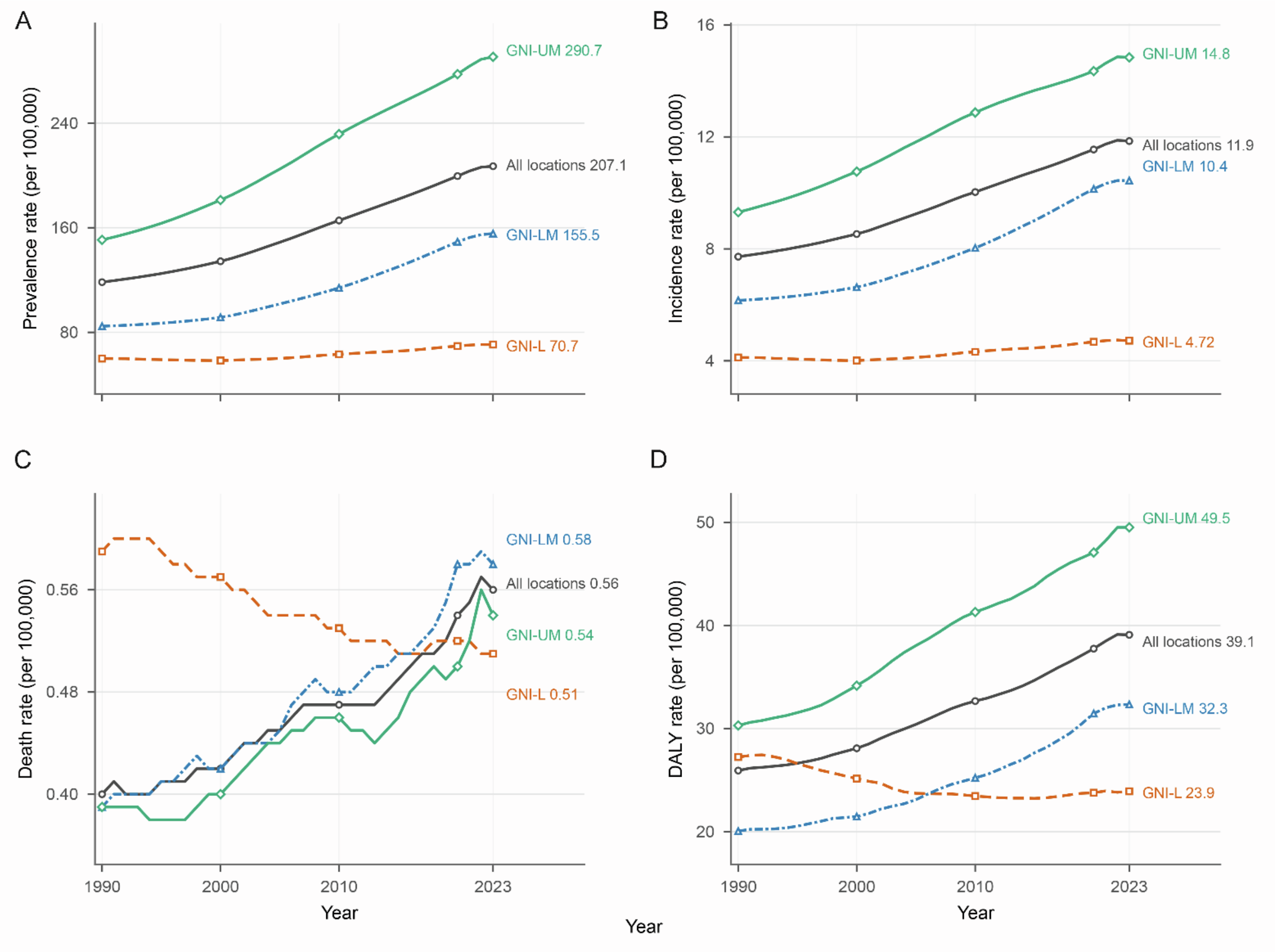

(A) All-age prevalence rates, (B) all-age incidence rates, (C) all-age death rates, and (D) all-age DALY rates across GNI-L, GNI-LM, and GNI-UM countries.

Figure S2. Age-standardized DALY rates (per 100,000) of rheumatoid arthritis in 129 LMICs, 2023

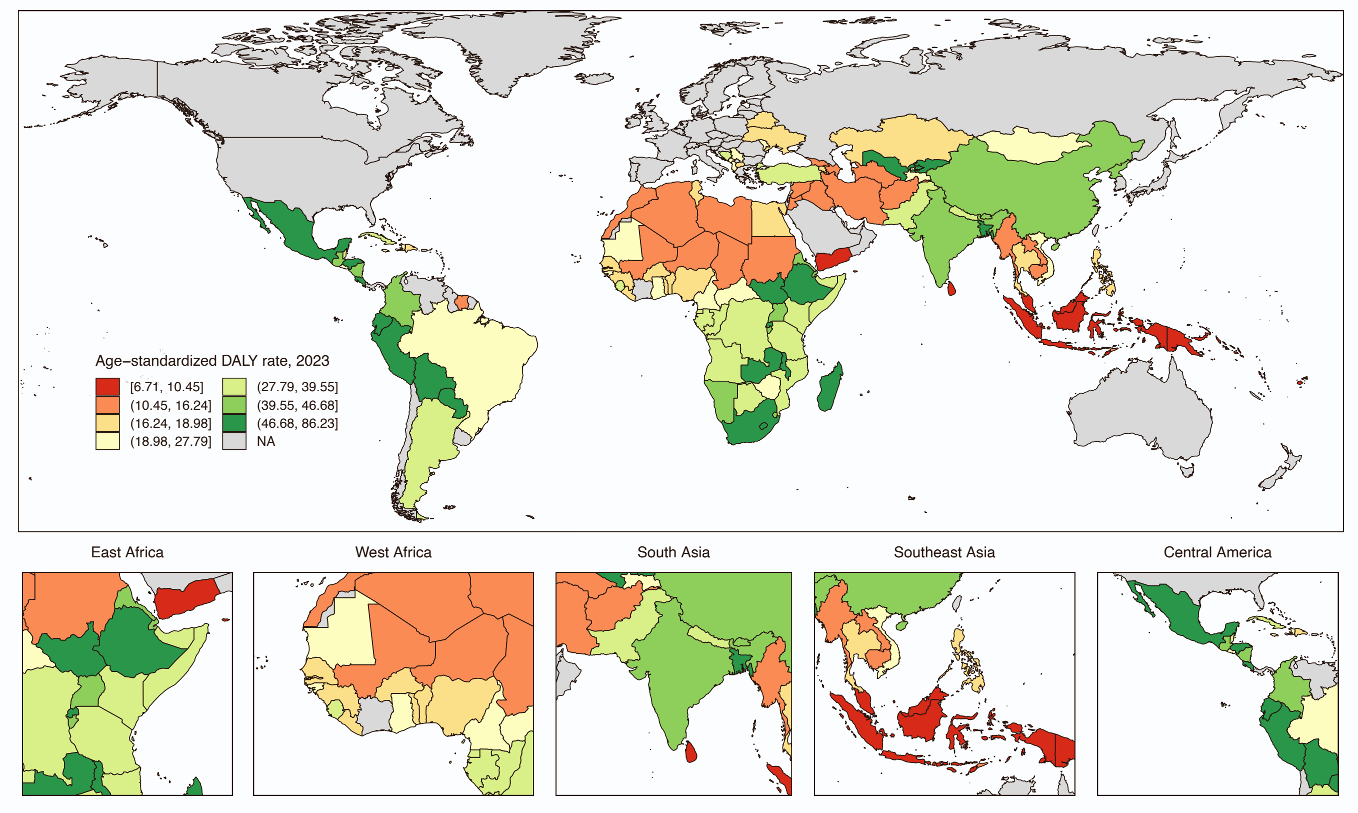

Figure S3. Age-standardized prevalence rates (per 100,000) of rheumatoid arthritis in 129 LMICs, 2023

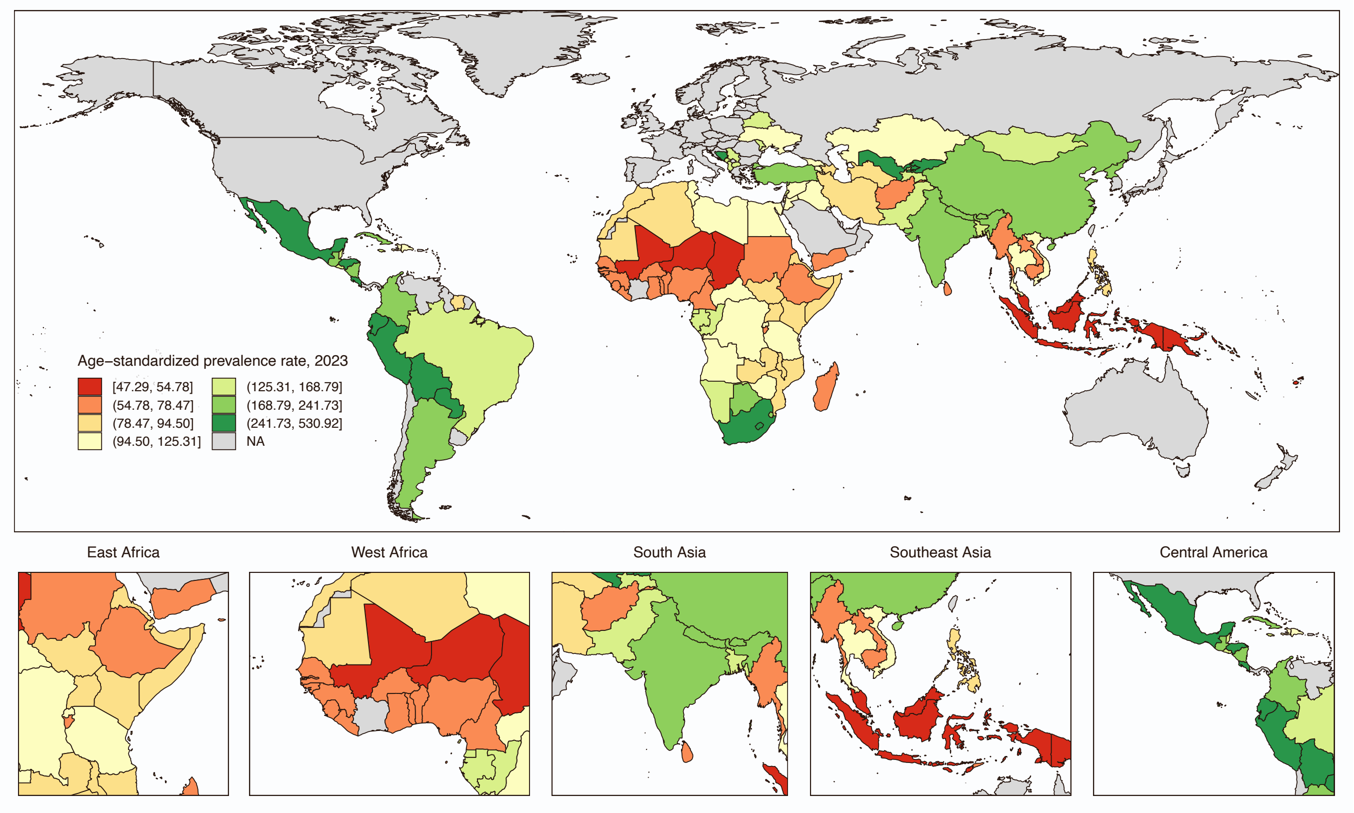

Figure S4. EAPC in age-standardized prevalence rates of rheumatoid arthritis in 129 LMICs, 1990–2023

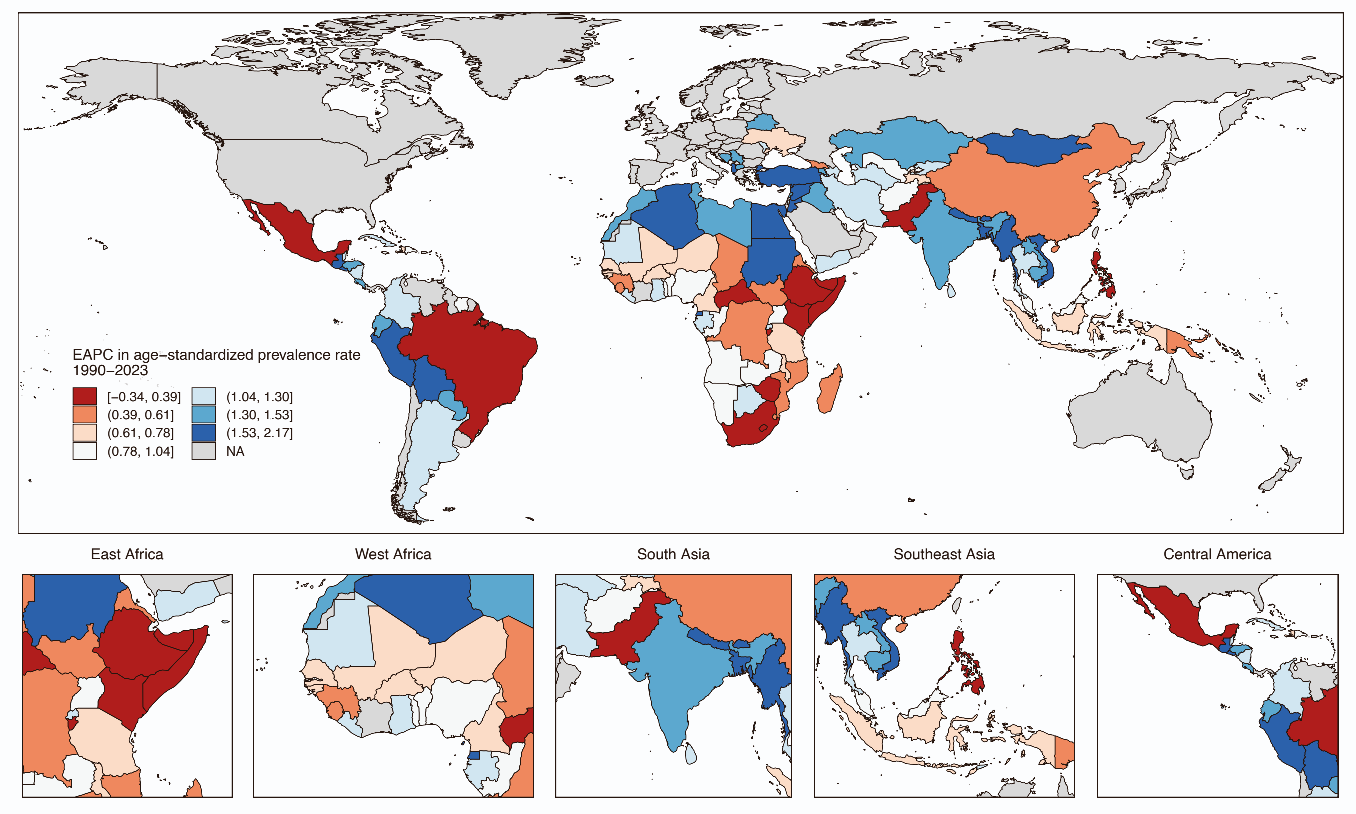

Figure S5. Age-standardized incidence rates (per 100,000) of rheumatoid arthritis in 129 LMICs, 2023

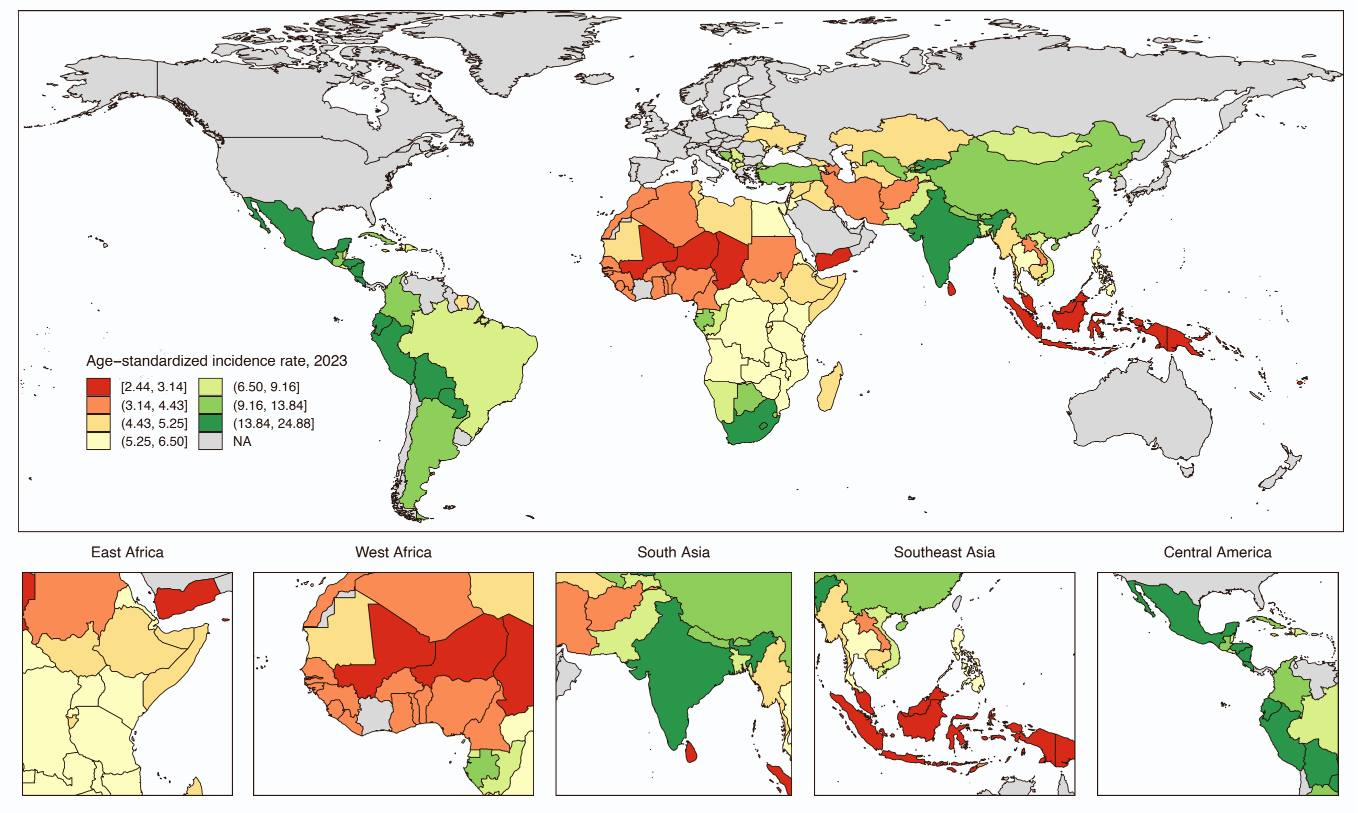

Figure S6. EAPC in age-standardized incidence rates of rheumatoid arthritis in 129 LMICs, 1990–2023

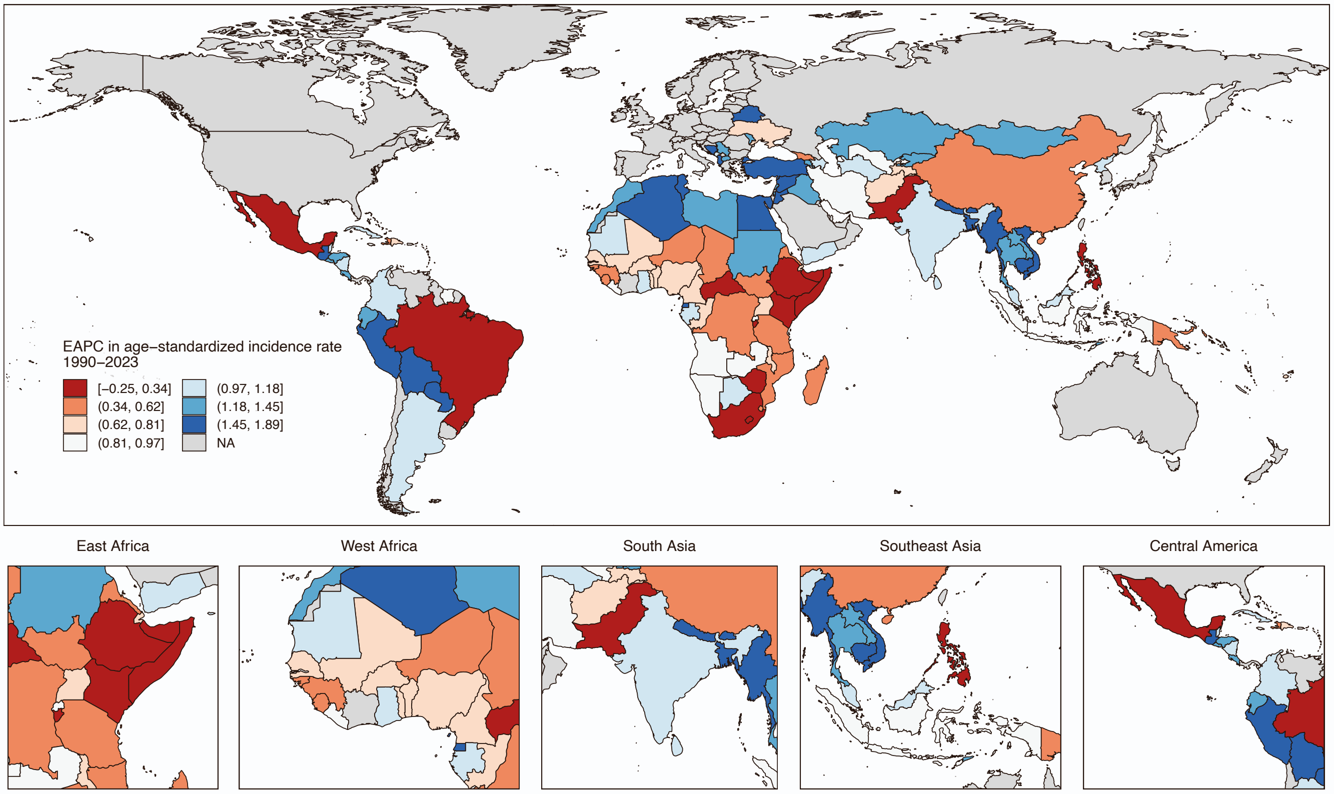

Figure S7. Age-standardized death rates (per 100,000) of rheumatoid arthritis in 129 LMICs, 2023

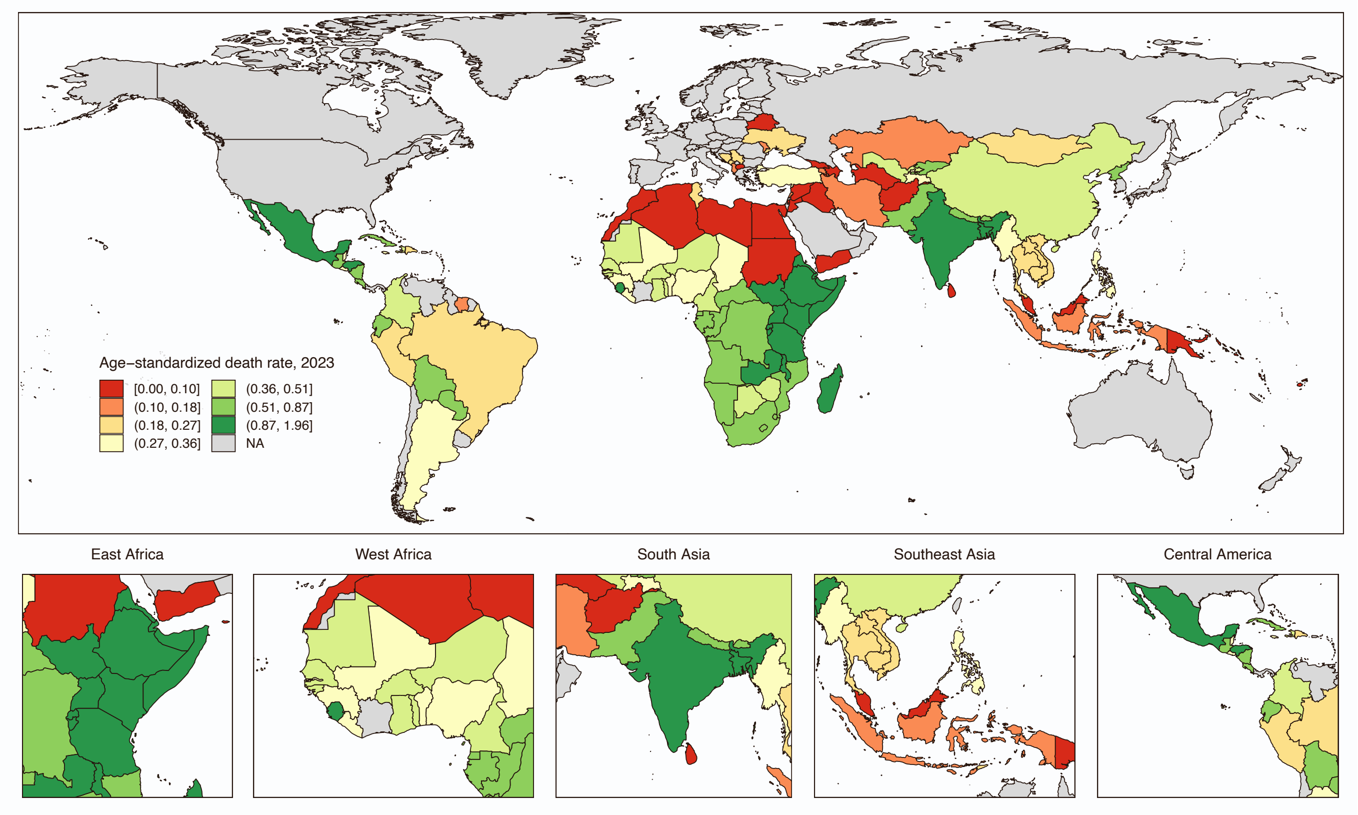

Figure S8. EAPC in age-standardized death rates of rheumatoid arthritis in 129 LMICs, 1990–2023

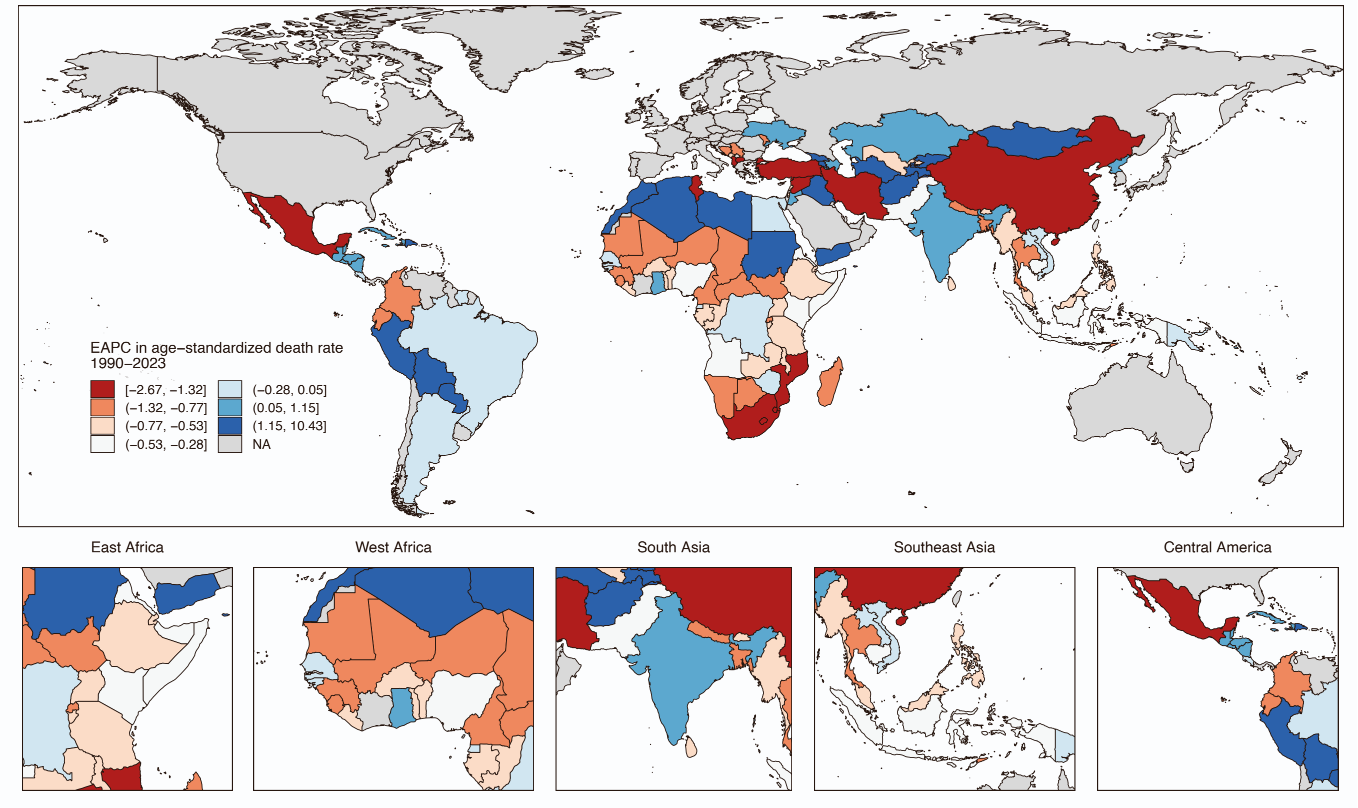

Figure S9. Female-to-male ratios of rheumatoid arthritis burden by age and GNI stratum, 2023

(A) Prevalence, (B) incidence, (C) deaths, and (D) DALYs

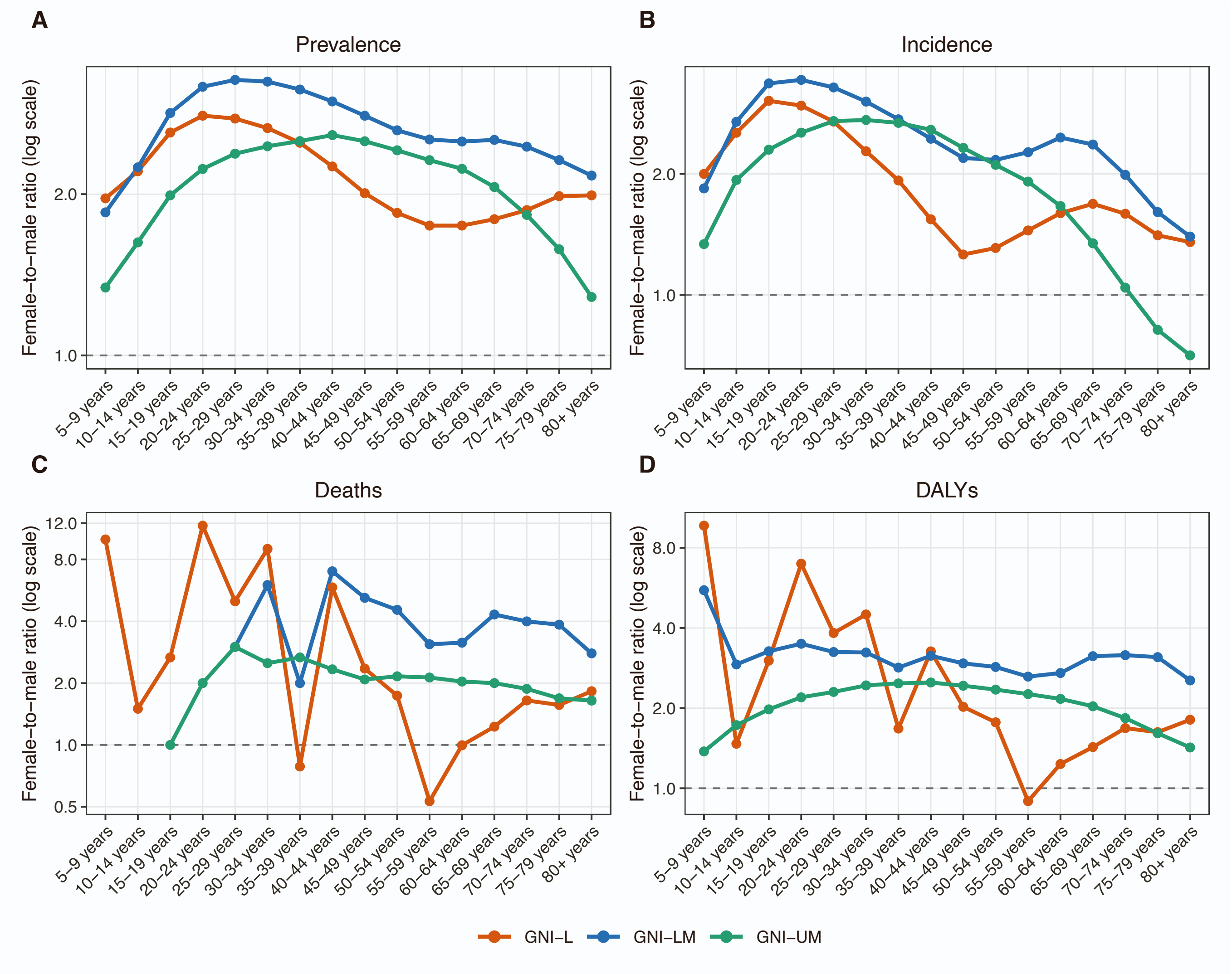

Figure S10. Socioeconomic gradients in all-age rheumatoid arthritis incidence and DALY burden across LMICs, 1990–2023

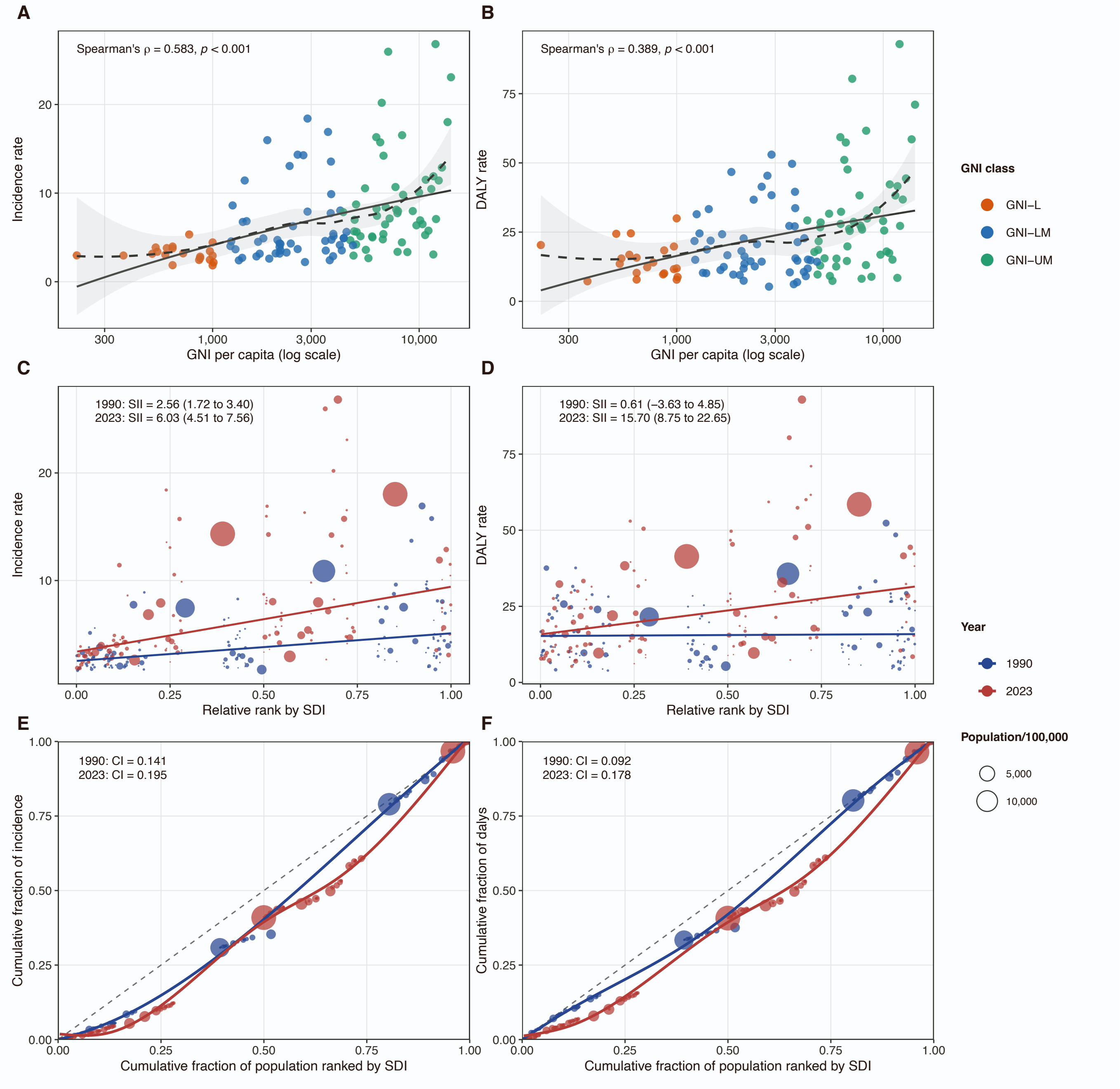

(A, B) Spearman correlations between GNI per capita and all-age incidence rates (A) and all-age DALY rates (B) in 2023. (C, D) Slope index of inequality for all-age incidence rates (C) and all-age DALY rates (D), between 1990 and 2023. (E, F) Concentration index for incident cases (E) and DALYs (F), between 1990 and 2023.

Figure S11. Income-stratified projected trends of RA prevalence and DALY rates in 129 LMICs, 1990-2050

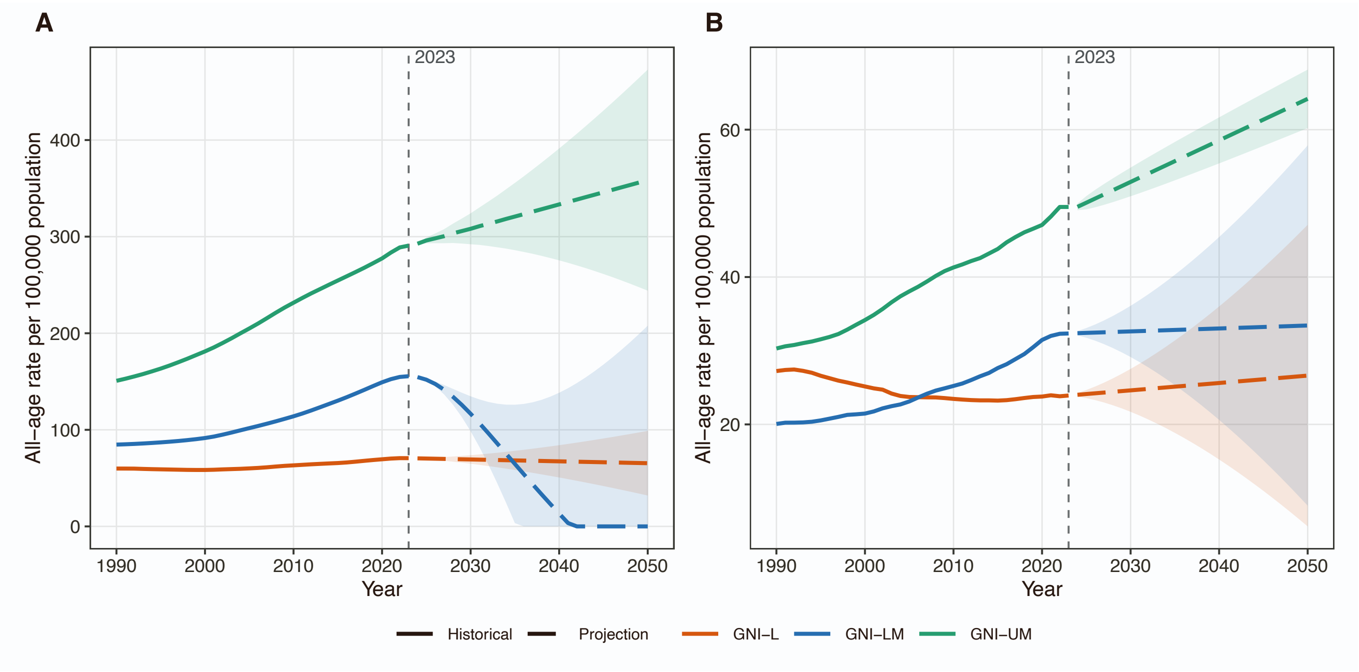

(A) All-age prevalence rates and (B) all-age DALY rates projected from 2024 to 2050 based on 1990-2023 trends.

Table S1. GNI Classification of 129 low- and middle-income countries

Location	GNI Classification	Location	GNI Classification
Afghanistan	L	Madagascar	L
Albania	UM	Malawi	L
Algeria	UM	Malaysia	UM
Angola	LM	Maldives	UM
Argentina	UM	Mali	L
Armenia	UM	Marshall Islands	UM
Azerbaijan	UM	Mauritania	LM
Bangladesh	LM	Mauritius	UM
Belarus	UM	Mexico	UM
Belize	UM	Micronesia	LM
Benin	LM	Moldova	UM
Bhutan	LM	Mongolia	UM
Bolivia	LM	Montenegro	UM
Bosnia and Herzegovina	UM	Morocco	LM
Botswana	UM	Mozambique	L
Brazil	UM	Myanmar	LM
Burkina Faso	L	Namibia	UM
Burundi	L	Nepal	LM
Cambodia	LM	Nicaragua	LM
Cameroon	LM	Niger	L
Cape Verde	LM	Nigeria	LM
Central African Republic	L	North Korea	L
Chad	L	North Macedonia	UM
China	UM	Pakistan	LM
Colombia	UM	Palestine	LM
Comoros	LM	Papua New Guinea	LM
Congo (Brazzaville)	LM	Paraguay	UM
Costa Rica	UM	Peru	UM
Cuba	UM	Philippines	LM
Democratic Republic of Congo	L	Rwanda	L
Djibouti	LM	Saint Lucia	UM
Dominica	UM	Saint Vincent and the Grenadines	UM
Dominican Republic	UM	Samoa	LM
Ecuador	UM	Sao Tome and Principe	LM
Egypt	LM	Senegal	LM
El Salvador	UM	Serbia	UM
Equatorial Guinea	UM	Sierra Leone	L

Eritrea	L	Solomon Islands	LM
Ethiopia	L	Somalia	L
Fiji	UM	South Africa	UM
Gabon	UM	South Sudan	L
Gambia	L	Sri Lanka	LM
Georgia	UM	Sudan	L
Ghana	LM	Suriname	UM
Grenada	UM	Swaziland	LM
Guatemala	UM	Syria	L
Guinea	LM	Tajikistan	LM
Guinea-Bissau	L	Tanzania	LM
Haiti	LM	Thailand	UM
Honduras	LM	Timor-Leste	LM
India	LM	Togo	L
Indonesia	UM	Tonga	UM
Iran	UM	Tunisia	LM
Iraq	UM	Turkiye	UM
Jamaica	UM	Turkmenistan	UM
Jordan	LM	Tuvalu	UM
Kazakhstan	UM	Uganda	L
Kenya	LM	Ukraine	UM
Kiribati	LM	Uzbekistan	LM
Kyrgyzstan	LM	Vanuatu	LM
Laos	LM	Vietnam	LM
Lebanon	LM	Yemen	L
Lesotho	LM	Zambia	LM
Liberia	L	Zimbabwe	LM
Libya	UM		

Note: Classification based on World Bank fiscal year 2025 definitions, using 2023 GNI per capita. L (Low income): ≤$1,145; LM (Lower-middle income): $1,146–$4,515; UM (Upper-middle income): $4,516–$14,005 GNI per capita.

Table S2. Geographical distribution of the 129 study locations by GBD regional hierarchy

GBD Super-Region	GBD Region	Countries included in this study
**Central Europe, Eastern Europe, and Central Asia**	Central Asia	Armenia, Azerbaijan, Georgia, Kazakhstan, Kyrgyzstan, Mongolia, Tajikistan, Turkmenistan, Uzbekistan
	Central Europe	Albania, Bosnia and Herzegovina, Montenegro, North Macedonia, Serbia
	Eastern Europe	Belarus, Moldova, Ukraine
**Latin America and Caribbean**	Andean Latin America	Bolivia, Ecuador, Peru
	Caribbean	Belize, Cuba, Dominica, Dominican Republic, Grenada, Haiti, Jamaica, Saint Lucia, Saint Vincent and the Grenadines, Suriname
	Central Latin America	Colombia, Costa Rica, El Salvador, Guatemala, Honduras, Mexico, Nicaragua
	Southern Latin America	Argentina
	Tropical Latin America	Brazil, Paraguay
**North Africa and Middle East**	North Africa and Middle East	Afghanistan, Algeria, Egypt, Iran, Iraq, Jordan, Lebanon, Libya, Morocco, Palestine, Sudan, Syria, Tunisia, Turkiye, Yemen
**South Asia**	South Asia	Bangladesh, Bhutan, India, Nepal, Pakistan, Sri Lanka
**Southeast Asia, East Asia, and Oceania**	East Asia	China, North Korea
	Oceania	Fiji, Kiribati, Marshall Islands, Micronesia, Papua New Guinea, Samoa, Solomon Islands, Tonga, Tuvalu, Vanuatu
	Southeast Asia	Cambodia, Indonesia, Laos, Malaysia, Maldives, Myanmar, Philippines, Thailand, Timor-Leste, Vietnam
**Sub-Saharan Africa**	Central Sub-Saharan Africa	Angola, Central African Republic, Congo (Brazzaville), Democratic Republic of Congo, Equatorial Guinea, Gabon, Sao Tome and Principe
	Eastern Sub-Saharan Africa	Burundi, Comoros, Djibouti, Eritrea, Ethiopia, Kenya, Madagascar, Malawi, Mauritius, Mozambique, Rwanda, Somalia, South Sudan, Tanzania, Uganda, Zambia
	Southern Sub-Saharan Africa	Botswana, Lesotho, Namibia, South Africa, Swaziland, Zimbabwe
	Western Sub-Saharan Africa	Benin, Burkina Faso, Cameroon, Cape Verde, Chad, Gambia, Ghana, Guinea, Guinea-Bissau, Liberia, Mali, Mauritania, Niger, Nigeria, Senegal, Sierra Leone, Togo

Note: This table lists only the 129 low- and middle-income countries (LMICs) included in the present analysis, grouped according to the standard Global Burden of Disease (GBD) geographical hierarchy (available at http://ghdx.healthdata.org/gbd-2023).

Table S3. ARIMA model parameters and diagnostics for burden indicators by GNI stratum (1990–2023) using all-age rates

Measures	Location	ARIMA model	AIC	BIC	p-value
**DALYs**					
	GNI-L	ARIMA(0,2,0)	-40.15	-38.69	0.8836
	GNI-LM	ARIMA(0,2,0)	-28.7	-27.23	0.6409
	GNI-UM	ARIMA(0,1,1)	2.74	7.23	0.8439
**Deaths**					
	GNI-L	ARIMA(0,1,0)	-245.49	-242.5	0.3238
	GNI-LM	ARIMA(0,1,0)	-216.72	-213.72	0.5328
	GNI-UM	ARIMA(0,1,0)	-201.48	-198.49	0.4832
**Incidence**					
	GNI-L	ARIMA(0,2,0)	-190.31	-188.84	0.7983
	GNI-LM	ARIMA(1,2,2)	-164.3	-158.43	0.7592
	GNI-UM	ARIMA(0,1,1)	-111.2	-106.71	0.9978
**Prevalence**					
	GNI-L	ARIMA(0,2,1)	-46.88	-43.95	0.8715
	GNI-LM	ARIMA(2,2,0)	-27.86	-23.47	0.9769
	GNI-UM	ARIMA(2,2,2)	54.97	62.3	0.9999

Notes: Model parameters were automatically selected using the `auto.arima` function in the R forecast package. AIC and BIC were used to summarize model fit and parsimony. The p value refers to the Ljung-Box test at lag 10 for residual autocorrelation. Abbreviations: LMICs, low- and middle-income countries; GNI-L, low-income countries; GNI-LM, lower-middle-income countries; GNI-UM, upper-middle-income countries; ARIMA, autoregressive integrated moving average.

Table S4. Rheumatoid arthritis counts and age-standardized prevalence rates in 129 LMICs, 1990

Location	Prevalent cases (95% UI, 1990)	Age-standardized prevalence rate (per 100,000, 95% UI, 1990)
Afghanistan	4113.49 (3425.56-4837.77)	54.79 (46.11-64.72)
Albania	2533.78 (2150.23-3038.17)	94.50 (79.49-111.47)
Algeria	8563.62 (7031.47-10159.30)	51.09 (42.92-60.69)
Angola	4666.20 (3938.99-5445.49)	84.83 (74.42-96.86)
Argentina	47560.39 (41206.09-55482.55)	147.41 (127.73-172.17)
Armenia	2386.33 (1976.95-2859.64)	75.70 (63.09-90.53)
Azerbaijan	4091.83 (3370.50-4987.21)	66.67 (55.38-80.78)
Bangladesh	57837.11 (48211.70-68579.23)	87.16 (74.00-102.30)
Belarus	10147.32 (8485.36-11974.98)	82.95 (69.20-98.25)
Belize	124.81 (105.79-146.94)	110.70 (96.57-128.23)
Benin	1351.57 (1086.77-1673.63)	50.79 (41.80-61.27)
Bhutan	464.74 (384.66-561.05)	132.67 (114.04-154.92)
Bolivia	7979.77 (6782.67-9234.24)	191.89 (167.87-218.01)
Bosnia and Herzegovina	7814.70 (6826.81-8873.94)	168.78 (148.65-190.56)
Botswana	1073.54 (895.09-1253.61)	137.91 (117.90-158.52)
Brazil	206083.99 (169587.69-245656.37)	167.24 (138.97-199.04)
Burkina Faso	2645.56 (2148.08-3215.47)	48.17 (40.20-57.14)
Burundi	2127.31 (1781.73-2498.76)	72.01 (63.02-82.36)
Cabo Verde	121.11 (99.74-146.76)	53.04 (43.69-64.28)
Cambodia	2932.90 (2422.07-3531.29)	47.68 (40.22-56.61)
Cameroon	3689.70 (2996.05-4505.42)	59.81 (49.58-72.45)
Central African Republic	1464.12 (1254.50-1699.41)	93.28 (81.98-106.61)
Chad	1526.17 (1223.61-1865.17)	43.56 (35.86-52.94)
China	2052563.84 (1755864.10-2383923.50)	205.75 (179.74-238.28)
Colombia	36362.73 (30978.12-42517.29)	159.46 (138.88-182.55)
Comoros	204.88 (173.86-242.85)	77.61 (66.92-90.20)
Congo (Brazzaville)	1567.79 (1340.71-1802.56)	115.39 (101.40-129.95)
Costa Rica	4514.93 (3904.04-5216.58)	219.61 (193.32-249.65)
Cuba	15453.89 (13248.35-17901.72)	143.66 (122.90-166.45)
DR Congo	17391.69 (14689.73-20322.01)	83.54 (72.91-95.05)
Democratic Republic of Sao Tome and Principe	41.70 (34.06-50.85)	55.92 (46.36-67.17)
Djibouti	145.91 (119.30-176.11)	72.00 (62.50-83.66)
Dominica	70.58 (61.22-81.26)	107.11 (93.09-124.09)
Dominican Republic	3602.74 (2987.07-4344.42)	74.98 (63.27-88.35)
Ecuador	13155.01 (11503.33-15076.01)	199.91 (177.55-226.97)

Egypt	28031.89 (22616.33-33592.48)	67.35 (55.43-79.78)
El Salvador	3436.82 (2898.71-4036.91)	95.68 (81.03-110.74)
Equatorial Guinea	213.52 (179.68-245.37)	86.72 (74.70-98.22)
Eritrea	1286.00 (1087.26-1498.85)	72.03 (62.65-81.80)
Ethiopia	22377.18 (18742.28-26644.89)	81.32 (69.88-95.78)
Fiji	244.68 (191.55-301.75)	41.58 (33.02-50.47)
Gabon	733.65 (629.97-841.31)	116.20 (100.86-132.25)
Gambia	267.00 (213.91-333.59)	49.75 (40.89-60.39)
Georgia	4981.07 (4092.90-6071.13)	82.29 (67.77-100.01)
Ghana	4195.69 (3357.89-5247.72)	46.92 (38.23-57.08)
Grenada	53.85 (45.55-64.27)	70.10 (58.62-83.18)
Guatemala	5053.25 (4194.77-5860.75)	108.64 (93.72-125.59)
Guinea	1896.39 (1539.77-2308.31)	47.90 (39.60-57.89)
Guinea-Bissau	276.50 (224.53-340.04)	47.74 (39.69-57.83)
Haiti	3334.23 (2822.59-3901.68)	78.74 (68.51-90.23)
Honduras	5980.03 (5267.67-6860.85)	232.37 (209.87-262.19)
India	817452.77 (702426.00-977215.50)	149.21 (131.05-174.05)
Indonesia	48816.67 (39203.83-60876.72)	36.17 (29.58-44.30)
Iran	23723.23 (19353.61-28571.48)	63.24 (52.36-75.14)
Iraq	8901.97 (7130.76-11045.64)	74.33 (62.34-90.38)
Jamaica	1704.72 (1447.66-1991.23)	91.85 (78.15-107.22)
Jordan	1683.41 (1360.24-2058.95)	71.20 (58.93-85.62)
Kazakhstan	11439.93 (9432.68-13997.01)	75.79 (62.84-91.91)
Kenya	8729.25 (7253.73-10513.54)	79.95 (69.07-94.17)
Kiribati	23.09 (18.62-28.37)	41.52 (34.32-50.31)
Kyrgyzstan	7391.96 (6424.98-8534.75)	226.53 (198.43-256.23)
Laos	1300.68 (1068.75-1577.96)	48.12 (40.55-56.68)
Lebanon	3054.79 (2523.95-3606.73)	111.67 (93.96-130.86)
Lesotho	1406.66 (1198.64-1631.33)	142.18 (122.99-162.87)
Liberia	825.39 (683.66-1021.20)	51.95 (43.61-62.66)
Libya	1897.26 (1549.04-2292.17)	66.20 (55.36-79.45)
Madagascar	4089.57 (3410.31-4852.73)	63.28 (54.07-73.60)
Malawi	3889.57 (3244.99-4571.68)	76.64 (66.35-87.96)
Malaysia	4681.93 (3812.98-5848.45)	37.32 (30.67-45.93)
Maldives	96.89 (82.07-114.73)	75.50 (64.83-88.15)
Mali	2319.96 (1889.97-2862.91)	44.22 (36.84-52.86)
Marshall Islands	13.17 (10.65-15.90)	50.72 (42.52-59.38)
Mauritania	710.12 (577.86-857.75)	55.79 (46.48-66.53)
Mauritius	564.21 (472.70-686.07)	62.96 (53.62-75.96)

Mexico	209498.10 (182586.28-240153.35)	400.65 (352.74-452.36)
Micronesia	37.92 (30.65-45.88)	56.77 (47.28-66.66)
Moldova	4026.01 (3341.19-4788.54)	86.87 (71.95-103.01)
Mongolia	1344.45 (1127.62-1582.29)	96.46 (82.02-111.98)
Montenegro	831.46 (702.89-999.81)	130.45 (111.00-156.28)
Morocco	11400.07 (9198.65-13790.67)	59.07 (48.74-70.79)
Mozambique	5603.31 (4780.30-6585.29)	71.39 (62.65-82.61)
Myanmar	14599.66 (12074.86-17615.86)	49.09 (41.32-57.86)
Namibia	1108.19 (933.45-1301.44)	128.56 (110.65-148.80)
Nepal	13966.10 (11644.13-16396.50)	115.14 (99.43-132.06)
Nicaragua	3321.16 (2812.75-3805.36)	164.44 (143.35-185.76)
Niger	1807.32 (1447.02-2279.56)	42.71 (35.19-52.51)
Nigeria	27489.00 (22282.20-34276.75)	47.98 (39.96-58.71)
North Korea	29486.20 (25354.29-34072.43)	158.37 (137.82-180.64)
North Macedonia	1832.88 (1498.93-2202.64)	89.84 (73.80-107.62)
Pakistan	134955.52 (114082.62-161321.86)	183.95 (159.57-217.79)
Palestine	920.70 (729.04-1118.98)	75.48 (61.57-89.63)
Papua New Guinea	1114.09 (891.15-1396.13)	39.15 (32.18-47.76)
Paraguay	5141.25 (4289.63-6145.39)	181.54 (154.15-214.03)
Peru	50400.42 (43204.81-57552.48)	310.28 (272.10-351.77)
Philippines	40947.37 (34083.80-49179.33)	99.36 (85.53-117.45)
Rwanda	2984.81 (2534.54-3489.12)	78.93 (68.22-90.14)
Saint Lucia	110.54 (95.32-128.53)	112.12 (96.98-128.70)
Saint Vincent and the Grenadines	60.19 (50.85-71.89)	75.14 (63.04-88.94)
Samoa	71.12 (57.99-86.24)	61.37 (50.90-72.98)
Senegal	2225.94 (1785.94-2750.75)	51.72 (42.82-61.96)
Serbia	12111.89 (11164.59-13264.34)	110.14 (101.64-119.85)
Sierra Leone	1299.18 (1056.60-1596.83)	48.22 (40.35-58.06)
Solomon Islands	106.13 (86.16-126.50)	49.70 (41.54-58.11)
Somalia	3074.96 (2578.78-3652.06)	78.40 (67.96-89.90)
South Africa	95128.18 (82790.16-109443.27)	346.60 (303.26-394.20)
South Sudan	2091.68 (1727.47-2488.43)	68.62 (59.20-80.08)
Sri Lanka	5696.11 (4648.48-7137.08)	40.48 (33.13-49.93)
Sudan	6688.72 (5411.19-8111.89)	49.68 (41.25-59.28)
Suriname	205.76 (168.96-247.01)	66.23 (55.20-78.98)
Swaziland	762.82 (637.61-883.63)	175.33 (149.74-200.37)
Syria	5258.76 (4253.89-6408.68)	66.23 (55.20-79.66)
Tajikistan	3987.70 (3397.98-4603.94)	115.76 (99.71-132.51)
Tanzania	10724.77 (9014.46-12655.84)	76.97 (66.95-88.72)

Thailand	30987.51 (25850.43-36654.06)	67.19 (57.39-78.18)
Timor-Leste	210.05 (168.92-258.58)	45.27 (38.11-53.74)
Togo	955.07 (759.16-1195.04)	50.57 (41.71-60.54)
Tonga	40.03 (32.67-47.94)	57.54 (47.38-68.44)
Tunisia	4264.02 (3474.06-5148.91)	65.25 (54.17-77.76)
Turkiye	61549.71 (54456.82-69831.29)	137.93 (122.77-155.73)
Turkmenistan	1677.31 (1375.62-2029.56)	65.39 (53.89-78.41)
Tuvalu	3.95 (3.24-4.72)	49.20 (40.72-58.24)
Uganda	6088.16 (5078.33-7277.45)	72.44 (62.82-84.29)
Ukraine	48333.17 (40159.75-57863.37)	74.70 (62.06-89.76)
Uzbekistan	32594.77 (28016.03-36939.87)	238.64 (210.00-266.47)
Vanuatu	47.53 (37.95-57.58)	47.47 (39.04-56.35)
Vietnam	34918.97 (29075.72-41423.67)	73.55 (61.67-86.46)
Yemen	3629.88 (2936.08-4369.37)	46.11 (38.29-54.89)
Zambia	2948.99 (2452.36-3454.92)	74.48 (64.63-85.42)
Zimbabwe	5997.50 (4940.90-7115.77)	101.01 (85.54-117.50)

Abbreviations: LMICs: Low- and Middle-Income Countries; UI: Uncertainty Interval.

Table S5. Rheumatoid arthritis counts and age-standardized prevalence rates in 129 LMICs, 2023

Location	Prevalent cases (95% UI, 2023)	Age-standardized prevalence rate (per 100,000, 95% UI, 2023)
Afghanistan	16394.32 (13528.20-19414.60)	72.36 (61.21-84.16)
Albania	5872.60 (5009.05-6867.32)	161.79 (137.32-192.08)
Algeria	37330.27 (30874.87-43825.99)	81.88 (68.35-95.92)
Angola	21007.01 (17842.36-24485.33)	107.68 (94.64-122.16)
Argentina	121976.19 (107716.00-140074.42)	220.36 (193.53-253.82)
Armenia	4828.99 (4090.64-5547.48)	117.73 (98.21-136.52)
Azerbaijan	10787.65 (9022.24-13041.91)	88.14 (73.55-106.31)
Bangladesh	223931.60 (189421.27-260997.03)	141.86 (121.64-164.64)
Belarus	18178.20 (15509.95-21259.05)	130.56 (110.32-154.43)
Belize	588.14 (498.85-689.34)	158.17 (136.24-183.87)
Benin	5564.21 (4483.69-6799.13)	65.82 (54.81-78.49)
Bhutan	1580.69 (1366.82-1825.15)	216.25 (189.58-248.35)
Bolivia	34853.57 (30160.60-40317.63)	314.17 (276.37-358.61)
Bosnia and Herzegovina	12930.91 (11572.75-14470.50)	254.86 (226.54-285.41)
Botswana	3997.47 (3349.81-4619.76)	186.47 (159.83-211.70)
Brazil	421736.87 (351629.06-494877.00)	168.79 (139.92-198.59)
Burkina Faso	8662.64 (7025.90-10554.34)	60.39 (50.44-71.47)
Burundi	5570.27 (4634.45-6646.32)	74.61 (64.52-86.70)
Cabo Verde	355.87 (289.47-424.59)	74.41 (61.50-87.25)
Cambodia	11922.67 (10124.64-14231.46)	75.66 (64.94-89.59)
Cameroon	15639.14 (12621.71-18985.21)	74.22 (61.99-88.13)
Central African Republic	3493.74 (3007.79-4034.00)	94.94 (83.36-107.35)
Chad	4791.38 (3840.24-5880.01)	50.10 (41.52-60.23)
China	4985071.17 (4389030.62-5800939.69)	241.73 (211.74-280.44)
Colombia	140933.68 (123584.48-160355.59)	240.50 (210.14-274.30)
Comoros	586.72 (499.98-688.42)	90.80 (78.69-105.22)
Congo (Brazzaville)	5289.98 (4454.18-6137.25)	139.13 (121.93-157.13)
Costa Rica	21952.13 (19823.19-24951.92)	358.58 (323.14-407.41)
Cuba	33719.93 (29211.43-38023.19)	204.23 (176.28-235.63)
DR Congo	51867.84 (43278.38-60746.28)	95.57 (82.26-109.59)
Democratic Republic of Sao Tome and Principe	126.31 (102.09-152.85)	75.34 (63.23-89.48)
Djibouti	897.72 (752.88-1067.96)	90.41 (78.02-104.65)
Dominica	118.24 (102.67-134.32)	134.43 (115.53-153.61)
Dominican Republic	10926.43 (9248.19-12931.81)	101.56 (86.60-119.20)
Ecuador	60587.93 (53077.47-68135.31)	333.45 (292.27-374.80)

Egypt	111244.45 (92892.63-131974.05)	115.16 (97.47-134.67)
El Salvador	9692.90 (8263.16-11383.78)	154.15 (131.41-180.79)
Equatorial Guinea	1227.11 (1005.94-1447.37)	149.90 (129.35-170.44)
Eritrea	3737.66 (3178.94-4406.01)	83.90 (73.49-96.55)
Ethiopia	51076.62 (42946.28-60923.37)	72.86 (62.70-85.53)
Fiji	501.42 (407.44-609.65)	53.74 (43.71-65.05)
Gabon	2387.95 (2043.19-2755.25)	164.43 (142.76-187.27)
Gambia	1028.69 (823.25-1251.13)	65.67 (54.33-78.26)
Georgia	4715.09 (4037.92-5471.99)	99.88 (84.09-117.41)
Ghana	16827.66 (13674.07-20592.43)	69.62 (57.91-84.82)
Grenada	151.19 (129.02-173.32)	108.08 (92.10-124.38)
Guatemala	27088.45 (23827.79-31195.75)	203.23 (180.28-231.75)
Guinea	4940.04 (3996.99-6051.04)	57.99 (48.71-70.18)
Guinea-Bissau	761.56 (615.84-931.78)	58.55 (48.89-69.10)
Haiti	10358.97 (8801.42-12054.22)	98.77 (85.56-112.96)
Honduras	30847.73 (27639.61-34267.20)	371.70 (335.90-411.22)
India	2931088.26 (2569177.49-3425759.58)	218.18 (191.92-252.29)
Indonesia	144914.87 (119591.01-177691.02)	47.62 (39.65-57.54)
Iran	89724.87 (74254.42-107175.46)	90.16 (74.73-107.15)
Iraq	40171.31 (32919.24-48252.61)	107.61 (90.67-126.50)
Jamaica	4219.34 (3640.50-4886.19)	133.07 (114.39-153.96)
Jordan	13877.46 (11588.91-16583.51)	111.54 (93.64-131.41)
Kazakhstan	22908.06 (18764.06-27143.78)	106.93 (87.97-127.15)
Kenya	31261.87 (26419.80-37051.77)	91.57 (79.38-106.40)
Kiribati	55.55 (45.33-67.11)	50.37 (41.53-60.37)
Kyrgyzstan	20492.57 (18131.46-23356.30)	327.93 (292.48-369.74)
Laos	4604.08 (3830.18-5498.37)	72.93 (62.04-85.71)
Lebanon	10324.33 (8611.72-12169.53)	168.08 (140.49-197.03)
Lesotho	2637.45 (2256.19-3038.20)	173.36 (149.70-196.28)
Liberia	2717.30 (2181.72-3328.15)	67.58 (55.98-80.69)
Libya	7677.32 (6257.31-9074.47)	97.09 (80.48-113.70)
Madagascar	13197.18 (10920.69-15580.55)	74.09 (63.75-85.29)
Malawi	10485.32 (8791.78-12421.34)	93.65 (81.16-106.98)
Malaysia	18564.13 (15467.78-22356.71)	51.66 (43.29-61.84)
Maldives	501.18 (413.56-598.14)	98.17 (83.23-114.50)
Mali	7541.57 (6046.60-9332.57)	54.78 (45.48-65.23)
Marshall Islands	24.40 (19.95-28.90)	67.66 (56.60-79.29)
Mauritania	2435.11 (1962.52-2919.94)	81.32 (67.25-94.92)
Mauritius	1720.32 (1476.42-2009.87)	98.39 (83.62-116.31)

Mexico	641185.30 (562148.53-716154.37)	447.81 (394.38-500.13)
Micronesia	79.10 (64.24-92.70)	81.60 (67.23-94.92)
Moldova	6662.16 (5691.75-7897.75)	126.34 (107.30-150.59)
Mongolia	4554.09 (3877.82-5281.47)	141.85 (122.58-162.59)
Montenegro	1523.76 (1320.00-1765.25)	165.37 (141.02-192.19)
Morocco	37323.26 (31731.07-44392.95)	92.59 (78.71-110.19)
Mozambique	15026.91 (12888.61-17667.45)	87.95 (76.96-101.21)
Myanmar	43647.13 (36562.65-51483.83)	78.14 (66.01-91.24)
Namibia	3660.18 (3054.56-4271.52)	163.95 (141.80-187.76)
Nepal	54832.51 (46622.37-63950.68)	192.03 (165.87-223.03)
Nicaragua	13850.85 (12073.89-16017.54)	236.55 (207.63-269.61)
Niger	6774.35 (5450.04-8350.50)	51.37 (42.61-60.87)
Nigeria	94705.06 (76808.73-116136.58)	63.19 (53.09-76.17)
North Korea	72743.51 (64033.39-81040.40)	213.13 (188.96-237.46)
North Macedonia	3479.85 (2945.37-4174.04)	130.59 (107.92-156.05)
Pakistan	267388.97 (227611.96-321097.13)	146.72 (125.09-174.88)
Palestine	4508.38 (3652.84-5464.62)	107.50 (89.50-126.94)
Papua New Guinea	4088.35 (3325.01-4943.07)	47.29 (39.35-56.55)
Paraguay	20179.48 (17403.72-23248.01)	321.14 (279.09-367.97)
Peru	203082.56 (177101.96-231656.03)	530.92 (464.21-604.95)
Philippines	91052.77 (77263.13-109595.12)	89.68 (76.94-106.99)
Rwanda	8727.34 (7315.16-10217.81)	95.96 (83.42-109.80)
Saint Lucia	354.02 (306.83-405.46)	151.75 (130.32-175.48)
Saint Vincent and the Grenadines	141.14 (121.03-166.03)	99.38 (84.32-118.04)
Samoa	143.37 (118.21-168.62)	78.45 (64.98-92.55)
Senegal	7853.63 (6321.55-9540.34)	66.29 (54.89-79.49)
Serbia	23259.04 (20475.40-26483.07)	167.06 (144.08-193.35)
Sierra Leone	3108.44 (2506.61-3879.16)	57.83 (47.79-69.31)
Solomon Islands	454.72 (376.42-542.87)	67.54 (57.55-78.66)
Somalia	8908.09 (7583.71-10396.52)	78.89 (69.49-90.58)
South Africa	183707.00 (158973.60-211768.64)	284.97 (248.24-325.93)
South Sudan	4783.94 (3960.24-5752.01)	80.43 (68.20-93.78)
Sri Lanka	15652.87 (13041.77-19042.48)	57.40 (47.48-70.14)
Sudan	24859.11 (20398.83-29586.29)	78.47 (65.67-93.02)
Suriname	574.63 (477.48-683.54)	87.86 (73.42-103.97)
Swaziland	1874.69 (1570.05-2168.94)	202.24 (173.19-230.78)
Syria	18739.41 (15391.27-22569.72)	106.81 (88.53-129.09)
Tajikistan	11965.93 (9993.34-13989.69)	145.66 (125.00-167.29)
Tanzania	36426.53 (30350.76-43120.55)	95.45 (82.80-109.91)

Thailand	98017.47 (84519.38-113621.26)	97.35 (83.02-114.46)
Timor-Leste	690.75 (576.48-825.76)	65.92 (55.71-77.98)
Togo	3833.52 (3100.30-4679.60)	64.04 (53.27-75.99)
Tonga	69.87 (57.44-84.06)	76.66 (63.47-91.75)
Tunisia	14221.92 (11838.00-17119.48)	102.01 (84.35-123.52)
Turkiye	220385.24 (197727.42-247443.66)	217.86 (195.80-244.44)
Turkmenistan	4823.06 (4054.17-5734.94)	90.83 (76.75-107.34)
Tuvalu	6.98 (5.78-8.27)	67.85 (56.80-79.92)
Uganda	20832.56 (17524.50-24851.26)	91.70 (79.76-105.39)
Ukraine	61869.92 (52093.21-72843.00)	98.22 (81.93-117.41)
Uzbekistan	98992.91 (85435.36-113041.79)	286.98 (250.67-322.78)
Vanuatu	163.36 (133.40-194.80)	61.59 (51.36-73.01)
Vietnam	144586.75 (123751.48-168923.70)	125.31 (107.71-146.43)
Yemen	15944.75 (12948.73-19119.49)	62.14 (51.76-73.38)
Zambia	10036.68 (8377.37-11939.06)	94.50 (81.35-109.29)
Zimbabwe	11960.39 (10116.23-14093.57)	104.81 (89.34-121.20)

Abbreviations: LMICs: Low- and Middle-Income Countries; UI: Uncertainty Interval.

Table S6. Percentage change in prevalent cases and EAPC in age-standardized prevalence rates of rheumatoid arthritis in 129 LMICs, 1990–2023

Location	Prevalence PC (%, 1990–2023)	Prevalence EAPC (%, 95% CI, 1990–2023)
Afghanistan	299	0.96 (0.82 to 1.10)
Albania	132	1.83 (1.76 to 1.90)
Algeria	336	1.58 (1.49 to 1.67)
Angola	350	0.96 (0.88 to 1.05)
Argentina	156	1.15 (1.05 to 1.25)
Armenia	102	1.49 (1.44 to 1.54)
Azerbaijan	164	1.18 (0.92 to 1.44)
Bangladesh	287	1.79 (1.61 to 1.96)
Belarus	79	1.49 (1.36 to 1.61)
Belize	371	1.14 (1.09 to 1.19)
Benin	312	0.88 (0.80 to 0.96)
Bhutan	240	1.68 (1.57 to 1.80)
Bolivia	337	1.60 (1.58 to 1.63)
Bosnia and Herzegovina	65	1.51 (1.41 to 1.61)
Botswana	272	1.09 (0.95 to 1.22)
Brazil	105	0.13 (0.08 to 0.18)
Burkina Faso	227	0.78 (0.71 to 0.84)
Burundi	162	0.29 (0.23 to 0.35)
Cabo Verde	194	1.17 (1.08 to 1.27)
Cambodia	307	1.47 (1.43 to 1.50)
Cameroon	324	0.74 (0.58 to 0.91)
Central African Republic	139	0.17 (0.13 to 0.21)
Chad	214	0.49 (0.37 to 0.62)
China	143	0.58 (0.55 to 0.61)
Colombia	288	1.30 (1.16 to 1.45)
Comoros	186	0.63 (0.55 to 0.71)
Congo (Brazzaville)	237	0.83 (0.75 to 0.92)
Costa Rica	386	1.43 (1.35 to 1.52)
Cuba	118	1.10 (1.03 to 1.17)
DR Congo	198	0.50 (0.40 to 0.61)
Democratic Republic of Sao Tome and Principe	203	1.02 (0.90 to 1.13)
Djibouti	515	0.84 (0.73 to 0.96)
Dominica	68	0.63 (0.57 to 0.69)
Dominican Republic	203	0.92 (0.87 to 0.97)
Ecuador	361	1.52 (1.43 to 1.60)

Egypt	297	1.55 (1.47 to 1.63)
El Salvador	182	1.53 (1.42 to 1.64)
Equatorial Guinea	475	2.17 (1.97 to 2.37)
Eritrea	191	0.51 (0.49 to 0.53)
Ethiopia	128	-0.09 (-0.17 to -0.01)
Fiji	105	0.80 (0.67 to 0.93)
Gabon	225	1.23 (1.17 to 1.29)
Gambia	285	0.95 (0.86 to 1.03)
Georgia	-5	0.58 (0.50 to 0.67)
Ghana	301	1.26 (1.15 to 1.37)
Grenada	181	1.13 (1.04 to 1.21)
Guatemala	436	1.79 (1.67 to 1.91)
Guinea	160	0.61 (0.51 to 0.72)
Guinea-Bissau	175	0.68 (0.58 to 0.79)
Haiti	211	0.72 (0.66 to 0.77)
Honduras	416	1.37 (1.30 to 1.44)
India	259	1.34 (1.24 to 1.44)
Indonesia	197	0.78 (0.73 to 0.83)
Iran	278	1.11 (1.09 to 1.13)
Iraq	351	1.36 (1.20 to 1.52)
Jamaica	148	1.04 (0.95 to 1.12)
Jordan	724	1.66 (1.56 to 1.75)
Kazakhstan	100	1.34 (1.11 to 1.57)
Kenya	258	0.39 (0.30 to 0.48)
Kiribati	141	0.53 (0.47 to 0.60)
Kyrgyzstan	177	1.30 (1.18 to 1.42)
Laos	254	1.38 (1.31 to 1.45)
Lebanon	238	1.42 (1.31 to 1.53)
Lesotho	87	0.47 (0.35 to 0.58)
Liberia	229	1.06 (0.92 to 1.21)
Libya	305	1.32 (1.24 to 1.39)
Madagascar	223	0.55 (0.51 to 0.59)
Malawi	170	0.75 (0.68 to 0.82)
Malaysia	297	1.04 (1.00 to 1.08)
Maldives	417	1.10 (0.76 to 1.44)
Mali	225	0.75 (0.67 to 0.83)
Marshall Islands	85	0.89 (0.83 to 0.95)
Mauritania	243	1.20 (1.11 to 1.30)
Mauritius	205	1.22 (1.16 to 1.28)

Mexico	206	0.30 (0.21 to 0.39)
Micronesia	109	1.08 (1.03 to 1.13)
Moldova	65	1.27 (1.12 to 1.42)
Mongolia	239	1.56 (1.42 to 1.70)
Montenegro	83	1.00 (0.85 to 1.14)
Morocco	227	1.45 (1.35 to 1.54)
Mozambique	168	0.61 (0.56 to 0.67)
Myanmar	199	1.54 (1.45 to 1.62)
Namibia	230	0.88 (0.77 to 0.98)
Nepal	293	1.70 (1.63 to 1.77)
Nicaragua	317	1.29 (1.22 to 1.35)
Niger	275	0.67 (0.58 to 0.75)
Nigeria	245	0.94 (0.79 to 1.09)
North Korea	147	0.96 (0.92 to 1.00)
North Macedonia	90	1.31 (1.24 to 1.39)
Pakistan	98	-0.04 (-0.24 to 0.15)
Palestine	390	1.09 (1.00 to 1.18)
Papua New Guinea	267	0.51 (0.44 to 0.58)
Paraguay	293	1.53 (1.42 to 1.63)
Peru	303	1.87 (1.78 to 1.96)
Philippines	122	-0.22 (-0.27 to -0.17)
Rwanda	192	1.05 (0.91 to 1.19)
Saint Lucia	220	0.90 (0.76 to 1.04)
Saint Vincent and the Grenadines	134	0.90 (0.87 to 0.94)
Samoa	102	0.67 (0.62 to 0.72)
Senegal	253	0.76 (0.68 to 0.85)
Serbia	92	1.39 (1.26 to 1.53)
Sierra Leone	139	0.57 (0.46 to 0.68)
Solomon Islands	328	0.87 (0.83 to 0.92)
Somalia	190	0.10 (0.08 to 0.13)
South Africa	93	-0.34 (-0.42 to -0.26)
South Sudan	129	0.59 (0.54 to 0.64)
Sri Lanka	175	1.17 (1.07 to 1.28)
Sudan	272	1.53 (1.40 to 1.66)
Suriname	179	0.96 (0.88 to 1.03)
Swaziland	146	0.46 (0.31 to 0.61)
Syria	256	1.58 (1.48 to 1.67)
Tajikistan	200	0.69 (0.64 to 0.74)
Tanzania	240	0.69 (0.63 to 0.76)

Thailand	216	1.30 (1.20 to 1.39)
Timor-Leste	229	1.19 (1.16 to 1.23)
Togo	301	0.79 (0.66 to 0.92)
Tonga	75	0.77 (0.71 to 0.83)
Tunisia	234	1.50 (1.40 to 1.60)
Turkiye	258	1.63 (1.56 to 1.70)
Turkmenistan	188	1.18 (1.05 to 1.32)
Tuvalu	77	0.94 (0.91 to 0.97)
Uganda	242	0.90 (0.80 to 1.00)
Ukraine	28	0.76 (0.72 to 0.79)
Uzbekistan	204	0.82 (0.74 to 0.91)
Vanuatu	244	0.78 (0.74 to 0.82)
Vietnam	314	1.63 (1.59 to 1.68)
Yemen	339	1.05 (0.97 to 1.13)
Zambia	240	0.92 (0.78 to 1.07)
Zimbabwe	99	-0.17 (-0.33 to -0.01)

Notes: PC was calculated from all-age counts between 1990 and 2023, whereas EAPC was calculated from age-standardized rates over the 1990-2023 time series. Abbreviations: LMICs: Low- and Middle-Income Countries; PC: Percentage Change; EAPC: Estimated Annual Percentage Change; CI: Confidence Interval.

Table S7. Rheumatoid arthritis incident cases and age-standardized incidence rates in 129 LMICs, 1990

Location	Incidence cases (95% UI, 1990)	Age-standardized incidence rate (per 100,000, 95% UI, 1990)
Afghanistan	232.11 (196.20-270.96)	2.76 (2.37-3.18)
Albania	152.72 (130.02-179.85)	5.06 (4.34-5.92)
Algeria	543.05 (452.67-640.63)	2.56 (2.19-2.98)
Angola	347.01 (302.72-396.64)	5.30 (4.80-5.93)
Argentina	2673.71 (2376.75-2996.55)	8.27 (7.35-9.27)
Armenia	128.17 (108.67-154.83)	3.79 (3.22-4.53)
Azerbaijan	232.61 (194.42-279.74)	3.32 (2.80-3.97)
Bangladesh	4130.97 (3559.61-4860.62)	5.18 (4.58-5.97)
Belarus	463.25 (394.47-546.30)	4.10 (3.45-4.81)
Belize	8.75 (7.56-10.08)	6.53 (5.85-7.36)
Benin	94.40 (78.77-113.12)	2.96 (2.56-3.44)
Bhutan	34.51 (29.61-40.47)	8.42 (7.48-9.44)
Bolivia	529.46 (464.00-607.70)	11.08 (10.06-12.32)
Bosnia and Herzegovina	419.56 (371.41-475.57)	8.64 (7.64-9.70)
Botswana	73.22 (62.43-84.00)	7.73 (6.91-8.68)
Brazil	11232.21 (9398.65-13130.31)	7.53 (6.48-8.72)
Burkina Faso	181.74 (154.83-214.68)	2.85 (2.47-3.33)
Burundi	156.66 (135.98-180.66)	4.61 (4.14-5.25)
Cabo Verde	7.58 (6.43-8.91)	2.95 (2.51-3.48)
Cambodia	202.51 (173.42-236.46)	2.78 (2.43-3.19)
Cameroon	252.59 (210.82-300.80)	3.42 (2.95-3.97)
Central African Republic	104.64 (93.31-117.66)	5.75 (5.28-6.39)
Chad	105.04 (88.34-124.76)	2.58 (2.20-3.03)
China	128307.83 (112655.41-147427.10)	11.59 (10.23-13.22)
Colombia	2432.68 (2123.14-2775.63)	9.03 (8.17-10.12)
Comoros	14.78 (12.95-17.05)	4.96 (4.43-5.67)
Congo (Brazzaville)	109.80 (96.34-122.52)	6.92 (6.27-7.64)
Costa Rica	302.24 (267.28-341.35)	12.72 (11.63-14.29)
Cuba	882.91 (786.13-1011.83)	7.84 (7.02-8.97)
DR Congo	1240.43 (1084.64-1412.78)	5.14 (4.61-5.83)
Democratic Republic of Sao Tome and Principe	2.76 (2.33-3.30)	3.19 (2.73-3.74)
Djibouti	11.33 (9.62-13.13)	4.66 (4.16-5.27)
Dominica	4.74 (4.26-5.29)	6.61 (5.97-7.43)
Dominican Republic	270.15 (235.26-313.77)	5.25 (4.70-5.93)
Ecuador	919.07 (823.83-1035.69)	12.46 (11.47-13.73)

Egypt	1648.99 (1367.74-1953.32)	3.23 (2.75-3.81)
El Salvador	225.73 (197.22-260.77)	5.42 (4.83-6.14)
Equatorial Guinea	14.99 (13.22-16.91)	5.40 (4.83-6.01)
Eritrea	96.38 (84.52-109.24)	4.67 (4.25-5.22)
Ethiopia	1628.78 (1413.66-1880.31)	5.12 (4.51-5.87)
Fiji	14.82 (12.04-18.06)	2.08 (1.69-2.52)
Gabon	48.97 (43.75-55.12)	7.08 (6.39-7.86)
Gambia	19.42 (16.13-23.53)	2.94 (2.53-3.44)
Georgia	236.28 (202.26-283.76)	4.07 (3.47-4.91)
Ghana	291.69 (243.96-351.82)	2.72 (2.31-3.22)
Grenada	3.42 (2.98-3.96)	3.97 (3.45-4.59)
Guatemala	359.24 (306.46-408.59)	6.70 (5.98-7.51)
Guinea	124.88 (106.37-147.69)	2.82 (2.44-3.29)
Guinea-Bissau	19.46 (16.29-23.23)	2.83 (2.44-3.28)
Haiti	226.57 (196.29-259.55)	4.84 (4.36-5.42)
Honduras	418.72 (373.12-477.96)	13.69 (12.54-15.21)
India	62960.03 (55585.63-72611.20)	10.62 (9.34-12.09)
Indonesia	3189.30 (2674.82-3892.38)	2.05 (1.76-2.48)
Iran	1477.88 (1227.05-1741.79)	3.12 (2.68-3.66)
Iraq	569.12 (469.08-698.43)	3.53 (2.99-4.16)
Jamaica	112.33 (98.38-128.69)	5.41 (4.78-6.16)
Jordan	112.88 (92.11-137.26)	3.41 (2.89-4.02)
Kazakhstan	615.36 (521.28-721.12)	3.73 (3.18-4.36)
Kenya	670.92 (576.05-790.59)	5.16 (4.53-5.93)
Kiribati	1.40 (1.16-1.68)	2.08 (1.76-2.48)
Kyrgyzstan	431.93 (381.20-493.13)	11.68 (10.49-12.92)
Laos	87.13 (74.70-101.85)	2.81 (2.47-3.21)
Lebanon	159.28 (135.09-186.61)	5.09 (4.37-6.01)
Lesotho	89.12 (77.90-100.99)	7.88 (7.07-8.81)
Liberia	57.29 (48.74-69.63)	2.97 (2.56-3.51)
Libya	124.64 (102.82-148.22)	3.23 (2.75-3.82)
Madagascar	300.79 (259.20-350.37)	4.03 (3.61-4.59)
Malawi	289.30 (252.74-334.48)	4.91 (4.42-5.57)
Malaysia	307.94 (257.54-373.16)	2.12 (1.79-2.53)
Maldives	6.63 (5.69-7.61)	4.29 (3.81-4.87)
Mali	157.89 (133.16-187.07)	2.60 (2.21-3.05)
Marshall Islands	0.91 (0.75-1.08)	2.64 (2.27-3.05)
Mauritania	47.84 (40.37-56.64)	3.21 (2.77-3.70)
Mauritius	35.86 (30.68-42.44)	3.62 (3.13-4.22)

Mexico	14427.60 (12837.84-16258.53)	23.57 (20.95-26.68)
Micronesia	2.46 (2.03-2.90)	2.89 (2.48-3.33)
Moldova	197.01 (166.89-230.28)	4.28 (3.61-5.04)
Mongolia	88.28 (74.95-103.02)	5.09 (4.53-5.78)
Montenegro	44.74 (38.19-52.79)	6.84 (5.86-8.04)
Morocco	676.45 (560.85-804.08)	2.86 (2.44-3.36)
Mozambique	406.85 (358.48-464.12)	4.64 (4.20-5.20)
Myanmar	953.18 (818.26-1093.14)	2.84 (2.51-3.20)
Namibia	74.92 (64.13-86.23)	7.27 (6.50-8.13)
Nepal	998.13 (871.34-1140.72)	7.43 (6.64-8.33)
Nicaragua	247.14 (214.43-278.50)	10.40 (9.49-11.44)
Niger	131.01 (107.92-157.14)	2.52 (2.14-2.96)
Nigeria	1847.36 (1545.35-2245.82)	2.70 (2.30-3.23)
North Korea	1826.93 (1619.61-2070.07)	9.12 (8.14-10.35)
North Macedonia	96.17 (79.47-115.44)	4.62 (3.81-5.52)
Pakistan	8630.94 (7471.42-10096.44)	9.82 (8.72-11.22)
Palestine	58.69 (47.84-71.23)	3.57 (3.02-4.19)
Papua New Guinea	71.13 (58.78-86.17)	2.03 (1.73-2.41)
Paraguay	307.02 (262.65-361.86)	8.90 (7.89-10.27)
Peru	2977.00 (2604.12-3377.81)	14.82 (13.30-16.61)
Philippines	2756.81 (2365.27-3245.80)	5.78 (5.09-6.68)
Rwanda	219.39 (191.64-249.77)	5.04 (4.54-5.63)
Saint Lucia	7.86 (7.01-8.88)	7.14 (6.49-7.97)
Saint Vincent and the Grenadines	4.03 (3.44-4.69)	4.37 (3.84-5.10)
Samoa	4.44 (3.67-5.24)	3.07 (2.64-3.60)
Senegal	156.65 (130.17-187.23)	3.04 (2.57-3.53)
Serbia	659.84 (601.26-728.73)	6.05 (5.50-6.67)
Sierra Leone	86.55 (72.65-102.94)	2.84 (2.41-3.32)
Solomon Islands	7.06 (5.88-8.37)	2.60 (2.25-3.00)
Somalia	239.87 (207.17-273.58)	5.01 (4.50-5.58)
South Africa	5872.89 (5147.24-6677.45)	17.18 (15.37-19.44)
South Sudan	156.08 (134.98-182.91)	4.34 (3.84-4.90)
Sri Lanka	349.90 (290.81-421.64)	2.23 (1.89-2.67)
Sudan	425.76 (348.47-509.37)	2.48 (2.08-2.90)
Suriname	12.94 (11.09-15.13)	3.82 (3.34-4.44)
Swaziland	52.96 (45.28-60.80)	9.55 (8.51-10.78)
Syria	345.38 (284.93-414.15)	3.22 (2.70-3.80)
Tajikistan	254.44 (218.85-293.76)	6.08 (5.37-6.88)
Tanzania	784.49 (682.63-905.32)	4.93 (4.38-5.57)

Thailand	1984.64 (1692.02-2283.51)	3.80 (3.29-4.37)
Timor-Leste	14.89 (12.43-17.56)	2.67 (2.33-3.07)
Togo	69.79 (57.91-84.10)	2.96 (2.54-3.48)
Tonga	2.43 (2.03-2.89)	2.89 (2.48-3.37)
Tunisia	247.86 (207.41-294.56)	3.14 (2.63-3.71)
Turkiye	3572.05 (3186.97-4064.88)	6.77 (6.08-7.65)
Turkmenistan	103.98 (85.55-124.30)	3.26 (2.77-3.87)
Tuvalu	0.22 (0.18-0.25)	2.53 (2.15-2.92)
Uganda	457.76 (392.20-529.67)	4.66 (4.15-5.27)
Ukraine	2086.19 (1769.57-2491.95)	3.63 (3.10-4.31)
Uzbekistan	2005.62 (1711.49-2300.55)	11.40 (10.23-12.63)
Vanuatu	3.09 (2.54-3.65)	2.48 (2.14-2.88)
Vietnam	2226.14 (1905.86-2584.50)	4.13 (3.59-4.74)
Yemen	240.16 (197.34-284.21)	2.29 (1.95-2.67)
Zambia	219.08 (190.47-251.61)	4.52 (4.07-4.97)
Zimbabwe	411.74 (345.75-483.08)	5.46 (4.72-6.16)

Abbreviations: LMICs: Low- and Middle-Income Countries; UI: Uncertainty Interval.

Table S8. Rheumatoid arthritis incident cases and age-standardized incidence rates in 129 LMICs, 2023

Location	Incidence cases (95% UI, 2023)	Age-standardized incidence rate (per 100,000, 95% UI, 2023)
Afghanistan	1072.53 (895.72-1245.03)	3.46 (3.02-3.92)
Albania	259.21 (225.72-298.66)	8.64 (7.50-10.00)
Algeria	1872.97 (1561.42-2193.76)	4.08 (3.46-4.76)
Angola	1537.45 (1348.43-1741.56)	6.50 (5.84-7.32)
Argentina	6117.09 (5533.76-6803.57)	11.61 (10.44-12.96)
Armenia	195.99 (170.69-225.59)	5.80 (4.91-6.67)
Azerbaijan	503.35 (429.00-602.87)	4.35 (3.68-5.21)
Bangladesh	13757.05 (12213.15-15819.13)	8.20 (7.32-9.32)
Belarus	735.23 (632.73-860.24)	6.50 (5.63-7.64)
Belize	34.77 (30.86-39.86)	8.91 (8.01-10.07)
Benin	383.37 (321.54-454.63)	3.69 (3.20-4.28)
Bhutan	101.20 (89.06-115.13)	13.54 (12.10-15.42)
Bolivia	2040.30 (1841.83-2282.17)	17.43 (15.90-19.35)
Bosnia and Herzegovina	527.89 (471.05-594.63)	13.04 (11.57-14.51)
Botswana	239.87 (210.58-271.61)	10.14 (9.06-11.34)
Brazil	16909.64 (14607.63-19528.72)	7.57 (6.55-8.69)
Burkina Faso	596.44 (496.38-710.08)	3.46 (3.01-4.04)
Burundi	424.54 (367.30-490.92)	4.73 (4.24-5.32)
Cabo Verde	20.37 (17.31-24.15)	4.05 (3.47-4.78)
Cambodia	741.05 (647.98-852.43)	4.50 (3.93-5.16)
Cameroon	1071.95 (886.45-1261.46)	4.15 (3.58-4.79)
Central African Republic	253.96 (224.93-286.49)	5.84 (5.31-6.52)
Chad	354.90 (290.62-424.44)	2.94 (2.53-3.41)
China	257757.82 (225232.18-294722.20)	13.84 (12.27-15.78)
Colombia	7412.80 (6672.92-8242.97)	12.92 (11.60-14.40)
Comoros	39.70 (34.74-45.24)	5.68 (5.01-6.37)
Congo (Brazzaville)	383.33 (335.52-435.16)	8.23 (7.44-9.18)
Costa Rica	1201.72 (1082.16-1346.87)	20.10 (18.17-22.64)
Cuba	1493.84 (1347.37-1696.41)	10.72 (9.70-11.96)
DR Congo	3740.72 (3247.69-4268.18)	5.83 (5.21-6.55)
Democratic Republic of Sao Tome and Principe	8.18 (6.83-9.59)	4.24 (3.66-4.92)
Djibouti	64.00 (54.94-73.18)	5.68 (5.02-6.43)
Dominica	6.97 (6.31-7.74)	8.22 (7.35-9.22)
Dominican Republic	714.04 (629.07-815.21)	6.69 (5.96-7.55)
Ecuador	3585.80 (3290.25-3937.72)	19.64 (18.00-21.57)

Egypt	5930.68 (5031.04-6871.24)	5.48 (4.71-6.35)
El Salvador	539.49 (472.91-619.43)	8.36 (7.34-9.54)
Equatorial Guinea	92.56 (78.26-106.94)	8.66 (7.77-9.77)
Eritrea	274.50 (242.05-310.90)	5.40 (4.89-6.03)
Ethiopia	3675.63 (3155.15-4286.42)	4.61 (4.05-5.27)
Fiji	24.92 (20.83-29.77)	2.62 (2.20-3.13)
Gabon	155.68 (138.14-173.38)	9.68 (8.68-10.74)
Gambia	71.32 (58.21-85.09)	3.76 (3.22-4.36)
Georgia	192.70 (167.39-221.53)	4.93 (4.22-5.72)
Ghana	1095.50 (923.99-1325.15)	3.92 (3.35-4.62)
Grenada	7.90 (7.11-9.08)	6.02 (5.34-6.90)
Guatemala	1736.96 (1553.03-1961.56)	11.97 (10.87-13.44)
Guinea	342.30 (288.74-408.68)	3.35 (2.88-3.88)
Guinea-Bissau	53.70 (44.58-63.80)	3.37 (2.95-3.87)
Haiti	660.62 (579.75-755.67)	5.81 (5.25-6.50)
Honduras	1973.31 (1790.77-2190.66)	21.18 (19.60-23.52)
India	206960.01 (182390.01-235675.06)	14.78 (13.03-16.82)
Indonesia	8479.79 (7240.28-10096.80)	2.79 (2.41-3.28)
Iran	4114.04 (3502.30-4866.41)	4.26 (3.64-4.97)
Iraq	2257.86 (1904.58-2660.46)	5.16 (4.41-5.99)
Jamaica	234.64 (210.07-266.71)	7.51 (6.73-8.52)
Jordan	742.23 (609.78-870.67)	5.33 (4.45-6.16)
Kazakhstan	1074.85 (928.71-1246.63)	5.25 (4.53-6.05)
Kenya	2261.89 (1972.47-2638.51)	5.84 (5.16-6.69)
Kiribati	3.10 (2.63-3.70)	2.48 (2.12-2.93)
Kyrgyzstan	1158.34 (1039.61-1290.39)	17.48 (16.00-19.23)
Laos	293.68 (252.13-341.10)	4.29 (3.79-4.94)
Lebanon	441.29 (375.25-512.11)	7.35 (6.30-8.45)
Lesotho	165.24 (144.23-189.01)	9.48 (8.51-10.59)
Liberia	183.30 (150.78-218.75)	3.78 (3.24-4.37)
Libya	370.33 (315.51-434.11)	4.62 (3.97-5.35)
Madagascar	973.77 (838.92-1123.58)	4.65 (4.12-5.28)
Malawi	787.31 (682.40-913.02)	5.85 (5.26-6.64)
Malaysia	1050.84 (890.71-1254.73)	2.88 (2.47-3.38)
Maldives	31.25 (26.55-36.64)	5.38 (4.72-6.11)
Mali	527.93 (437.37-628.65)	3.14 (2.71-3.60)
Marshall Islands	1.41 (1.19-1.60)	3.49 (3.01-3.98)
Mauritania	161.95 (136.36-187.86)	4.60 (3.97-5.27)
Mauritius	90.15 (79.83-104.49)	5.55 (4.87-6.39)

Mexico	35380.45 (31660.64-39992.91)	24.86 (22.30-28.13)
Micronesia	4.29 (3.64-4.90)	4.10 (3.48-4.69)
Moldova	268.36 (235.00-311.56)	6.16 (5.41-7.21)
Mongolia	242.01 (211.86-277.34)	7.27 (6.42-8.23)
Montenegro	68.43 (60.37-78.77)	8.73 (7.54-10.11)
Morocco	1749.32 (1504.30-2022.44)	4.43 (3.80-5.12)
Mozambique	1126.78 (984.09-1284.73)	5.57 (5.00-6.21)
Myanmar	2619.31 (2302.15-3048.43)	4.56 (4.02-5.26)
Namibia	236.47 (204.64-269.32)	9.16 (8.22-10.27)
Nepal	3549.28 (3167.81-4039.53)	11.96 (10.67-13.51)
Nicaragua	889.78 (799.66-997.73)	14.36 (12.98-15.84)
Niger	498.22 (411.63-593.89)	2.96 (2.55-3.42)
Nigeria	6377.96 (5329.10-7642.21)	3.42 (2.94-4.03)
North Korea	4151.52 (3738.16-4636.88)	12.52 (11.34-14.00)
North Macedonia	147.77 (125.82-174.56)	6.76 (5.68-7.99)
Pakistan	16670.00 (14467.43-19484.17)	8.14 (7.19-9.41)
Palestine	266.41 (221.77-315.36)	5.23 (4.51-6.05)
Papua New Guinea	245.23 (205.41-288.21)	2.44 (2.10-2.83)
Paraguay	1031.29 (930.44-1168.84)	15.58 (14.09-17.52)
Peru	9378.50 (8337.70-10598.32)	24.88 (22.11-27.98)
Philippines	5623.27 (4927.00-6554.30)	5.32 (4.70-6.16)
Rwanda	618.37 (532.39-707.06)	6.00 (5.30-6.80)
Saint Lucia	20.44 (18.64-22.84)	9.34 (8.48-10.41)
Saint Vincent and the Grenadines	7.54 (6.76-8.70)	5.66 (5.02-6.56)
Samoa	7.75 (6.59-8.88)	3.96 (3.40-4.55)
Senegal	534.56 (442.59-634.38)	3.80 (3.25-4.41)
Serbia	1032.47 (909.69-1186.42)	8.94 (7.86-10.32)
Sierra Leone	214.06 (178.70-255.72)	3.33 (2.83-3.88)
Solomon Islands	27.28 (23.16-31.74)	3.42 (2.98-3.93)
Somalia	665.82 (584.82-763.61)	5.02 (4.56-5.58)
South Africa	10047.88 (8876.27-11397.05)	14.59 (13.10-16.54)
South Sudan	344.15 (291.23-397.37)	4.97 (4.41-5.63)
Sri Lanka	797.11 (681.16-948.95)	3.13 (2.64-3.74)
Sudan	1414.57 (1183.27-1674.58)	3.82 (3.25-4.44)
Suriname	30.48 (26.69-35.47)	4.91 (4.26-5.69)
Swaziland	117.25 (102.38-133.51)	10.85 (9.77-12.06)
Syria	947.79 (810.04-1131.50)	5.19 (4.42-6.19)
Tajikistan	720.74 (622.64-823.29)	7.93 (7.09-8.94)
Tanzania	2631.32 (2289.83-3001.96)	5.98 (5.31-6.76)

Thailand	4691.03 (4034.81-5342.50)	5.38 (4.66-6.13)
Timor-Leste	45.87 (39.88-53.34)	3.99 (3.51-4.58)
Togo	251.90 (213.25-297.59)	3.64 (3.19-4.24)
Tonga	3.76 (3.17-4.40)	3.80 (3.21-4.43)
Tunisia	616.37 (527.86-722.76)	4.83 (4.13-5.71)
Turkiye	10217.83 (9163.95-11495.12)	10.66 (9.57-12.00)
Turkmenistan	249.10 (213.45-289.27)	4.45 (3.84-5.15)
Tuvalu	0.37 (0.32-0.43)	3.41 (2.93-3.92)
Uganda	1571.46 (1365.18-1819.56)	5.73 (5.17-6.40)
Ukraine	2390.30 (2035.68-2823.47)	4.77 (4.01-5.62)
Uzbekistan	5046.02 (4494.47-5665.46)	13.78 (12.29-15.35)
Vanuatu	9.51 (8.03-11.01)	3.15 (2.73-3.62)
Vietnam	8288.40 (7293.99-9421.94)	7.21 (6.39-8.22)
Yemen	986.75 (812.50-1168.33)	3.06 (2.61-3.60)
Zambia	768.18 (658.52-881.64)	5.67 (5.11-6.31)
Zimbabwe	751.50 (642.43-861.19)	5.64 (4.94-6.37)

Abbreviations: LMICs: Low- and Middle-Income Countries; UI: Uncertainty Interval.

Table S9. Percentage change in incident cases and EAPC in age-standardized incidence rates of rheumatoid arthritis in 129 LMICs, 1990–2023

Location	Incidence PC (%, 1990–2023)	Incidence EAPC (%, 95% CI, 1990–2023)
Afghanistan	362	0.81 (0.63 to 0.98)
Albania	70	1.82 (1.75 to 1.89)
Algeria	245	1.56 (1.48 to 1.64)
Angola	343	0.84 (0.76 to 0.92)
Argentina	129	0.99 (0.90 to 1.09)
Armenia	53	1.43 (1.39 to 1.48)
Azerbaijan	116	1.13 (0.88 to 1.39)
Bangladesh	233	1.67 (1.50 to 1.84)
Belarus	59	1.50 (1.38 to 1.62)
Belize	297	1.01 (0.97 to 1.06)
Benin	306	0.76 (0.68 to 0.84)
Bhutan	193	1.61 (1.52 to 1.70)
Bolivia	285	1.45 (1.42 to 1.48)
Bosnia and Herzegovina	26	1.51 (1.41 to 1.60)
Botswana	228	0.98 (0.85 to 1.11)
Brazil	51	0.10 (0.06 to 0.15)
Burkina Faso	228	0.67 (0.61 to 0.74)
Burundi	171	0.25 (0.19 to 0.31)
Cabo Verde	169	1.10 (1.01 to 1.19)
Cambodia	266	1.50 (1.46 to 1.53)
Cameroon	324	0.68 (0.53 to 0.83)
Central African Republic	143	0.17 (0.13 to 0.22)
Chad	238	0.47 (0.35 to 0.59)
China	101	0.62 (0.59 to 0.64)
Colombia	205	1.16 (1.03 to 1.30)
Comoros	169	0.57 (0.49 to 0.65)
Congo (Brazzaville)	249	0.79 (0.70 to 0.88)
Costa Rica	298	1.35 (1.27 to 1.43)
Cuba	69	0.97 (0.91 to 1.03)
DR Congo	202	0.49 (0.39 to 0.59)
Democratic Republic of Sao Tome and Principe	196	0.96 (0.85 to 1.07)
Djibouti	465	0.74 (0.64 to 0.84)
Dominica	47	0.61 (0.55 to 0.67)
Dominican Republic	164	0.70 (0.63 to 0.77)
Ecuador	290	1.34 (1.26 to 1.42)

Egypt	260	1.54 (1.46 to 1.62)
El Salvador	139	1.41 (1.30 to 1.51)
Equatorial Guinea	517	1.89 (1.71 to 2.06)
Eritrea	185	0.50 (0.48 to 0.53)
Ethiopia	126	-0.09 (-0.16 to -0.01)
Fiji	68	0.74 (0.60 to 0.88)
Gabon	218	1.10 (1.05 to 1.15)
Gambia	267	0.84 (0.77 to 0.92)
Georgia	-18	0.57 (0.49 to 0.66)
Ghana	276	1.15 (1.04 to 1.26)
Grenada	131	1.07 (0.97 to 1.16)
Guatemala	384	1.64 (1.53 to 1.76)
Guinea	174	0.57 (0.47 to 0.67)
Guinea-Bissau	176	0.60 (0.50 to 0.70)
Haiti	192	0.58 (0.54 to 0.63)
Honduras	371	1.27 (1.20 to 1.33)
India	229	1.18 (1.10 to 1.26)
Indonesia	166	0.83 (0.76 to 0.89)
Iran	178	0.97 (0.93 to 1.00)
Iraq	297	1.40 (1.24 to 1.56)
Jamaica	109	0.91 (0.83 to 0.99)
Jordan	558	1.63 (1.54 to 1.72)
Kazakhstan	75	1.32 (1.10 to 1.55)
Kenya	237	0.34 (0.27 to 0.41)
Kiribati	122	0.50 (0.44 to 0.57)
Kyrgyzstan	168	1.32 (1.23 to 1.41)
Laos	237	1.38 (1.31 to 1.44)
Lebanon	177	1.27 (1.16 to 1.38)
Lesotho	85	0.43 (0.31 to 0.54)
Liberia	220	0.94 (0.81 to 1.07)
Libya	197	1.22 (1.16 to 1.28)
Madagascar	224	0.50 (0.46 to 0.54)
Malawi	172	0.67 (0.61 to 0.74)
Malaysia	241	1.00 (0.96 to 1.04)
Maldives	371	0.95 (0.64 to 1.27)
Mali	234	0.68 (0.60 to 0.76)
Marshall Islands	54	0.86 (0.80 to 0.91)
Mauritania	239	1.15 (1.06 to 1.24)
Mauritius	151	1.13 (1.06 to 1.20)

Mexico	145	0.17 (0.10 to 0.24)
Micronesia	74	1.03 (1.00 to 1.07)
Moldova	36	1.23 (1.08 to 1.38)
Mongolia	174	1.44 (1.31 to 1.57)
Montenegro	53	1.01 (0.87 to 1.15)
Morocco	159	1.40 (1.30 to 1.50)
Mozambique	177	0.53 (0.48 to 0.58)
Myanmar	175	1.52 (1.44 to 1.60)
Namibia	216	0.83 (0.73 to 0.94)
Nepal	256	1.54 (1.50 to 1.57)
Nicaragua	260	1.14 (1.10 to 1.19)
Niger	280	0.60 (0.52 to 0.68)
Nigeria	245	0.81 (0.67 to 0.94)
North Korea	127	1.02 (0.97 to 1.06)
North Macedonia	54	1.33 (1.26 to 1.41)
Pakistan	93	0.00 (-0.17 to 0.17)
Palestine	354	1.20 (1.12 to 1.28)
Papua New Guinea	245	0.51 (0.44 to 0.58)
Paraguay	236	1.47 (1.36 to 1.59)
Peru	215	1.80 (1.71 to 1.88)
Philippines	104	-0.17 (-0.21 to -0.14)
Rwanda	182	0.94 (0.82 to 1.07)
Saint Lucia	160	0.80 (0.68 to 0.93)
Saint Vincent and the Grenadines	87	0.82 (0.79 to 0.85)
Samoa	74	0.69 (0.64 to 0.74)
Senegal	241	0.70 (0.62 to 0.77)
Serbia	56	1.34 (1.24 to 1.45)
Sierra Leone	147	0.52 (0.40 to 0.63)
Solomon Islands	286	0.78 (0.74 to 0.82)
Somalia	178	0.08 (0.05 to 0.11)
South Africa	71	-0.25 (-0.34 to -0.17)
South Sudan	120	0.51 (0.47 to 0.55)
Sri Lanka	128	1.15 (1.03 to 1.27)
Sudan	232	1.45 (1.31 to 1.59)
Suriname	135	0.85 (0.78 to 0.92)
Swaziland	121	0.44 (0.29 to 0.58)
Syria	174	1.59 (1.49 to 1.70)
Tajikistan	183	0.77 (0.72 to 0.82)
Tanzania	235	0.62 (0.56 to 0.67)

Thailand	136	1.23 (1.14 to 1.32)
Timor-Leste	208	1.23 (1.21 to 1.26)
Togo	261	0.70 (0.58 to 0.82)
Tonga	55	0.74 (0.68 to 0.80)
Tunisia	149	1.46 (1.36 to 1.56)
Turkiye	186	1.64 (1.56 to 1.73)
Turkmenistan	140	1.11 (0.97 to 1.25)
Tuvalu	69	0.87 (0.83 to 0.90)
Uganda	243	0.81 (0.72 to 0.90)
Ukraine	15	0.75 (0.71 to 0.78)
Uzbekistan	152	0.82 (0.72 to 0.91)
Vanuatu	208	0.70 (0.66 to 0.74)
Vietnam	272	1.68 (1.64 to 1.72)
Yemen	311	1.02 (0.94 to 1.11)
Zambia	251	0.90 (0.76 to 1.03)
Zimbabwe	83	-0.15 (-0.31 to 0.00)

Notes: PC was calculated from all-age counts between 1990 and 2023, whereas EAPC was calculated from age-standardized rates over the 1990-2023 time series. Abbreviations: LMICs: Low- and Middle-Income Countries; PC: Percentage Change; EAPC: Estimated Annual Percentage Change; CI: Confidence Interval.

Table S10. Rheumatoid arthritis deaths and age-standardized death rates in 129 LMICs, 1990

Location	Deaths (95% UI, 1990)	Age-Standardized death rate (per 100,000, 95% UI, 1990)
Afghanistan	4.23 (1.09-10.11)	0.06 (0.02-0.15)
Albania	6.55 (3.73-10.00)	0.30 (0.17-0.47)
Algeria	5.92 (2.25-15.68)	0.05 (0.02-0.13)
Angola	31.90 (15.06-50.69)	0.83 (0.40-1.31)
Argentina	129.93 (111.46-153.81)	0.41 (0.35-0.48)
Armenia	1.16 (0.95-1.47)	0.05 (0.04-0.06)
Azerbaijan	1.63 (0.97-2.74)	0.04 (0.02-0.06)
Bangladesh	1038.23 (395.85-2445.24)	2.66 (0.99-6.44)
Belarus	4.79 (3.92-5.91)	0.04 (0.03-0.05)
Belize	0.25 (0.20-0.32)	0.28 (0.22-0.35)
Benin	8.48 (4.55-13.98)	0.40 (0.22-0.62)
Bhutan	1.76 (0.68-2.98)	0.96 (0.38-1.63)
Bolivia	11.71 (6.97-21.28)	0.40 (0.24-0.71)
Bosnia and Herzegovina	10.45 (6.00-15.23)	0.28 (0.16-0.42)
Botswana	3.78 (2.15-5.28)	0.60 (0.35-0.84)
Brazil	230.64 (204.63-261.22)	0.27 (0.24-0.30)
Burkina Faso	21.13 (11.41-33.99)	0.51 (0.29-0.80)
Burundi	38.43 (24.27-56.29)	1.55 (0.97-2.24)
Cabo Verde	0.64 (0.36-1.05)	0.29 (0.16-0.48)
Cambodia	8.81 (3.31-16.08)	0.25 (0.10-0.44)
Cameroon	23.54 (12.17-40.54)	0.56 (0.32-0.88)
Central African Republic	7.30 (3.30-12.48)	0.67 (0.30-1.12)
Chad	12.66 (6.88-21.12)	0.46 (0.27-0.74)
China	5567.22 (3466.20-7778.55)	0.75 (0.47-1.05)
Colombia	127.85 (114.92-144.13)	0.80 (0.72-0.90)
Comoros	2.83 (1.87-4.08)	1.20 (0.79-1.74)
Congo (Brazzaville)	9.28 (4.33-15.56)	0.92 (0.44-1.51)
Costa Rica	12.21 (10.11-14.86)	0.77 (0.64-0.93)
Cuba	40.69 (33.89-48.40)	0.39 (0.32-0.46)
DR Congo	118.47 (53.70-199.36)	0.79 (0.37-1.25)
Democratic Republic of Sao Tome and Principe	0.27 (0.15-0.44)	0.42 (0.24-0.67)
Djibouti	2.01 (1.29-2.87)	1.65 (1.12-2.28)
Dominica	0.15 (0.09-0.22)	0.21 (0.13-0.32)
Dominican Republic	1.81 (0.58-5.33)	0.05 (0.01-0.14)
Ecuador	41.02 (35.08-47.94)	0.87 (0.75-1.02)

Egypt	19.51 (9.28-40.24)	0.08 (0.04-0.17)
El Salvador	8.85 (4.87-13.64)	0.31 (0.17-0.48)
Equatorial Guinea	1.68 (0.78-2.68)	0.93 (0.43-1.48)
Eritrea	21.07 (13.23-30.28)	1.71 (1.11-2.45)
Ethiopia	448.01 (271.45-649.98)	2.11 (1.29-3.19)
Fiji	0.00 (0.00-0.05)	0.00 (0.00-0.02)
Gabon	4.52 (2.08-7.17)	0.87 (0.41-1.37)
Gambia	1.29 (0.69-2.16)	0.34 (0.20-0.53)
Georgia	1.16 (0.95-1.44)	0.02 (0.02-0.03)
Ghana	29.22 (15.42-51.91)	0.51 (0.28-0.91)
Grenada	0.19 (0.14-0.24)	0.24 (0.19-0.31)
Guatemala	25.04 (21.04-29.25)	0.70 (0.59-0.81)
Guinea	14.92 (8.01-24.56)	0.47 (0.27-0.73)
Guinea-Bissau	2.25 (1.16-3.78)	0.48 (0.28-0.78)
Haiti	11.02 (4.73-20.24)	0.33 (0.15-0.60)
Honduras	20.80 (8.31-33.57)	1.18 (0.48-1.92)
India	3466.21 (1168.68-6159.27)	1.04 (0.35-1.89)
Indonesia	122.02 (55.23-240.62)	0.15 (0.07-0.30)
Iran	100.33 (51.45-159.34)	0.39 (0.20-0.61)
Iraq	1.17 (0.34-5.91)	0.01 (0.00-0.08)
Jamaica	4.77 (3.87-5.89)	0.26 (0.21-0.32)
Jordan	0.63 (0.22-1.90)	0.05 (0.02-0.16)
Kazakhstan	9.99 (8.11-12.26)	0.09 (0.07-0.11)
Kenya	109.86 (70.99-154.29)	1.46 (0.96-2.05)
Kiribati	0.00 (0.00-0.01)	0.00 (0.00-0.03)
Kyrgyzstan	9.82 (7.65-12.69)	0.40 (0.31-0.51)
Laos	4.84 (2.08-8.90)	0.26 (0.11-0.49)
Lebanon	3.05 (1.30-6.34)	0.14 (0.06-0.29)
Lesotho	6.51 (3.89-9.76)	0.79 (0.48-1.17)
Liberia	5.20 (2.89-8.49)	0.45 (0.26-0.69)
Libya	1.00 (0.39-2.65)	0.05 (0.02-0.14)
Madagascar	102.23 (64.73-146.80)	2.32 (1.51-3.37)
Malawi	70.25 (45.64-100.65)	1.87 (1.22-2.68)
Malaysia	5.44 (2.07-20.30)	0.08 (0.03-0.25)
Maldives	0.22 (0.12-0.38)	0.28 (0.16-0.45)
Mali	16.97 (8.76-27.85)	0.45 (0.25-0.70)
Marshall Islands	0.00 (0.00-0.01)	0.00 (0.00-0.04)
Mauritania	5.64 (3.38-9.29)	0.59 (0.36-0.94)
Mauritius	0.07 (0.05-0.08)	0.01 (0.01-0.02)

Mexico	783.84 (697.91-875.33)	1.98 (1.77-2.20)
Micronesia	0.01 (0.00-0.03)	0.01 (0.00-0.03)
Moldova	9.16 (7.62-11.24)	0.20 (0.17-0.25)
Mongolia	1.22 (0.33-3.65)	0.13 (0.03-0.39)
Montenegro	1.36 (0.80-1.97)	0.25 (0.15-0.36)
Morocco	8.24 (2.65-20.02)	0.06 (0.02-0.14)
Mozambique	81.15 (52.68-113.90)	1.34 (0.87-1.88)
Myanmar	63.36 (28.32-113.07)	0.34 (0.15-0.59)
Namibia	6.34 (3.71-8.87)	0.94 (0.57-1.29)
Nepal	60.27 (27.16-99.35)	0.88 (0.39-1.47)
Nicaragua	10.60 (5.83-15.81)	0.79 (0.44-1.15)
Niger	12.77 (6.00-22.15)	0.48 (0.26-0.77)
Nigeria	160.68 (88.63-265.04)	0.39 (0.23-0.62)
North Korea	73.01 (44.23-108.23)	0.59 (0.37-0.87)
North Macedonia	0.80 (0.33-1.87)	0.05 (0.02-0.11)
Pakistan	426.97 (187.96-687.87)	0.91 (0.40-1.47)
Palestine	0.49 (0.22-1.22)	0.05 (0.02-0.14)
Papua New Guinea	0.05 (0.01-0.37)	0.00 (0.00-0.02)
Paraguay	8.83 (5.29-15.14)	0.40 (0.24-0.67)
Peru	16.47 (9.81-27.88)	0.14 (0.09-0.24)
Philippines	89.25 (44.27-132.06)	0.41 (0.20-0.62)
Rwanda	66.18 (42.41-96.32)	2.16 (1.43-3.10)
Saint Lucia	0.20 (0.16-0.24)	0.21 (0.17-0.26)
Saint Vincent and the Grenadines	0.02 (0.01-0.02)	0.02 (0.02-0.03)
Samoa	0.00 (0.00-0.02)	0.00 (0.00-0.02)
Senegal	12.95 (6.50-21.64)	0.38 (0.21-0.59)
Serbia	28.86 (15.99-46.35)	0.29 (0.16-0.46)
Sierra Leone	34.03 (16.68-61.70)	1.93 (0.92-3.59)
Solomon Islands	0.00 (0.00-0.03)	0.00 (0.00-0.02)
Somalia	28.57 (16.92-48.67)	1.12 (0.67-1.87)
South Africa	188.26 (99.32-265.99)	0.94 (0.50-1.32)
South Sudan	48.81 (30.97-73.39)	2.13 (1.35-3.15)
Sri Lanka	4.63 (2.63-10.01)	0.05 (0.03-0.11)
Sudan	4.54 (1.49-10.54)	0.05 (0.02-0.12)
Suriname	0.29 (0.14-0.83)	0.12 (0.06-0.35)
Swaziland	3.62 (2.12-5.27)	1.08 (0.63-1.52)
Syria	0.09 (0.02-0.76)	0.00 (0.00-0.02)
Tajikistan	4.01 (2.09-7.53)	0.16 (0.08-0.30)
Tanzania	167.17 (110.86-237.87)	1.50 (1.00-2.16)

Thailand	90.58 (43.04-170.31)	0.30 (0.15-0.58)
Timor-Leste	0.55 (0.21-1.04)	0.31 (0.12-0.57)
Togo	5.78 (2.94-10.34)	0.45 (0.25-0.73)
Tonga	0.00 (0.00-0.00)	0.00 (0.00-0.01)
Tunisia	17.28 (8.23-28.87)	0.41 (0.19-0.71)
Turkiye	154.53 (77.59-257.81)	0.47 (0.23-0.79)
Turkmenistan	0.09 (0.07-0.11)	0.00 (0.00-0.01)
Tuvalu	0.00 (0.00-0.00)	0.00 (0.00-0.03)
Uganda	96.61 (62.57-143.32)	1.57 (1.03-2.31)
Ukraine	92.73 (76.51-112.71)	0.13 (0.11-0.16)
Uzbekistan	59.03 (50.47-71.11)	0.53 (0.45-0.64)
Vanuatu	0.00 (0.00-0.01)	0.00 (0.00-0.02)
Vietnam	87.50 (36.79-146.40)	0.24 (0.10-0.39)
Yemen	2.20 (0.79-5.21)	0.05 (0.02-0.11)
Zambia	55.66 (36.47-78.78)	2.21 (1.42-3.20)
Zimbabwe	22.30 (14.09-35.11)	0.51 (0.33-0.79)

Abbreviations: LMICs: Low- and Middle-Income Countries; UI: Uncertainty Interval.

Table S11. Rheumatoid arthritis deaths and age-standardized death rates in 129 LMICs, 2023

Location	Deaths (95% UI, 2023)	Age-standardized death rate (per 100,000, 95% UI, 2023)
Afghanistan	11.77 (3.59-24.90)	0.10 (0.03-0.20)
Albania	7.75 (3.86-13.26)	0.17 (0.09-0.30)
Algeria	26.17 (9.30-48.34)	0.07 (0.02-0.14)
Angola	94.22 (53.96-146.49)	0.72 (0.41-1.09)
Argentina	209.05 (175.68-246.18)	0.34 (0.29-0.40)
Armenia	8.40 (6.61-10.36)	0.16 (0.13-0.20)
Azerbaijan	3.26 (1.85-5.58)	0.04 (0.02-0.06)
Bangladesh	1895.42 (779.00-3818.23)	1.96 (0.76-4.10)
Belarus	5.74 (4.65-7.05)	0.04 (0.03-0.04)
Belize	0.97 (0.75-1.25)	0.32 (0.25-0.41)
Benin	21.27 (10.52-37.09)	0.35 (0.19-0.58)
Bhutan	4.81 (2.40-8.17)	0.81 (0.40-1.38)
Bolivia	58.01 (27.66-92.97)	0.65 (0.31-1.04)
Bosnia and Herzegovina	13.35 (8.48-20.02)	0.21 (0.13-0.31)
Botswana	8.16 (5.11-12.03)	0.47 (0.30-0.70)
Brazil	566.04 (496.08-650.87)	0.22 (0.19-0.25)
Burkina Faso	40.80 (23.07-63.49)	0.40 (0.23-0.61)
Burundi	64.15 (41.89-91.45)	1.30 (0.85-1.86)
Cabo Verde	1.06 (0.53-1.75)	0.25 (0.13-0.40)
Cambodia	26.17 (9.83-46.53)	0.23 (0.08-0.40)
Cameroon	59.34 (31.74-104.08)	0.43 (0.26-0.68)
Central African Republic	13.28 (6.71-22.60)	0.54 (0.28-0.89)
Chad	23.19 (11.68-39.32)	0.35 (0.19-0.57)
China	9456.38 (5825.20-12473.95)	0.43 (0.26-0.57)
Colombia	302.71 (262.49-347.93)	0.51 (0.44-0.58)
Comoros	6.50 (3.95-9.38)	0.98 (0.61-1.41)
Congo (Brazzaville)	18.66 (10.35-29.43)	0.75 (0.42-1.15)
Costa Rica	44.55 (35.91-55.23)	0.75 (0.61-0.93)
Cuba	117.33 (97.41-146.80)	0.57 (0.48-0.72)
DR Congo	260.53 (150.23-425.99)	0.69 (0.41-1.11)
Democratic Republic of Sao Tome and Principe	0.47 (0.24-0.82)	0.38 (0.21-0.61)
Djibouti	8.72 (5.69-12.62)	1.38 (0.88-2.04)
Dominica	0.22 (0.13-0.32)	0.21 (0.13-0.32)
Dominican Republic	18.68 (5.75-34.35)	0.19 (0.06-0.36)
Ecuador	111.84 (94.19-134.77)	0.64 (0.54-0.77)

Egypt	41.52 (20.78-73.80)	0.07 (0.03-0.12)
El Salvador	19.04 (10.23-31.23)	0.32 (0.17-0.52)
Equatorial Guinea	4.41 (2.41-7.30)	0.85 (0.48-1.38)
Eritrea	52.96 (34.74-76.58)	1.55 (1.01-2.21)
Ethiopia	1101.56 (721.02-1579.36)	1.83 (1.20-2.66)
Fiji	0.01 (0.00-0.13)	0.00 (0.00-0.02)
Gabon	7.90 (4.50-12.68)	0.67 (0.38-1.07)
Gambia	3.66 (1.99-6.31)	0.30 (0.17-0.48)
Georgia	4.72 (3.68-5.98)	0.08 (0.06-0.10)
Ghana	85.31 (48.73-154.63)	0.51 (0.28-0.88)
Grenada	0.43 (0.34-0.55)	0.28 (0.22-0.35)
Guatemala	71.56 (57.80-86.08)	0.70 (0.58-0.84)
Guinea	23.56 (12.83-40.62)	0.36 (0.21-0.59)
Guinea-Bissau	4.00 (2.01-7.37)	0.44 (0.25-0.72)
Haiti	26.69 (10.71-48.72)	0.36 (0.15-0.67)
Honduras	75.71 (34.67-116.56)	1.23 (0.57-1.93)
India	11273.45 (4903.69-18777.47)	1.06 (0.46-1.80)
Indonesia	309.28 (138.34-608.21)	0.14 (0.06-0.28)
Iran	135.28 (70.49-241.80)	0.18 (0.09-0.33)
Iraq	5.32 (1.51-19.68)	0.02 (0.01-0.09)
Jamaica	10.81 (8.43-13.98)	0.33 (0.26-0.43)
Jordan	3.13 (1.27-6.59)	0.04 (0.02-0.09)
Kazakhstan	21.45 (16.98-26.88)	0.12 (0.09-0.15)
Kenya	296.91 (185.17-422.53)	1.23 (0.77-1.76)
Kiribati	0.00 (0.00-0.02)	0.00 (0.00-0.02)
Kyrgyzstan	25.98 (20.97-33.07)	0.61 (0.49-0.77)
Laos	11.40 (4.52-20.78)	0.27 (0.11-0.51)
Lebanon	6.80 (3.23-11.74)	0.11 (0.05-0.19)
Lesotho	9.39 (5.58-13.14)	0.74 (0.44-1.03)
Liberia	9.35 (4.85-15.96)	0.36 (0.21-0.57)
Libya	3.62 (1.51-6.96)	0.06 (0.03-0.12)
Madagascar	187.42 (125.72-270.61)	1.80 (1.21-2.65)
Malawi	124.69 (86.98-180.11)	1.59 (1.12-2.25)
Malaysia	16.72 (6.78-67.73)	0.06 (0.03-0.23)
Maldives	0.65 (0.38-0.97)	0.29 (0.17-0.43)
Mali	32.20 (18.28-55.19)	0.33 (0.19-0.51)
Marshall Islands	0.00 (0.00-0.01)	0.00 (0.00-0.04)
Mauritania	10.19 (5.44-17.40)	0.46 (0.26-0.78)
Mauritius	6.29 (5.13-7.61)	0.37 (0.30-0.45)

Mexico	1697.46 (1466.91-1950.39)	1.27 (1.09-1.45)
Micronesia	0.01 (0.00-0.03)	0.01 (0.00-0.03)
Moldova	11.28 (9.17-14.27)	0.18 (0.15-0.23)
Mongolia	5.06 (2.25-8.08)	0.24 (0.11-0.39)
Montenegro	2.38 (1.39-3.43)	0.21 (0.12-0.30)
Morocco	26.02 (9.76-49.78)	0.07 (0.03-0.14)
Mozambique	113.54 (78.17-164.83)	0.87 (0.60-1.28)
Myanmar	122.38 (48.10-221.93)	0.29 (0.12-0.52)
Namibia	13.27 (8.22-19.07)	0.78 (0.48-1.10)
Nepal	165.74 (78.02-283.52)	0.74 (0.34-1.30)
Nicaragua	29.82 (16.66-47.28)	0.71 (0.39-1.15)
Niger	31.96 (16.63-53.51)	0.37 (0.21-0.60)
Nigeria	324.54 (178.08-545.16)	0.34 (0.18-0.53)
North Korea	167.63 (100.62-258.57)	0.57 (0.35-0.86)
North Macedonia	0.99 (0.43-3.22)	0.03 (0.01-0.10)
Pakistan	912.80 (427.89-1529.20)	0.79 (0.37-1.32)
Palestine	1.22 (0.56-3.29)	0.05 (0.02-0.13)
Papua New Guinea	0.18 (0.02-1.13)	0.00 (0.00-0.02)
Paraguay	40.67 (17.94-59.61)	0.75 (0.34-1.10)
Peru	98.05 (25.69-160.56)	0.27 (0.07-0.44)
Philippines	230.41 (124.27-373.48)	0.36 (0.19-0.54)
Rwanda	106.29 (69.38-153.61)	1.68 (1.11-2.45)
Saint Lucia	0.84 (0.67-1.04)	0.34 (0.27-0.43)
Saint Vincent and the Grenadines	0.10 (0.08-0.14)	0.06 (0.05-0.09)
Samoa	0.01 (0.00-0.04)	0.00 (0.00-0.03)
Senegal	33.51 (18.15-56.98)	0.37 (0.21-0.59)
Serbia	36.23 (22.30-55.47)	0.21 (0.13-0.33)
Sierra Leone	44.01 (26.39-74.87)	1.42 (0.83-2.57)
Solomon Islands	0.02 (0.00-0.11)	0.00 (0.00-0.02)
Somalia	86.49 (55.24-125.14)	1.01 (0.65-1.47)
South Africa	326.90 (204.62-482.71)	0.66 (0.41-0.99)
South Sudan	64.58 (42.88-99.83)	1.66 (1.08-2.63)
Sri Lanka	13.78 (6.85-28.49)	0.05 (0.02-0.10)
Sudan	16.67 (6.37-32.13)	0.07 (0.03-0.14)
Suriname	0.68 (0.35-2.06)	0.11 (0.06-0.33)
Swaziland	5.22 (3.20-7.76)	0.70 (0.43-1.01)
Syria	0.16 (0.02-1.57)	0.00 (0.00-0.01)
Tajikistan	13.87 (7.08-21.06)	0.29 (0.15-0.45)
Tanzania	355.59 (230.06-506.21)	1.22 (0.79-1.74)

Thailand	288.37 (160.98-532.79)	0.25 (0.14-0.46)
Timor-Leste	1.92 (0.69-3.57)	0.25 (0.09-0.46)
Togo	15.36 (7.93-26.47)	0.38 (0.21-0.62)
Tonga	0.00 (0.00-0.01)	0.00 (0.00-0.01)
Tunisia	25.97 (12.44-41.00)	0.20 (0.09-0.32)
Turkiye	281.02 (166.75-437.60)	0.29 (0.17-0.46)
Turkmenistan	3.10 (2.49-3.84)	0.08 (0.07-0.10)
Tuvalu	0.00 (0.00-0.00)	0.00 (0.00-0.03)
Uganda	208.99 (136.15-299.96)	1.31 (0.87-1.93)
Ukraine	135.58 (108.97-168.62)	0.18 (0.15-0.23)
Uzbekistan	108.17 (89.64-130.36)	0.48 (0.40-0.58)
Vanuatu	0.01 (0.00-0.04)	0.00 (0.00-0.02)
Vietnam	223.17 (86.89-370.42)	0.21 (0.08-0.35)
Yemen	9.95 (4.22-17.76)	0.07 (0.03-0.12)
Zambia	113.98 (75.11-163.84)	1.80 (1.19-2.54)
Zimbabwe	44.63 (28.75-69.17)	0.50 (0.32-0.77)

Abbreviations: LMICs: Low- and Middle-Income Countries; UI: Uncertainty Interval.

Table S12. Percentage change in deaths and EAPC in age-standardized death rates of rheumatoid arthritis in 129 LMICs, 1990–2023

Location	Deaths PC (%, 1990–2023)	Deaths EAPC (%, 95% CI, 1990–2023)
Afghanistan	178	1.70 (1.43 to 1.98)
Albania	18	-1.37 (-1.71 to -1.04)
Algeria	342	1.52 (1.23 to 1.82)
Angola	195	-0.34 (-0.42 to -0.27)
Argentina	61	0.05 (-0.38 to 0.47)
Armenia	626	5.36 (4.13 to 6.61)
Azerbaijan	100	0.43 (0.22 to 0.63)
Bangladesh	83	-1.10 (-1.24 to -0.97)
Belarus	20	-0.44 (-0.76 to -0.11)
Belize	284	0.34 (0.08 to 0.60)
Benin	151	-0.58 (-0.68 to -0.49)
Bhutan	173	-0.70 (-0.78 to -0.62)
Bolivia	395	1.55 (1.32 to 1.78)
Bosnia and Herzegovina	28	-1.01 (-1.09 to -0.93)
Botswana	116	-1.07 (-1.31 to -0.84)
Brazil	145	-0.22 (-0.48 to 0.04)
Burkina Faso	93	-0.63 (-0.70 to -0.57)
Burundi	67	-0.53 (-0.59 to -0.47)
Cabo Verde	65	-0.10 (-0.33 to 0.14)
Cambodia	197	-0.32 (-0.35 to -0.29)
Cameroon	152	-0.77 (-0.85 to -0.69)
Central African Republic	82	-1.02 (-1.15 to -0.88)
Chad	83	-0.83 (-0.89 to -0.78)
China	70	-1.71 (-1.86 to -1.56)
Colombia	137	-1.06 (-1.51 to -0.62)
Comoros	130	-0.75 (-0.82 to -0.68)
Congo (Brazzaville)	101	-0.67 (-0.74 to -0.59)
Costa Rica	265	-0.09 (-0.21 to 0.03)
Cuba	188	0.56 (0.20 to 0.92)
DR Congo	120	-0.27 (-0.34 to -0.20)
Democratic Republic of Sao Tome and Principe	73	-0.18 (-0.28 to -0.09)
Djibouti	333	-0.66 (-0.74 to -0.58)
Dominica	45	-0.12 (-0.17 to -0.08)
Dominican Republic	934	5.27 (4.35 to 6.19)
Ecuador	173	-0.97 (-1.59 to -0.34)

Egypt	113	-0.20 (-0.34 to -0.06)
El Salvador	115	0.27 (0.03 to 0.52)
Equatorial Guinea	162	-0.05 (-0.13 to 0.04)
Eritrea	151	-0.34 (-0.39 to -0.29)
Ethiopia	146	-0.64 (-0.71 to -0.57)
Fiji	118	-0.24 (-0.38 to -0.11)
Gabon	75	-0.74 (-0.82 to -0.66)
Gambia	184	-0.40 (-0.53 to -0.26)
Georgia	309	6.46 (5.04 to 7.90)
Ghana	192	0.22 (0.07 to 0.37)
Grenada	132	-0.11 (-0.42 to 0.20)
Guatemala	186	0.56 (0.23 to 0.88)
Guinea	58	-0.95 (-1.04 to -0.86)
Guinea-Bissau	78	-0.27 (-0.37 to -0.18)
Haiti	142	0.29 (0.20 to 0.38)
Honduras	264	0.14 (0.00 to 0.28)
India	225	0.10 (0.00 to 0.20)
Indonesia	153	-0.28 (-0.34 to -0.22)
Iran	35	-2.67 (-2.85 to -2.48)
Iraq	357	2.20 (1.80 to 2.61)
Jamaica	127	0.88 (0.66 to 1.09)
Jordan	394	0.41 (-0.01 to 0.83)
Kazakhstan	115	1.15 (0.96 to 1.34)
Kenya	170	-0.50 (-0.55 to -0.44)
Kiribati	161	0.43 (0.28 to 0.58)
Kyrgyzstan	165	2.08 (1.25 to 2.92)
Laos	135	0.00 (-0.06 to 0.05)
Lebanon	123	-0.55 (-0.88 to -0.21)
Lesotho	44	-0.25 (-0.63 to 0.12)
Liberia	80	-0.69 (-0.83 to -0.56)
Libya	262	1.43 (1.11 to 1.76)
Madagascar	83	-0.84 (-0.91 to -0.76)
Malawi	78	-0.67 (-0.77 to -0.57)
Malaysia	207	-0.70 (-0.86 to -0.53)
Maldives	197	-0.19 (-0.35 to -0.02)
Mali	90	-1.00 (-1.07 to -0.93)
Marshall Islands	72	0.17 (0.00 to 0.34)
Mauritania	81	-0.80 (-0.86 to -0.75)
Mauritius	9288	6.89 (4.22 to 9.63)

Mexico	117	-1.50 (-1.65 to -1.35)
Micronesia	-32	-2.23 (-2.46 to -2.00)
Moldova	23	-0.77 (-1.30 to -0.24)
Mongolia	315	2.87 (2.41 to 3.34)
Montenegro	75	-0.59 (-0.69 to -0.49)
Morocco	216	1.43 (1.09 to 1.77)
Mozambique	40	-1.54 (-1.65 to -1.43)
Myanmar	93	-0.53 (-0.59 to -0.46)
Namibia	109	-0.82 (-0.96 to -0.67)
Nepal	175	-0.82 (-0.97 to -0.68)
Nicaragua	181	0.08 (-0.10 to 0.27)
Niger	150	-0.90 (-1.00 to -0.79)
Nigeria	102	-0.45 (-0.56 to -0.35)
North Korea	130	0.09 (0.01 to 0.17)
North Macedonia	24	-1.58 (-1.76 to -1.41)
Pakistan	114	-0.29 (-0.39 to -0.19)
Palestine	150	-0.59 (-0.67 to -0.51)
Papua New Guinea	248	-0.19 (-0.28 to -0.10)
Paraguay	360	2.33 (2.10 to 2.56)
Peru	495	1.50 (0.81 to 2.20)
Philippines	158	-0.53 (-0.67 to -0.39)
Rwanda	61	-0.96 (-1.06 to -0.85)
Saint Lucia	328	1.29 (0.47 to 2.11)
Saint Vincent and the Grenadines	553	2.91 (1.35 to 4.49)
Samoa	122	0.31 (0.17 to 0.45)
Senegal	159	-0.19 (-0.31 to -0.07)
Serbia	26	-1.03 (-1.12 to -0.93)
Sierra Leone	29	-1.17 (-1.35 to -0.98)
Solomon Islands	368	1.09 (1.01 to 1.17)
Somalia	203	-0.43 (-0.48 to -0.39)
South Africa	74	-1.61 (-1.97 to -1.25)
South Sudan	32	-0.88 (-1.00 to -0.76)
Sri Lanka	197	-0.58 (-0.79 to -0.37)
Sudan	267	1.65 (1.45 to 1.86)
Suriname	133	-0.24 (-0.35 to -0.13)
Swaziland	44	-1.54 (-1.86 to -1.22)
Syria	72	-1.32 (-1.45 to -1.19)
Tajikistan	246	1.77 (1.53 to 2.01)
Tanzania	113	-0.71 (-0.77 to -0.66)

Thailand	218	-0.98 (-1.18 to -0.78)
Timor-Leste	247	-0.83 (-0.93 to -0.74)
Togo	166	-0.54 (-0.66 to -0.42)
Tonga	27	-0.65 (-0.75 to -0.55)
Tunisia	50	-2.41 (-2.74 to -2.09)
Turkiye	82	-1.64 (-1.82 to -1.46)
Turkmenistan	3478	10.43 (9.33 to 11.54)
Tuvalu	65	0.26 (0.21 to 0.31)
Uganda	116	-0.76 (-0.83 to -0.70)
Ukraine	46	0.77 (0.58 to 0.97)
Uzbekistan	83	-0.74 (-1.45 to -0.02)
Vanuatu	250	0.27 (0.16 to 0.37)
Vietnam	155	-0.27 (-0.35 to -0.20)
Yemen	352	1.32 (1.10 to 1.53)
Zambia	105	-0.69 (-0.76 to -0.62)
Zimbabwe	100	-0.18 (-0.59 to 0.23)

Note: "--" indicates that estimates were not calculated due to a zero baseline in 1990 or insufficient data for regression.

Abbreviations: LMICs: Low- and Middle-Income Countries; PC: Percentage Change; EAPC: Estimated Annual Percentage Change; CI: Confidence Interval.

Table S13. Rheumatoid arthritis DALYs and age-standardized DALY rates in 129 LMICs, 1990

Location	DALYs (95% UI, 1990)	Age-standardized DALY rate (per 100,000, 95% UI, 1990)
Afghanistan	668.40 (421.28-949.00)	8.89 (5.61-12.67)
Albania	511.84 (368.93-681.99)	19.61 (14.15-26.16)
Algeria	1351.16 (886.02-1914.65)	8.14 (5.36-11.34)
Angola	1740.65 (1156.36-2523.10)	31.88 (20.90-44.93)
Argentina	9727.28 (7640.80-12702.21)	30.03 (23.58-39.29)
Armenia	352.58 (238.20-510.32)	11.28 (7.68-16.28)
Azerbaijan	603.35 (402.13-865.76)	9.85 (6.57-13.85)
Bangladesh	28075.99 (15671.39-54295.88)	57.83 (30.07-118.27)
Belarus	1485.52 (1037.11-2104.48)	12.18 (8.39-17.35)
Belize	23.41 (17.75-31.26)	21.27 (16.53-27.74)
Benin	443.74 (283.80-671.52)	16.73 (11.20-24.12)
Bhutan	100.71 (66.66-138.43)	34.80 (22.85-48.76)
Bolivia	1395.03 (987.48-1885.63)	34.31 (24.78-45.53)
Bosnia and Herzegovina	1318.43 (959.22-1714.34)	29.02 (21.38-37.61)
Botswana	262.45 (192.49-350.66)	32.95 (24.10-43.36)
Brazil	34515.31 (25026.76-47255.75)	28.87 (21.45-39.00)
Burkina Faso	1004.54 (622.78-1519.96)	18.74 (12.20-27.25)
Burundi	1545.63 (1062.87-2227.56)	47.04 (33.07-66.91)
Cabo Verde	31.22 (21.10-43.59)	14.04 (9.46-19.97)
Cambodia	682.62 (460.99-967.93)	11.73 (8.00-16.65)
Cameroon	1311.89 (829.36-1966.55)	22.19 (14.80-32.64)
Central African Republic	432.95 (292.65-630.43)	28.34 (19.17-40.37)
Chad	597.89 (372.34-914.66)	17.34 (11.24-25.98)
China	421034.87 (299352.27-539525.17)	43.91 (31.04-56.39)
Colombia	8072.72 (6458.04-10296.06)	38.16 (31.19-46.79)
Comoros	102.20 (74.92-134.85)	37.54 (28.06-49.71)
Congo (Brazzaville)	503.16 (344.06-707.99)	37.65 (25.19-53.15)
Costa Rica	855.05 (657.73-1150.46)	43.51 (34.25-57.21)
Cuba	2987.83 (2303.08-3905.00)	27.97 (21.76-36.34)
DR Congo	6454.61 (4166.44-9498.54)	31.07 (19.80-45.43)
Democratic Republic of Sao Tome and Principe	13.63 (9.18-19.95)	18.61 (12.68-26.60)
Djibouti	89.16 (64.37-119.24)	46.96 (34.63-62.75)
Dominica	12.81 (9.55-16.60)	19.36 (14.28-25.31)
Dominican Republic	542.53 (355.48-770.72)	11.16 (7.38-15.67)
Ecuador	2668.82 (2055.53-3487.97)	43.02 (33.74-54.00)

Egypt	4440.37 (2900.29-6057.19)	11.04 (7.30-15.02)
El Salvador	661.12 (466.38-884.46)	19.18 (13.67-25.89)
Equatorial Guinea	83.00 (52.91-120.49)	34.46 (21.82-48.84)
Eritrea	912.72 (630.64-1295.39)	49.79 (35.80-66.69)
Ethiopia	18426.65 (12249.51-26380.91)	63.70 (43.51-89.42)
Fiji	34.84 (21.33-52.66)	5.88 (3.71-8.60)
Gabon	223.57 (153.93-310.88)	35.88 (24.52-50.58)
Gambia	81.55 (52.10-120.32)	15.12 (10.32-21.43)
Georgia	698.08 (476.61-1006.12)	11.57 (7.90-16.73)
Ghana	1617.38 (954.32-2508.18)	18.83 (11.58-29.10)
Grenada	11.16 (8.46-14.69)	14.48 (11.05-19.05)
Guatemala	1608.83 (1331.99-2020.69)	33.32 (27.50-41.22)
Guinea	709.79 (440.85-1085.84)	18.28 (11.85-27.47)
Guinea-Bissau	116.72 (66.38-187.94)	19.87 (12.20-30.40)
Haiti	824.26 (514.95-1206.69)	19.25 (12.31-27.12)
Honduras	1325.35 (964.63-1781.97)	55.05 (38.71-73.25)
India	182035.93 (123985.63-252703.68)	38.10 (24.25-54.05)
Indonesia	9946.69 (6885.67-13577.25)	8.18 (5.60-11.14)
Iran	6497.81 (4593.89-8795.17)	18.40 (13.27-24.85)
Iraq	1254.10 (803.51-1812.76)	10.40 (6.84-14.63)
Jamaica	329.29 (250.28-441.13)	17.79 (13.55-23.87)
Jordan	251.11 (163.83-365.17)	10.76 (7.18-15.40)
Kazakhstan	1803.69 (1261.49-2536.95)	12.14 (8.55-16.83)
Kenya	4378.04 (3343.31-5736.62)	41.35 (31.44-52.62)
Kiribati	3.31 (2.03-4.84)	5.91 (3.66-8.55)
Kyrgyzstan	1187.89 (853.73-1624.54)	37.12 (27.28-49.91)
Laos	300.83 (207.37-424.79)	11.99 (8.11-16.88)
Lebanon	499.80 (338.36-677.66)	18.55 (12.57-25.27)
Lesotho	381.46 (280.65-518.32)	39.05 (28.94-52.21)
Liberia	279.23 (180.85-424.68)	18.33 (12.32-26.97)
Libya	290.85 (190.51-419.46)	10.34 (6.92-14.58)
Madagascar	3760.49 (2616.72-5197.44)	60.99 (42.43-82.06)
Malawi	2795.37 (1925.61-3906.31)	55.13 (39.28-75.48)
Malaysia	785.61 (507.32-1276.88)	6.62 (4.41-10.88)
Maldives	19.74 (13.55-27.64)	16.42 (11.50-22.26)
Mali	909.98 (559.67-1374.48)	17.90 (11.51-25.96)
Marshall Islands	1.86 (1.18-2.72)	7.07 (4.67-9.99)
Mauritania	261.85 (163.36-391.78)	21.45 (13.94-31.09)
Mauritius	78.32 (52.16-115.05)	8.69 (5.84-12.59)

Mexico	44603.26 (35902.42-55296.55)	89.56 (73.02-108.77)
Micronesia	5.81 (3.84-8.29)	8.42 (5.58-11.86)
Moldova	807.64 (623.18-1062.87)	17.42 (13.43-23.03)
Mongolia	216.09 (135.75-303.78)	15.81 (9.91-22.82)
Montenegro	147.30 (110.44-192.00)	23.50 (17.73-30.56)
Morocco	1797.23 (1144.20-2663.14)	9.45 (6.25-13.97)
Mozambique	3025.02 (2168.30-4030.15)	39.01 (28.36-51.28)
Myanmar	3674.27 (2469.53-5352.42)	13.65 (9.35-19.83)
Namibia	358.61 (256.06-467.43)	41.83 (30.55-54.34)
Nepal	3129.72 (2137.93-4257.16)	30.86 (20.91-42.54)
Nicaragua	704.59 (522.91-934.38)	37.31 (27.95-50.08)
Niger	717.15 (405.28-1106.02)	17.47 (10.79-25.87)
Nigeria	8755.39 (5524.92-12755.79)	15.86 (10.52-22.55)
North Korea	5865.29 (4369.54-7561.28)	33.43 (25.37-43.15)
North Macedonia	267.61 (178.91-368.46)	13.17 (8.82-18.14)
Pakistan	26692.96 (18457.07-35873.89)	40.68 (27.95-55.53)
Palestine	141.02 (91.48-196.64)	11.55 (7.69-15.95)
Papua New Guinea	160.50 (101.73-234.17)	5.61 (3.60-8.00)
Paraguay	949.39 (686.13-1275.07)	34.05 (24.69-45.84)
Peru	7220.48 (4930.80-9987.74)	44.66 (30.63-61.27)
Philippines	7735.52 (5618.76-10333.58)	20.55 (15.05-26.71)
Rwanda	2728.81 (1899.73-3840.88)	64.91 (45.55-89.65)
Saint Lucia	19.55 (14.65-25.10)	19.87 (14.94-25.17)
Saint Vincent and the Grenadines	8.45 (5.64-12.34)	10.49 (7.06-15.23)
Samoa	9.99 (6.46-14.16)	8.54 (5.47-12.11)
Senegal	764.57 (463.99-1153.78)	17.28 (11.33-25.02)
Serbia	2302.27 (1720.75-3026.12)	21.19 (15.86-27.77)
Sierra Leone	939.44 (576.99-1460.47)	42.32 (24.73-69.72)
Solomon Islands	15.07 (9.51-22.13)	6.99 (4.53-10.12)
Somalia	1329.08 (927.74-1965.71)	35.35 (25.05-52.72)
South Africa	18061.43 (13318.00-23752.35)	68.74 (51.35-88.46)
South Sudan	1777.21 (1212.41-2518.28)	58.65 (39.85-83.75)
Sri Lanka	909.86 (616.84-1311.84)	6.67 (4.63-9.32)
Sudan	1071.49 (707.66-1441.94)	8.04 (5.33-11.09)
Suriname	35.27 (23.10-50.83)	11.60 (7.69-17.49)
Swaziland	230.73 (166.88-306.17)	53.29 (38.83-68.34)
Syria	718.90 (436.17-1067.31)	8.93 (5.51-13.11)
Tajikistan	650.08 (444.65-855.49)	19.06 (13.29-24.58)
Tanzania	6416.86 (4685.29-8377.58)	44.96 (32.79-59.09)

Thailand	6554.61 (4617.18-9264.15)	15.35 (10.97-21.95)
Timor-Leste	45.36 (31.19-63.46)	11.99 (8.05-17.57)
Togo	344.68 (202.67-550.48)	18.31 (11.82-27.16)
Tonga	5.59 (3.70-7.92)	7.97 (5.23-11.36)
Tunisia	1045.44 (742.34-1467.13)	17.80 (12.35-25.33)
Turkiye	12916.94 (9606.93-17622.46)	30.25 (22.48-41.74)
Turkmenistan	234.24 (153.13-333.74)	9.03 (5.98-13.28)
Tuvalu	0.56 (0.35-0.82)	6.93 (4.44-10.04)
Uganda	3662.93 (2634.64-4986.98)	43.44 (31.68-59.35)
Ukraine	9235.04 (6944.34-11929.50)	14.13 (10.56-18.34)
Uzbekistan	6485.07 (5019.48-8450.45)	47.52 (36.98-60.77)
Vanuatu	6.76 (4.28-9.72)	6.69 (4.38-9.43)
Vietnam	6616.86 (4653.50-8801.61)	14.44 (10.23-19.24)
Yemen	568.48 (369.12-791.99)	7.40 (4.85-10.27)
Zambia	2291.70 (1634.58-3128.79)	60.49 (43.81-80.77)
Zimbabwe	1429.40 (1031.64-1905.00)	25.26 (18.65-33.22)

Notes: PC was calculated from all-age counts between 1990 and 2023, whereas EAPC was calculated from age-standardized rates over the 1990-2023 time series. Abbreviations: LMICs: Low- and Middle-Income Countries; DALY: Disability-Adjusted Life-Year; UI: Uncertainty Interval.

Table S14. Rheumatoid arthritis DALYs and age-standardized DALY rates in 129 LMICs, 2023

Location	DALYs (95% UI, 2023)	Age-standardized DALY rate (per 100,000, 95% UI, 2023)
Afghanistan	2619.05 (1696.87-3797.05)	12.02 (7.91-16.89)
Albania	927.32 (650.03-1266.85)	25.15 (17.56-35.08)
Algeria	5743.70 (3981.22-7807.95)	12.74 (8.84-17.27)
Angola	5846.45 (4284.39-7975.98)	31.34 (22.73-41.05)
Argentina	21076.59 (15608.17-27815.75)	37.76 (27.76-50.28)
Armenia	793.80 (570.23-1043.09)	18.91 (13.32-25.18)
Azerbaijan	1532.70 (1051.66-2202.29)	12.64 (8.62-18.29)
Bangladesh	66627.13 (44499.32-104519.89)	51.32 (32.34-84.91)
Belarus	2526.20 (1749.59-3523.97)	18.27 (12.46-26.00)
Belize	105.12 (78.26-143.16)	28.73 (21.62-38.50)
Benin	1442.28 (930.89-2036.24)	17.45 (11.93-24.01)
Bhutan	295.22 (194.26-412.14)	42.29 (28.05-57.80)
Bolivia	5993.48 (4337.38-8085.73)	55.19 (40.59-73.40)
Bosnia and Herzegovina	1966.79 (1433.74-2609.66)	38.24 (27.57-50.93)
Botswana	730.82 (535.26-984.13)	35.30 (26.14-46.99)
Brazil	69669.09 (51203.52-93071.84)	27.79 (20.25-37.35)
Burkina Faso	2358.54 (1556.60-3277.04)	17.11 (11.82-23.37)
Burundi	2902.13 (2108.18-3883.30)	39.75 (28.94-54.38)
Cabo Verde	70.71 (49.46-95.72)	15.14 (10.74-20.41)
Cambodia	2169.71 (1506.83-2923.23)	14.32 (9.98-19.18)
Cameroon	4205.30 (2712.36-6230.07)	20.68 (13.81-29.54)
Central African Republic	888.90 (605.83-1309.64)	25.26 (17.71-36.90)
Chad	1506.44 (912.16-2263.04)	15.82 (10.31-22.61)
China	837107.74 (617299.44-1068527.32)	40.21 (29.34-52.10)
Colombia	24821.01 (18624.46-32537.35)	42.25 (31.62-55.48)
Comoros	213.70 (159.66-283.44)	32.76 (24.54-43.69)
Congo (Brazzaville)	1260.82 (934.95-1707.01)	35.54 (26.82-46.99)
Costa Rica	3698.14 (2769.32-4872.73)	60.61 (45.38-79.85)
Cuba	6793.43 (5278.83-8613.15)	39.55 (30.10-51.33)
DR Congo	14860.19 (10633.77-21019.06)	28.81 (20.80-40.04)
Democratic Republic of Sao Tome and Principe	31.81 (21.66-46.15)	19.39 (13.75-27.00)
Djibouti	378.24 (286.88-501.72)	42.31 (31.73-56.56)
Dominica	20.06 (14.32-26.52)	22.68 (15.95-30.37)
Dominican Republic	1888.07 (1332.31-2604.97)	17.65 (12.51-24.36)
Ecuador	10186.67 (7653.65-13510.43)	56.26 (42.34-74.46)

Egypt	16116.48 (11222.94-22627.97)	16.89 (11.82-23.41)
El Salvador	1652.91 (1149.88-2238.39)	26.41 (18.48-35.49)
Equatorial Guinea	301.79 (217.91-419.51)	39.47 (28.84-53.28)
Eritrea	2076.90 (1497.61-2867.82)	46.68 (33.54-63.18)
Ethiopia	35902.41 (25937.22-47480.90)	51.19 (36.92-68.90)
Fiji	69.22 (44.24-102.56)	7.41 (4.78-10.90)
Gabon	524.28 (387.93-703.86)	37.00 (27.30-49.32)
Gambia	254.94 (166.09-355.51)	16.30 (11.26-22.23)
Georgia	710.27 (495.86-960.86)	14.89 (10.22-20.52)
Ghana	4812.24 (3203.92-7509.99)	21.04 (14.40-31.53)
Grenada	29.15 (22.08-38.60)	20.48 (15.30-27.34)
Guatemala	5211.71 (3973.74-6906.35)	40.58 (31.70-52.71)
Guinea	1458.03 (915.81-2149.79)	17.21 (11.26-24.05)
Guinea-Bissau	252.06 (154.89-383.95)	19.45 (12.97-28.18)
Haiti	2246.30 (1501.66-3180.08)	22.22 (15.12-31.61)
Honduras	5676.99 (4111.45-7606.90)	71.48 (52.30-94.74)
India	597363.73 (406058.73-793743.84)	46.63 (31.49-63.27)
Indonesia	27825.02 (18976.99-38372.32)	9.54 (6.52-13.27)
Iran	15666.23 (11022.35-21150.40)	16.24 (11.48-22.24)
Iraq	5558.30 (3705.01-8188.08)	14.85 (10.03-21.18)
Jamaica	795.11 (607.09-1034.10)	25.07 (19.14-32.75)
Jordan	1956.32 (1269.39-2807.13)	15.83 (10.47-22.27)
Kazakhstan	3553.76 (2474.72-4874.48)	16.77 (11.72-22.97)
Kenya	11852.60 (8878.51-15655.31)	37.25 (28.16-49.34)
Kiribati	7.90 (5.01-11.60)	7.13 (4.54-10.27)
Kyrgyzstan	3387.32 (2542.98-4570.05)	55.67 (42.10-73.69)
Laos	891.48 (601.26-1224.00)	15.13 (10.23-21.09)
Lebanon	1544.95 (1063.49-2150.14)	25.22 (17.33-34.85)
Lesotho	603.49 (442.40-810.25)	41.00 (30.22-54.85)
Liberia	705.99 (460.15-1049.27)	18.12 (12.42-25.28)
Libya	1142.03 (781.05-1579.77)	14.64 (10.04-20.18)
Madagascar	7523.38 (5462.68-10217.08)	48.23 (34.82-66.22)
Malawi	5087.73 (3748.93-6968.34)	47.47 (35.15-64.62)
Malaysia	2892.03 (1915.86-4417.52)	8.18 (5.47-12.61)
Maldives	81.61 (56.30-111.79)	17.89 (13.00-23.38)
Mali	2127.47 (1330.91-3064.35)	15.71 (10.36-21.62)
Marshall Islands	3.40 (2.18-4.97)	9.35 (6.10-13.54)
Mauritania	623.31 (418.54-899.72)	21.55 (15.09-30.42)
Mauritius	347.10 (265.54-453.00)	19.85 (15.05-26.08)

Mexico	122562.17 (96194.13-154471.06)	86.23 (67.91-108.19)
Micronesia	10.98 (7.04-15.67)	11.24 (7.25-16.01)
Moldova	1167.05 (871.63-1537.69)	21.74 (16.11-29.08)
Mongolia	751.66 (537.71-1012.54)	24.17 (17.47-32.16)
Montenegro	249.89 (181.37-331.61)	26.56 (18.90-35.81)
Morocco	5733.69 (3843.72-7943.33)	14.32 (9.64-19.77)
Mozambique	5154.96 (3817.64-6759.47)	31.03 (23.45-40.90)
Myanmar	8630.18 (5890.23-11698.66)	16.20 (11.25-22.25)
Namibia	879.53 (623.93-1147.77)	40.97 (29.43-52.30)
Nepal	10347.20 (7070.97-13890.59)	37.88 (25.80-51.60)
Nicaragua	2500.33 (1804.38-3361.48)	44.42 (32.49-58.98)
Niger	2092.13 (1242.97-3334.12)	16.16 (10.14-23.35)
Nigeria	23599.48 (15764.56-33240.14)	16.63 (11.55-22.87)
North Korea	13336.66 (9886.59-17806.69)	39.74 (29.43-52.96)
North Macedonia	479.71 (324.04-687.18)	18.08 (12.18-25.85)
Pakistan	53488.72 (35914.70-72902.37)	33.30 (21.75-45.90)
Palestine	641.71 (420.42-892.83)	15.40 (10.35-21.28)
Papua New Guinea	585.64 (368.67-873.49)	6.71 (4.27-9.74)
Paraguay	3747.83 (2862.12-5000.70)	60.75 (46.66-80.91)
Peru	29039.09 (20732.63-39271.83)	76.06 (54.55-102.84)
Philippines	17263.58 (12185.37-23487.82)	18.27 (13.24-24.54)
Rwanda	4175.56 (3084.28-5732.78)	49.27 (35.73-67.66)
Saint Lucia	65.77 (49.06-85.28)	28.08 (20.74-36.55)
Saint Vincent and the Grenadines	20.91 (14.28-28.10)	14.71 (9.93-19.91)
Samoa	19.90 (13.32-28.52)	10.86 (7.27-15.70)
Senegal	2114.78 (1367.03-3006.13)	18.05 (12.00-24.90)
Serbia	3788.89 (2705.20-4914.07)	26.57 (18.70-35.23)
Sierra Leone	1474.03 (1033.13-2142.48)	34.79 (23.94-53.82)
Solomon Islands	63.93 (40.47-94.08)	9.40 (6.11-13.57)
Somalia	3679.89 (2554.40-5073.38)	32.75 (23.13-44.06)
South Africa	32621.31 (23513.11-43117.35)	52.40 (38.08-67.81)
South Sudan	2717.71 (1923.59-3712.61)	49.31 (34.95-70.81)
Sri Lanka	2372.62 (1618.74-3273.82)	8.69 (5.85-12.09)
Sudan	3852.36 (2534.03-5284.86)	12.35 (8.15-16.94)
Suriname	92.26 (62.51-133.04)	14.13 (9.57-20.50)
Swaziland	399.12 (286.54-540.92)	44.01 (32.01-58.77)
Syria	2492.80 (1647.05-3599.86)	14.16 (9.39-20.25)
Tajikistan	1999.20 (1417.11-2740.59)	25.44 (18.57-34.04)
Tanzania	14250.26 (10455.31-18940.68)	38.73 (29.07-51.45)

Thailand	18872.86 (13642.48-25792.01)	18.28 (13.17-24.80)
Timor-Leste	135.93 (92.17-187.56)	13.48 (9.21-18.19)
Togo	1018.60 (666.59-1462.97)	17.64 (11.79-25.15)
Tonga	9.56 (6.01-13.89)	10.45 (6.58-15.18)
Tunisia	2560.76 (1832.26-3513.76)	18.38 (13.17-25.11)
Turkiye	35711.55 (26162.91-49153.61)	35.48 (26.01-48.96)
Turkmenistan	734.57 (511.28-1063.24)	14.06 (9.89-20.14)
Tuvalu	0.98 (0.64-1.41)	9.46 (6.19-13.82)
Uganda	9025.93 (6732.40-12441.64)	39.97 (30.05-53.43)
Ukraine	12115.81 (9130.73-16005.11)	18.98 (14.17-25.16)
Uzbekistan	16036.74 (11695.78-21746.91)	48.28 (35.65-64.87)
Vanuatu	22.82 (14.57-34.30)	8.54 (5.52-12.70)
Vietnam	23515.49 (16720.50-32207.75)	20.63 (14.66-28.26)
Yemen	2483.52 (1704.86-3606.55)	9.99 (7.06-13.97)
Zambia	5135.90 (3789.37-7000.30)	53.09 (38.64-70.10)
Zimbabwe	2895.36 (2115.46-3828.06)	26.10 (19.06-34.63)

Abbreviations: LMICs: Low- and Middle-Income Countries; DALY: Disability-Adjusted Life-Year; UI: Uncertainty Interval.

Table S15. Percentage change in DALYs and EAPC in age-standardized DALY rates of rheumatoid arthritis in 129 LMICs, 1990–2023

Location	DALYs PC (%, 1990–2023)	DALYs EAPC (%, 95% CI, 1990–2023)
Afghanistan	292	1.10 (1.01 to 1.19)
Albania	81	1.00 (0.90 to 1.10)
Algeria	325	1.53 (1.46 to 1.60)
Angola	236	0.11 (0.02 to 0.20)
Argentina	117	0.79 (0.64 to 0.95)
Armenia	125	1.97 (1.75 to 2.20)
Azerbaijan	154	1.04 (0.81 to 1.26)
Bangladesh	137	-0.46 (-0.51 to -0.41)
Belarus	70	1.29 (1.18 to 1.40)
Belize	349	0.91 (0.81 to 1.01)
Benin	225	0.06 (-0.03 to 0.15)
Bhutan	193	0.61 (0.53 to 0.69)
Bolivia	330	1.55 (1.48 to 1.62)
Bosnia and Herzegovina	49	1.01 (0.93 to 1.08)
Botswana	178	0.19 (0.09 to 0.28)
Brazil	102	0.06 (-0.03 to 0.16)
Burkina Faso	135	-0.16 (-0.23 to -0.08)
Burundi	88	-0.54 (-0.61 to -0.46)
Cabo Verde	127	0.40 (0.27 to 0.53)
Cambodia	218	0.59 (0.54 to 0.64)
Cameroon	221	-0.21 (-0.35 to -0.06)
Central African Republic	105	-0.53 (-0.61 to -0.45)
Chad	152	-0.24 (-0.33 to -0.16)
China	99	-0.22 (-0.27 to -0.17)
Colombia	207	0.55 (0.41 to 0.68)
Comoros	109	-0.47 (-0.51 to -0.42)
Congo (Brazzaville)	151	-0.13 (-0.20 to -0.07)
Costa Rica	333	0.96 (0.89 to 1.03)
Cuba	127	0.88 (0.80 to 0.95)
DR Congo	130	-0.10 (-0.20 to 0.00)
Democratic Republic of Sao Tome and Principe	133	0.26 (0.13 to 0.39)
Djibouti	324	-0.37 (-0.48 to -0.26)
Dominica	57	0.42 (0.38 to 0.46)
Dominican Republic	248	1.56 (1.39 to 1.73)
Ecuador	282	0.71 (0.46 to 0.96)

Egypt	263	1.28 (1.22 to 1.34)
El Salvador	150	1.11 (1.07 to 1.14)
Equatorial Guinea	264	0.67 (0.57 to 0.77)
Eritrea	128	-0.18 (-0.23 to -0.13)
Ethiopia	95	-0.82 (-0.88 to -0.75)
Fiji	99	0.73 (0.62 to 0.85)
Gabon	134	0.18 (0.15 to 0.22)
Gambia	213	0.23 (0.14 to 0.32)
Georgia	2	0.94 (0.81 to 1.07)
Ghana	198	0.45 (0.32 to 0.58)
Grenada	161	0.80 (0.63 to 0.96)
Guatemala	224	0.90 (0.76 to 1.05)
Guinea	105	-0.25 (-0.35 to -0.15)
Guinea-Bissau	116	0.01 (-0.14 to 0.16)
Haiti	173	0.48 (0.43 to 0.53)
Honduras	328	0.80 (0.72 to 0.89)
India	228	0.70 (0.62 to 0.78)
Indonesia	180	0.46 (0.44 to 0.49)
Iran	141	-0.46 (-0.55 to -0.37)
Iraq	343	1.34 (1.18 to 1.50)
Jamaica	141	0.96 (0.86 to 1.06)
Jordan	679	1.52 (1.40 to 1.64)
Kazakhstan	97	1.22 (1.07 to 1.38)
Kenya	171	-0.29 (-0.34 to -0.25)
Kiribati	139	0.52 (0.46 to 0.57)
Kyrgyzstan	185	1.62 (1.33 to 1.90)
Laos	196	0.71 (0.67 to 0.74)
Lebanon	209	1.10 (0.96 to 1.24)
Lesotho	58	0.06 (-0.18 to 0.30)
Liberia	153	0.11 (-0.02 to 0.24)
Libya	293	1.30 (1.19 to 1.40)
Madagascar	100	-0.78 (-0.88 to -0.68)
Malawi	82	-0.53 (-0.61 to -0.45)
Malaysia	268	0.68 (0.63 to 0.72)
Maldives	313	0.42 (0.22 to 0.62)
Mali	134	-0.35 (-0.44 to -0.26)
Marshall Islands	82	0.85 (0.79 to 0.91)
Mauritania	138	0.01 (-0.05 to 0.06)
Mauritius	343	1.95 (1.49 to 2.42)

Mexico	175	-0.16 (-0.23 to -0.10)
Micronesia	89	0.83 (0.78 to 0.88)
Moldova	45	0.65 (0.43 to 0.88)
Mongolia	248	1.79 (1.61 to 1.97)
Montenegro	70	0.55 (0.46 to 0.63)
Morocco	219	1.42 (1.37 to 1.47)
Mozambique	70	-0.86 (-0.94 to -0.77)
Myanmar	135	0.48 (0.42 to 0.53)
Namibia	145	-0.10 (-0.19 to -0.01)
Nepal	231	0.58 (0.49 to 0.68)
Nicaragua	255	0.77 (0.70 to 0.83)
Niger	192	-0.32 (-0.43 to -0.20)
Nigeria	170	0.20 (0.07 to 0.32)
North Korea	127	0.61 (0.58 to 0.65)
North Macedonia	79	1.12 (1.03 to 1.20)
Pakistan	100	-0.16 (-0.30 to -0.02)
Palestine	355	0.89 (0.79 to 0.98)
Papua New Guinea	265	0.47 (0.41 to 0.54)
Paraguay	295	1.73 (1.61 to 1.85)
Peru	302	1.81 (1.76 to 1.85)
Philippines	123	-0.31 (-0.35 to -0.27)
Rwanda	53	-1.12 (-1.29 to -0.94)
Saint Lucia	236	0.96 (0.64 to 1.28)
Saint Vincent and the Grenadines	147	1.04 (0.91 to 1.17)
Samoa	99	0.64 (0.59 to 0.69)
Senegal	177	0.04 (-0.04 to 0.12)
Serbia	65	0.74 (0.63 to 0.85)
Sierra Leone	57	-0.73 (-0.92 to -0.54)
Solomon Islands	324	0.84 (0.80 to 0.89)
Somalia	177	-0.28 (-0.30 to -0.26)
South Africa	81	-0.83 (-0.95 to -0.72)
South Sudan	53	-0.58 (-0.71 to -0.45)
Sri Lanka	161	0.87 (0.79 to 0.94)
Sudan	260	1.52 (1.42 to 1.62)
Suriname	162	0.69 (0.65 to 0.74)
Swaziland	73	-0.72 (-0.86 to -0.58)
Syria	247	1.53 (1.44 to 1.62)
Tajikistan	208	0.81 (0.74 to 0.87)
Tanzania	122	-0.46 (-0.50 to -0.43)

Thailand	188	0.52 (0.48 to 0.56)
Timor-Leste	200	0.32 (0.26 to 0.38)
Togo	196	-0.02 (-0.16 to 0.12)
Tonga	71	0.73 (0.67 to 0.79)
Tunisia	145	0.08 (0.04 to 0.11)
Turkiye	176	0.59 (0.50 to 0.67)
Turkmenistan	214	1.66 (1.46 to 1.86)
Tuvalu	75	0.91 (0.88 to 0.94)
Uganda	146	-0.42 (-0.48 to -0.35)
Ukraine	31	0.76 (0.71 to 0.82)
Uzbekistan	147	0.01 (-0.15 to 0.18)
Vanuatu	237	0.74 (0.70 to 0.78)
Vietnam	255	1.11 (1.05 to 1.16)
Yemen	337	1.08 (1.01 to 1.14)
Zambia	124	-0.43 (-0.51 to -0.35)
Zimbabwe	103	0.10 (-0.14 to 0.34)

Notes: PC was calculated from all-age counts between 1990 and 2023, whereas EAPC was calculated from age-standardized rates over the 1990-2023 time series. Abbreviations: LMICs: Low- and Middle-Income Countries; DALY: Disability-Adjusted Life-Year; PC: Percentage Change; EAPC: Estimated Annual Percentage Change; CI: Confidence Interval; DALYs: Disability-Adjusted Life-Years.

Table S16. Income-stratified projections of all-age rheumatoid arthritis burden rates in 129 LMICs, 2023–2050

Measures	Location	2023 Baseline	2050 Projection	Change (%)
**DALYs**				
	GNI-L	23.9 (17.1–32.8)	26.6 (6.2–47.1)	11.3%
	GNI-LM	32.3 (22.1–43.9)	33.4 (9.0–57.9)	3.3%
	GNI-UM	49.5 (36.6–64.1)	64.2 (60.2–68.2)	29.6%
**Deaths**				
	GNI-L	0.51 (0.33–0.76)	0.44 (0.39–0.50)	-12.8%
	GNI-LM	0.58 (0.27–0.99)	0.74 (0.65–0.82)	26.8%
	GNI-UM	0.54 (0.36–0.72)	0.66 (0.55–0.77)	22.7%
**Incidence**				
	GNI-L	4.72 (4.07–5.46)	4.18 (2.22–6.14)	-11.4%
	GNI-LM	10.44 (9.17–11.94)	--	--
	GNI-UM	14.84 (13.01–16.96)	19.12 (18.33–19.91)	28.8%
**Prevalence**				
	GNI-L	70.7 (59.3–83.1)	65.4 (32.0–98.9)	-7.4%
	GNI-LM	155.5 (134.7–182.5)	--	--
	GNI-UM	290.7 (254.1–337.0)	358.5 (244.0–472.9)	23.3%

Notes: Values represent rates per 100,000 population. Baseline 2023 GBD estimates are shown with 95% uncertainty intervals, whereas 2050 ARIMA projections are shown with 95% prediction intervals. Percentage changes were calculated from unrounded point estimates. Countries were stratified by World Bank 2025 gross national income (GNI) per capita. Abbreviations: LMICs, low- and middle-income countries; GNI-L, low-income countries; GNI-LM, lower-middle-income countries; GNI-UM, upper-middle-income countries; DALYs, disability-adjusted life years; UI, uncertainty interval. "--" indicates that 2050 point estimates and percentage changes were not summarized because the forecast crossed below zero over the long-term horizon.
